# Structural Diversity and Differential Natural Pairings of MAT1-1-1 and MAT1-2-1 Proteins Essential for Sexual Reproduction in *Ophiocordyceps sinensis* Strains

**DOI:** 10.3390/ijms27167191

**Published:** 2026-08-11

**Authors:** Xiu-Zhang Li, Yu-Ling Li, Wei Liu, Jian-Zhao Qi, Jia-Shi Zhu

**Affiliations:** 1 State Key Laboratory of Plateau Ecology and Agriculture, Qinghai Academy of Animal and Veterinary Sciences, Qinghai University, Xining 810016, China; xiuzhang11@163.com (X.-Z.L.); yulingli2000@163.com (Y.-L.L.); 2 Qinghai Provincial Grassland Engineering Technology Research Center, Xining 810016, China; 3 Institute of Immunology, Army Medical University, Chongqing 400038, China; weiliu@tmmu.edu.cn; 4 College of Chemistry and Pharmacy, Northwest A&F University, Yangling 712100, China; qjz@nwafu.edu.cn

**Keywords:** MATα_HMGbox domain of the MAT1-1-1 protein, HMG-box_ROX1-like domain of the MAT1-2-1 protein, heteromorphic primary, secondary, and tertiary structures of proteins, *Ophiocordyceps sinensis* strain, *Cordyceps sinensis* insect–fungal complex, sexual reproduction, heterothallism, hybridization

## Abstract

The MAT1-1-1 and MAT1-2-1 proteins perform essential DNA-binding activities and regulation of the transcription of genes governing sexual reproduction in *Ophiocordyceps sinensis*. Previous studies have documented differential occurrence, alternative splicing, and transcriptional divergence of *MAT1-1-1*, *MAT1-2-1*, and pheromone receptor genes in *Hirsutella sinensis* (Genotype #1 among 17 genome-independent genotypes of *O. sinensis* fungi). In the present study, we analyzed the natural pairing patterns of structurally variant MAT1-1-1 and MAT1-2-1 proteins simultaneously produced by each of 20 purportedly homogenous *O. sinensis* strains, based on AlphaFold-predicted 3D structural models and pairwise structural superposition analyses. The differentially naturally paired mating proteins exhibited distinct heteromorphic stereostructures across strains. Specifically, the MATα_HMGbox domain of MAT1-1-1 and the HMG-box_ROX1-like domain of MAT1-2-1 displayed variable N- and/or C-terminal truncations, 1–4 amino acid substitutions at distinct sites, and concomitant alterations in hydrophobic properties and secondary/tertiary structural configurations. Thus, the differentially naturally paired but structurally divergent mating proteins support heterogeneous fungal origins within the analyzed *O. sinensis* strains and are inconsistent with a strictly self-fertilization reproductive model. Our findings suggest that *O. sinensis* adopts a self-sterile reproductive strategy, potentially involving heterothallic mating or hybrid reproduction during the lifecycle of the LEVEL-II protected *Cordyceps sinensis* insect–fungal complex endemic to the Qinghai-Tibet Plateau.

## 1. Introduction

Natural *Cordyceps sinensis* (DōngChóng XiàCǎo, **冬虫夏草,** in Chinese) is found exclusively in alpine meadows (3000–5000 m) on the Qinghai-Tibet Plateau, a restricted habitat that contributes substantially to its scarcity. It has been used for centuries in traditional Chinese medicine for health maintenance, disease amelioration, post-illness and postoperative recovery, and antiaging therapy [1,2,3,4]. Recent studies have investigated the complex biochemical mechanisms that may underlie these clinical applications, including the regulation of glucose and lipid metabolism, immunomodulatory effects, anti-inflammatory and antioxidant effects, hormone-like activities related to libido and fertility, and improvements in perceived energy/vitality. This distinctive therapeutic significance, together with the high commercial value of this natural substance, has driven extensive overharvesting and led to its designation as a LEVEL-II protected natural substance in China [5].

Natural *C. sinensis* comprises the fruiting body of *Ophiocordyceps sinensis* and the remains of a mummified *Hepialidae* moth larva. The larval body retains a thick body wall bearing numerous bristles, together with intact intestinal structures, cephalic tissues, and fragments of other tissues [6,7,8,9,10,11,12,13]. Dual-fluorescence microscopy has revealed multicellular heterokaryotic microstructures within *C. sinensis* ascospores and hyphae, including mono-, bi-, tri-, and tetra-nucleate cells [14]. Molecular studies have further demonstrated substantial genetic heterogeneity, identifying >90 fungal species across at least 37 genera, as well as 17 genomically independent *O. sinensis* genotypes that differentially co-occur and vary in natural abundance among distinct compartments of *C. sinensis* insect-fungal complex during maturation, which supports their genome-independent natural characteristics [9,10,12,13,15,16,17,18,19,20,21,22,23,24,25,26,27,28,29,30,31,32,33,34,35,36,37,38,39,40,41,42,43,44]. Accordingly, the Chinese Pharmacopoeia defines this medicinal microecosystem as an insect-fungal complex.

Among the numerous co-occurring fungal species [20,31,32,33,40,41], *Hirsutella sinensis* has been proposed as the sole anamorph of *O. sinensis* [45]. However, this hypothesis has not yet been conclusively validated according to all 4 criteria of Koch’s postulates. Among the many attempts to support this hypothesis, Wei et al. [46] reported successful artificial cultivation in an industrial, product-oriented setting. They revealed a discrepancy between the species identities of the anamorphic inoculants (3 GC-biased Genotype #1 *H. sinensis* strains) introduced into *Hepialidae* larvae and the sole teleomorph (AT-biased Genotype #4 of *O. sinensis*) identified in the cultivated fruiting body.

Since the 1840s, the Latin designation *C. sinensis* has been used indiscriminately to refer to both the fungal teleomorph and the wild insect-fungal complex, despite Edward Doubleday’s original finding in 1842 that the insect component of the natural material belonged to *Agrotis* [47]. This imprecise terminology has blurred the distinction among the fungus, the host insect, and the insect–fungal complex as a unique therapeutic entity. Consequently, inconsistencies have emerged across scientific publications, commercial trade in the mass market, and governmental regulatory frameworks worldwide. In 2007, the fungus was renamed *O. sinensis*, and the *H. sinensis* strain EFCC7287 (GC-biased Genotype #1 of *O. sinensis*) was designated the nomenclatural reference [9,10,48,49,50,51]. Following the “One Fungus = One Name” nomenclature principle established by the International Mycological Association (IMA) [52,53], Zhang et al. [51] interpreted *O. sinensis* as a single fungal species to satisfy the “One Fungus” prerequisite of the rule and replaced the anamorphic name *H. sinensis* with the teleomorphic name *O. sinensis*. However, the 17 genomically independent genotypes that differentially coexist in various combinations within different compartments of the *C. sinensis* insect–fungal complex were not fully considered. In the present study, we retain the anamorphic designation *H. sinensis* specifically for GC-biased Genotype #1 while referring to the genome-independent Genotypes #2–17 fungi as *O. sinensis* in accordance with annotations in public databases such as GenBank and AlphaFold, pending formal taxonomic resolution. For consistency with the longstanding practice in the medicinal literature, we also retain the term *C. sinensis* to denote both wild and cultivated insect-fungal complexes, acknowledging that the 2007 nomenclatural revision did not resolve the longstanding indiscriminate application of the Latin name *C. sinensis* to the insect-fungal complex [9,10,48,51,54]. This provisionally necessary practice may require future refinement, as proprietary and exclusive Latin names are established for individual genome-independent genotypes of *O. sinensis* and for the insect-fungal complex as a distinct therapeutic entity.

Sexual reproduction in ascomycetes is regulated by transcription factors encoded at the mating-type (*MAT*) locus, which mediate mating-type recognition and compatibility, as well as the development of fruiting bodies and sexual structures (ascocarps and ascospores) [55,56,57,58,59,60,61,62]. In *O. sinensis*, reproductive behavior depends on the coordinated activity of 2 core mating proteins encoded by the *MAT1-1* and *MAT1-2* idiomorphs. The MAT1-1-1 protein contains a mating-type alpha high mobility group box (MATα_HMGbox) domain, whereas the MAT1-2-1 protein contains a high mobility group box ROX1-like (HMG-box_ROX1-like) domain [14,58,63,64,65]. Each DNA-binding domain contains a hydrophobic core formed by 3 asymmetrically oriented α-helices. These DNA-binding domains regulate the transcription of genes associated with sexual reproduction. Li et al. [65,66,67,68] reported differential occurrence, alternative splicing, and differential transcription of mating-type and pheromone receptor genes in the genome and transcriptome assemblies of 7 *H. sinensis* strains, which are inconsistent with self-fertilization but consistent with self-sterility. In addition, Li et al. [65,69] described differential occurrence of the MAT1-1-1 and MAT1-2-1 proteins with heteromorphic tertiary structures of the DNA-binding domains in wild-type *C. sinensis* isolates, which may have originated from either GC-biased Genotype #1 or Genotype #3 of *O. sinensis*. Taken together, these observations support the existence of complex reproductive biology in *O. sinensis* that may involve heterothallic mating or hybrid reproductive mechanisms.

Variations in the DNA-binding domains of the MAT1-1-1 and MAT1-2-1 proteins with diverse amino acid substitutions may alter their tertiary structures and influence the interactions between the critical mating proteins. Prior studies have characterized mating proteins from numerous impure wild-type *C. sinensis* isolates characterized by Prof. Zhang Y-J and associates [7,51,65,70,71,72]. In the present study, we investigated the naturally paired MAT1-1-1 and MAT1-2-1 proteins simultaneously coproduced by each of 20 purportedly pure *O. sinensis* strains reported by Prof. Yao Y-J and collaborators [14,36]. Specifically, we analyzed differential N- and/or C-terminal truncations and diverse amino acid substitutions within the MATα_HMGbox and HMG-box_ROX1-like domains and assessed how these variants are naturally paired yet structurally divergent within individual strains. These analyses enable the evaluation of whether the natural pairings and structural diversity of the mating proteins are consistent with their derivation from a single homogeneous fungal source or instead suggest contributions from multiple co-occurring and symbiotic fungal taxa within the purportedly pure, but potentially impure, strains.

## 2. Results

### 2.1. Primary Structures of the Truncated MAT1-1-1 Proteins in O. sinensis Strains

A sequence alignment of 20 MAT1-1-1 proteins (AGW27517–AGW27536 [14]), exhibiting variable N- and C-terminal truncations together with 1–4 amino acid substitutions at distinct sites, and the representative full-length authentic MAT1-1-1 protein AGW27560 derived from the *H. sinensis* strain CS68-2-1229 is presented in Figure S1 [14].

**Figure 1:**
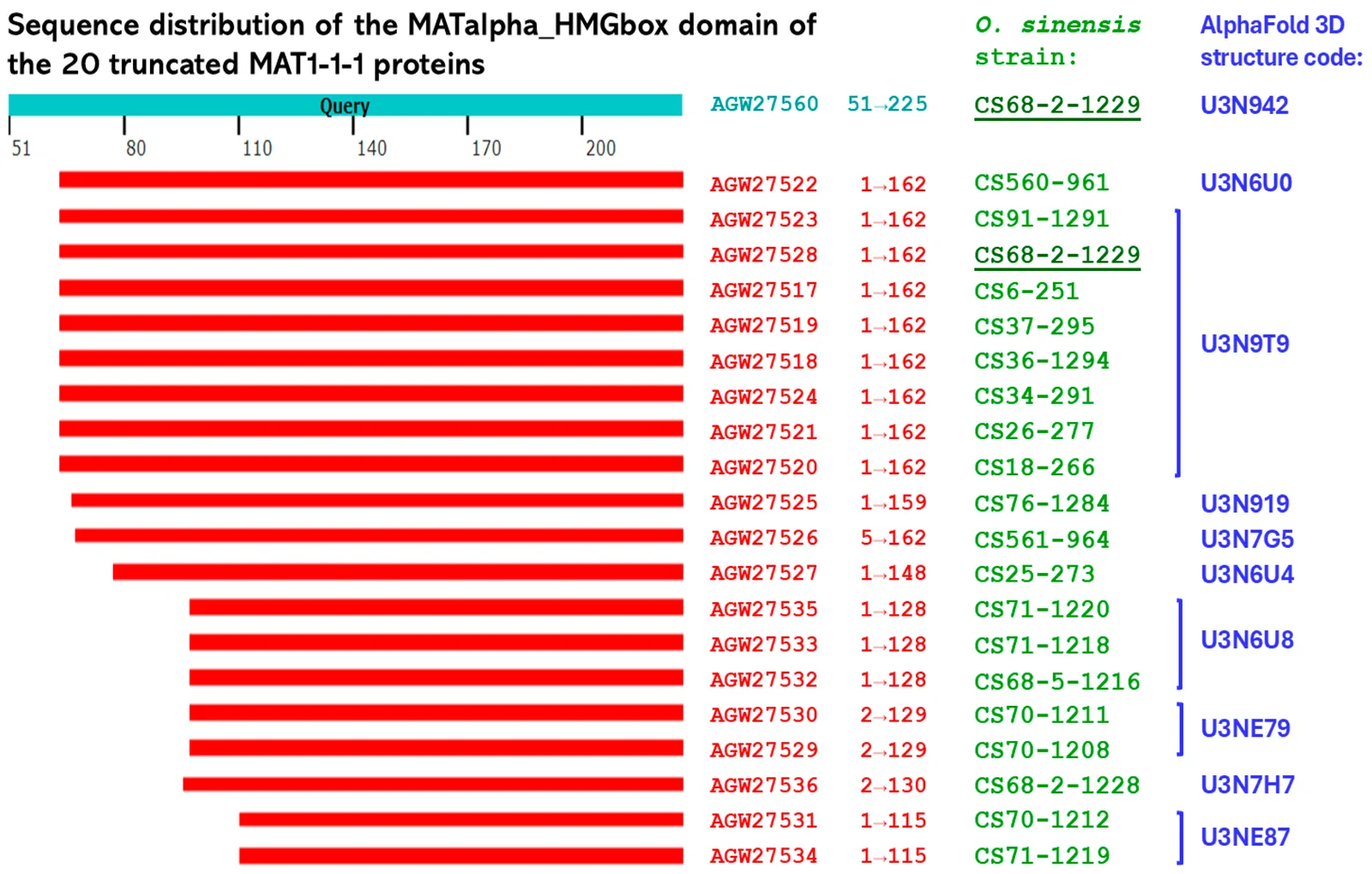
Sequence distribution of the differentially truncated MATα_HMGbox domains at the N-termini among the 20 MAT1-1-1 proteins derived from various *O. sinensis* strains (Table 1; Figure S1). The sequences are aligned relative to the full-length MATα_HMGbox domain (residues 51→225) of the query MAT1-1-1 protein AGW27560 derived from the *H. sinensis* strain CS68-2-1229.

These truncated MAT1-1-1 proteins were encoded by cDNAs amplified from total RNA extracted from 20 *O. sinensis* strains by using the same primer pair, *m1F3*/*m1R3* [14,70]. Relative to the full-length reference protein AGW27560 (AlphaFold model U3N942), these protein variants exhibit truncations of 63–110 residues at the N-termini and 8–18 residues at the C-termini and correspond to 9 distinct AlphaFold-predicted 3D structural morphotypes (U3N9T9, U3N6U0, U3N919, U3N7G5, U3N6U4, U3N6U8, U3NE79, U3N7H7, and U3NE87) (Figure S1; Table 1). In the reference authentic MAT1-1-1 protein AGW27560, the MATα_HMGbox domain spans amino acids 51→225 (highlighted in pink in Figure S1). All 20 variant MAT1-1-1 proteins display variable N-terminal truncations within their MATα_HMGbox domains, ranging from 13–60 amino acid residues (Figure 1). In addition, 1–4 amino acid substitutions were identified in several truncated protein variants and are highlighted in green in Figure S1.

### 2.2. Primary Structures of the MAT1-2-1 Proteins in O. sinensis Strains

A sequence alignment of 20 MAT1-2-1 proteins (AGW27537–AGW27556 [14]) produced by these 20 *O. sinensis* strains and the representative authentic MAT1-2-1 protein AEH27625 (AlphaFold model D7F2E9) derived from the *H. sinensis* strain CS2 is shown in Figure S2 [70]. Among these 20 MAT1-2-1 proteins, 15 (AGW27537–AGW27542, AGW27544–AGW27547, AGW27549–AGW27551, AGW27553, and AGW27556) are full-length proteins. The remaining 5 proteins (AGW27543, AGW27548, AGW27552, AGW27554, and AGW27555) exhibit C-terminal truncations of 64–75 amino acids. These truncated proteins were encoded by *MAT1-2-1* cDNAs amplified from total RNA extracted from the *O. sinensis* strains by using the same primer pair, *Mat1-2F*/*Mat1-2R* [14,70]. In the full-length reference protein AEH27625 [70], the HMG-box_ROX1-like domain spans amino acid residues 127→197, highlighted in pink in Figure S2. In addition, 1–2 amino acid substitutions were identified in many of the proteins and are marked in green in Figure S2.

Among the 20 MAT1-2-1 proteins, 3 full-length proteins (AGW27538, AGW27539, and AGW27541) were 100% identical to the reference authentic MAT1-2-1 protein AEH27625 and shared the same tertiary structure as the AlphaFold model D7F2E9 (Table 1; Figure S2). The remaining 17 MAT1-2-1 proteins contained tyrosine^144^→histidine^144^ (Y^144^→H^144^) substitutions within the HMG-box_ROX1-like domains, leading to a shift in the hydropathy index from −1.3 to −3.2 (Table S3) [73].

The truncated MAT1-2-1 protein AGW27554 contained an additional glutamine^185^→threonine^185^ (Q^185^→T^185^) substitution within its HMG-box_ROX1-like domain, corresponding to a hydropathy index shift from −3.5 to −0.7 (Table S3; Figure S2) [73].

**Figure 2:**
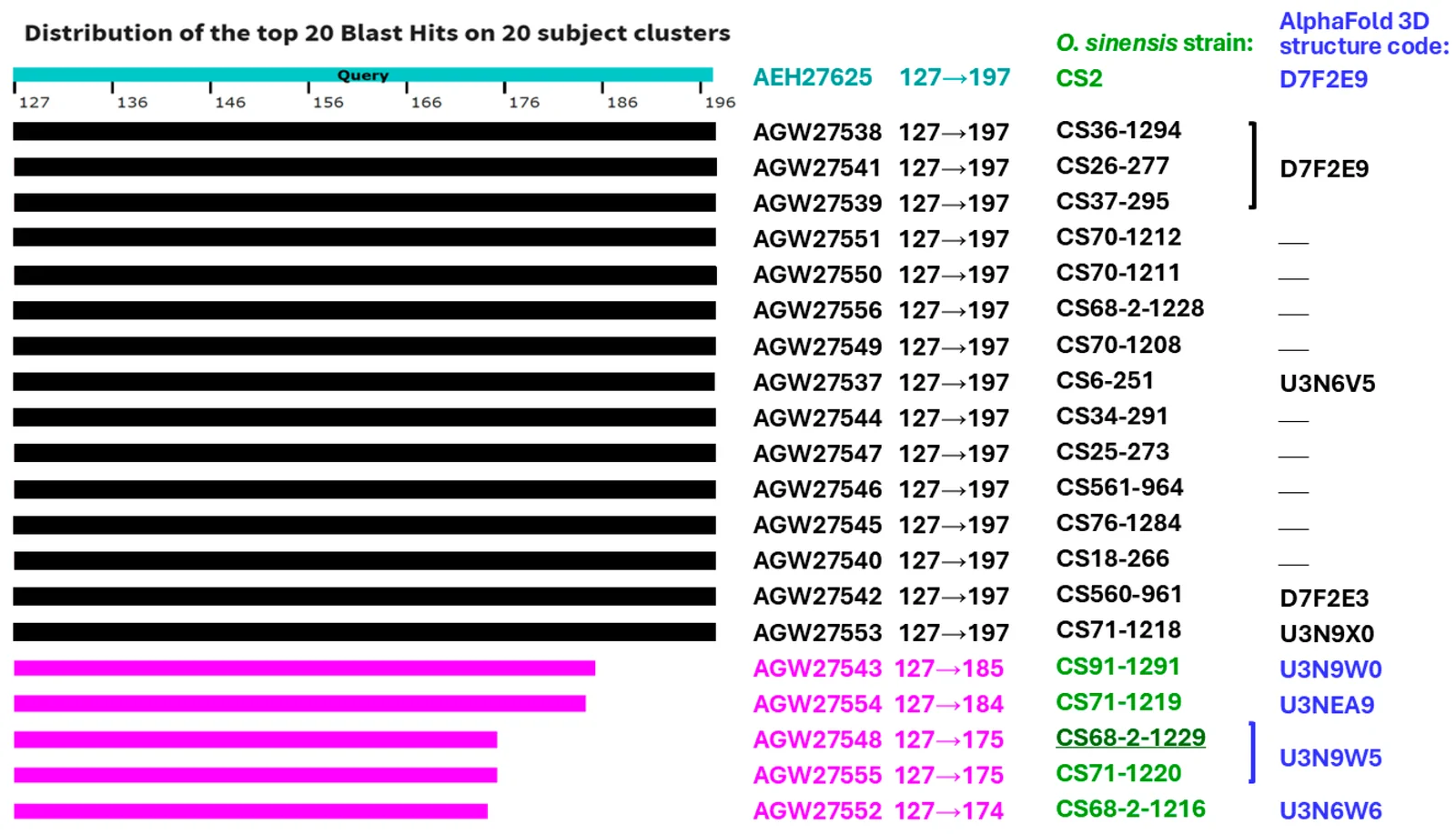
Sequence distribution of the HMG-box_ROX1-like domains of 15 full-length and 5 C-terminally truncated MAT1-2-1 proteins derived from 20 *O. sinensis* strains (Table 1; Figure S2). The sequences are aligned relative to the HMG-box_ROX1-like domain (residues 127→197) of the full-length MAT1-2-1 protein AEH27625 derived from the *H. sinensis* strain CS2.

Two full-length proteins, AGW27537 and AGW27553, exhibited additional amino acid substitutions. Specifically, AGW27537 carried a valine^78^→isoleucine^78^ (V^78^→I^78^) substitution upstream of the HMG-box_ROX1-like domain, corresponding to a hydropathy index shift from 4.2 to 4.5 (Table S3) [73]), whereas AGW27553 contained a lysine^174^→residue^174^ unidentified (K^174^→X^174^) substitution within the HMG-box_ROX1-like domain (Figure 2).

Eleven of the 20 MAT1-2-1 proteins exhibited diverse conformations corresponding to 7 distinct AlphaFold-predicted 3D structural morphotypes (Table 1). The remaining 9 proteins do not have predicted 3D structures available in the AlphaFold database.

In the five C-terminally truncated MAT1-2-1 proteins, the truncated regions (ranging from 12–23 residues) are located within the HMG-box_ROX1-like domains (Figure 2 and Figure S2). All amino acid substitutions, except for the valine^78^→isoleucine^78^ (V^78^→I^78^) substitution, are within the HMG-box_ROX1-like domains. The truncations and amino acid substitutions are associated with differences in hydrophobicity and the predicted secondary/tertiary structure of the HMG-box_ROX1-like domains, corresponding to 4 distinct AlphaFold 3D structural morphotypes (Table 1; Figure 2 and Figure S2), highlighting the structural diversity of this protein family across *O. sinensis* strains.

### 2.3. Differential Natural Pairings of MAT1-1-1 and MAT1-2-1 Proteins Simultaneously Produced by O. sinensis Strains

Li et al. [36] deposited internal transcribed spacer (ITS) sequences for 15 of the 20 *O. sinensis* strains in GenBank for phylogenetic positioning. Among these 15 strains, only Group-A (GC-biased Genotype #1 *H. sinensis*) ITS sequences were reported for 10 strains (highlighted in brown in Table 1). ITS sequences corresponding to both GC-biased Genotype #1 and AT-biased Genotype #17 (or #5) were reported for the remaining 5 strains (highlighted in red in Table 1) (Tables S1 and S2). The authors proposed that the AT-biased sequences represented nonfunctional pseudogenic repetitive copies within the genome of GC-biased Genotype #1 *H. sinensis* and concluded that all the strains represented pure *O. sinensis* cultures. ITS sequences of the remaining 5 strains (CS26-277, CS36-1294, CS68-5-1216, CS70-1211, and CS70-1212) are unavailable in GenBank (highlighted in black in Table 1).

With respect to the MAT1-1-1 and MAT1-2-1 proteins analyzed here, the assumption of homogeneity for these *O. sinensis* strains warrants careful interpretation for several reasons. (1) ITS sequences of AT-biased *O. sinensis* Genotypes #4–6 and #15–17 are absent in the genome of GC-biased Genotype #1; instead, they are apparently genome-independent fungal taxa [9,10,13,35,38]. (2) Li et al. [36] and collaborators [14] reported multicellular heterokaryotic microstructures in *C. sinensis* ascospores and hyphae that contain multiple mono-, bi-, tri-, and tetra-nucleate cells. Zhang & Zhang [72] discussed the possibility that such polynucleate cells could harbor heterogeneous hereditary substances. (3) Culture-dependent ITS profiling may not fully capture nonculturable fungal taxa and low-abundance amplicons, particularly when different primer sets and denaturation temperatures differentially affect amplification efficiency among genotypes (*cf*. Section 3.2) [9,10,12,13,37,74]. (4) Culture-independent studies have indicated that *C. sinensis* ascospores contain psychrophilic GC-biased Genotypes #1 and #13–14 and AT-biased Genotypes #5–6 and #16 of *O. sinensis*, which may share indistinguishable morphology and in vitro culture characteristics, along with additional mesophilic fungal taxa. Moreover, the stromata and caterpillar bodies of insect–fungal complexes contain additional *O. sinensis* genotypes and heterospecific fungi [9,10,11,12,13,14,15,16,20,21,31,32,34,37,40,41].

**Figure 3:**
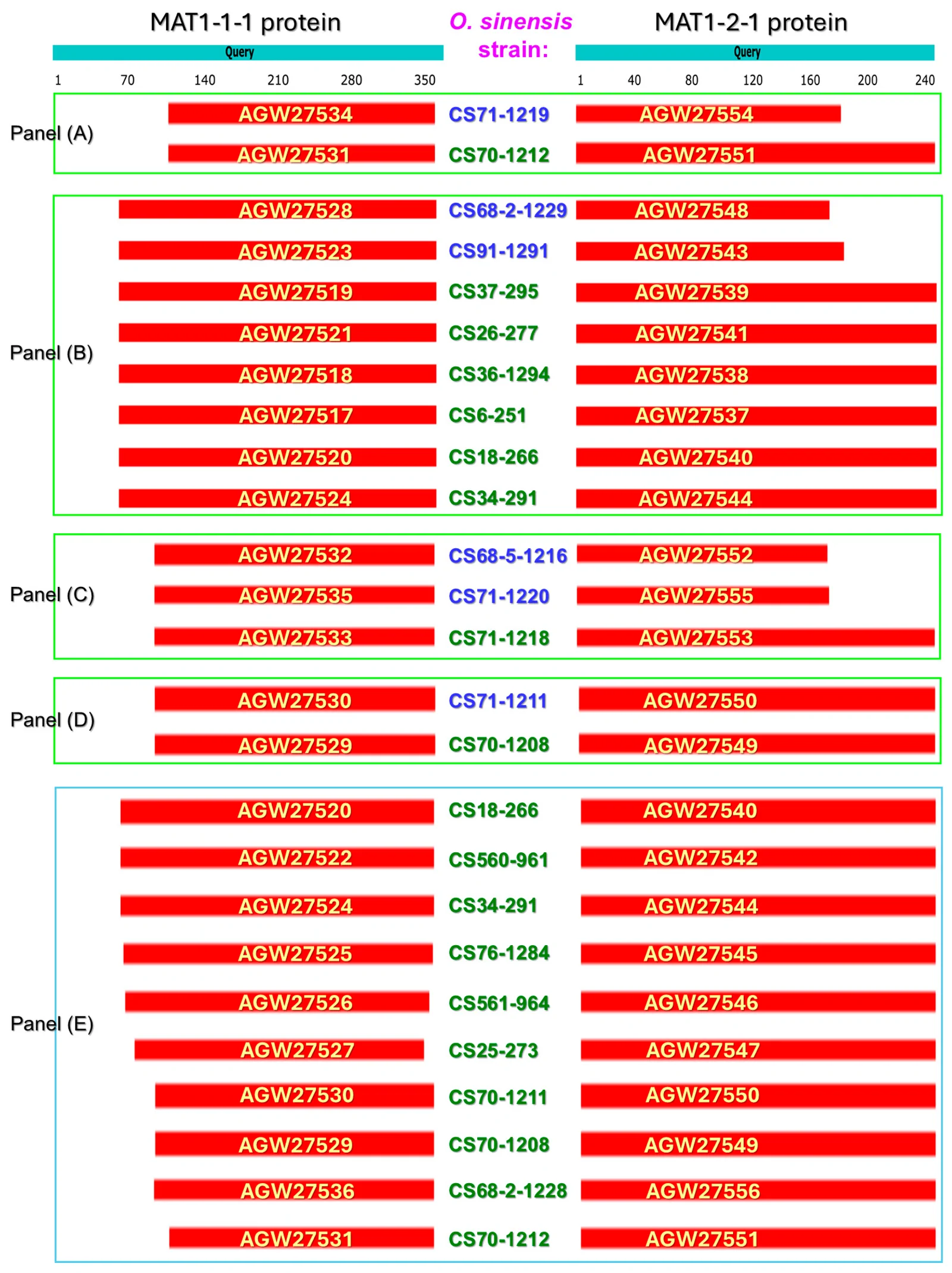
Sequence distributions of naturally paired MAT1-1-1 and MAT1-2-1 proteins simultaneously produced by *O. sinensis* strains. Group-I strains are shown in blue, whereas Group-II strains are shown in green (Table 2). The MAT1-1-1 protein AGW27560 and the MAT1-2-1 protein AEH27625, derived from the *H. sinensis* strains CS68-2-1229 and CS2, were used as the reference query sequences for the MAT1-1-1 and MAT1-2-1 proteins, respectively. Panels outlined with open green or blue rectangles denote proteins assigned to identical AlphaFold 3D structural models for MAT1-1-1 or MAT1-2-1 proteins, respectively.

The 20 *O. sinensis* strains can be categorized into 2 groups according to the observed natural pairing patterns of the 2 mating proteins (Table 2). Group I comprises 5 strains that simultaneously produce paired truncated MAT1-1-1 and MAT1-2-1 proteins. Group II comprises the remaining 15 *O. sinensis* strains that simultaneously produce truncated MAT1-1-1 proteins paired with full-length MAT1-2-1 proteins. The differential N- and/or C-terminal truncations and the diverse amino acid substitutions within the mating proteins are summarized in Table 2, with proteins sharing identical AlphaFold 3D structural morphotypes denoted by the same suffix symbol (*, ⁋, †, ⁑, ‡, or ♦). The sequence distributions of the differentially naturally paired but structurally divergent MAT1-1-1 and MAT1-2-1 proteins coexpressed in each of the 20 *O. sinensis* strains are presented in Figure 3. Across strains, these naturally paired mating proteins exhibit variable N- and/or C-terminal truncations and diverse amino acid substitutions (Figure 1, Figure 2, Figure 3, Figures S1 and S2; Table 2).

### 2.4. O. sinensis Strains Simultaneously Produce MAT1-1-1 Proteins with Identical Tertiary Structures and MAT1-2-1 Proteins with Various Tertiary Structures

#### 2.4.1. MAT1-1-1 Proteins Sharing the Same AlphaFold-Predicted 3D Structure as the AlphaFold Model U3NE87 Are Naturally Paired with Structurally Diverse MAT1-2-1 Proteins

**Figure 4:**
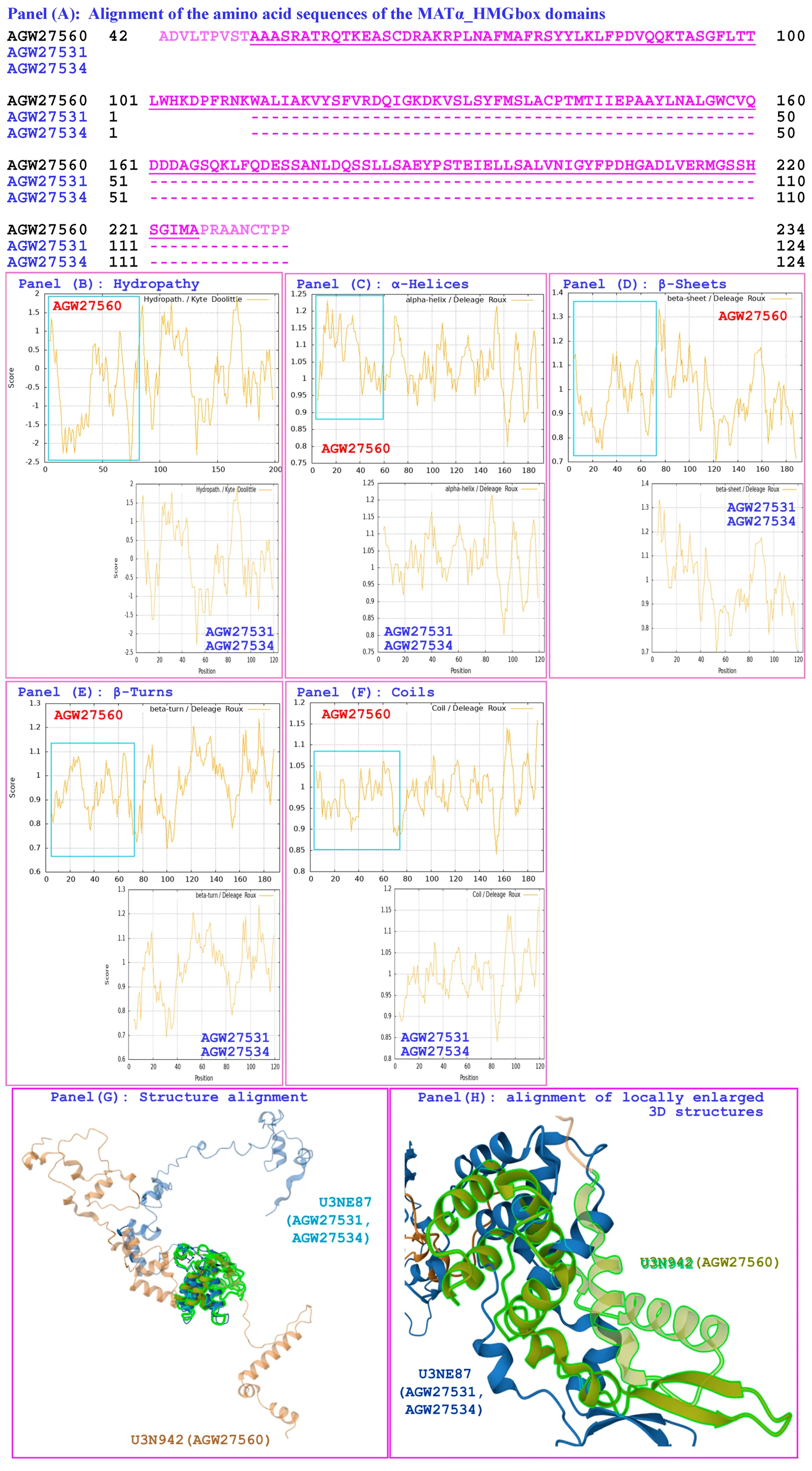
Correlations among changes in hydrophobicity and the primary, secondary, and tertiary structures of the MATα_HMGbox domains of the MAT1-1-1 proteins. The reference protein AGW27560 (AlphaFold model U3N942) originated from the *H. sinensis* strain CS68-2-1229, whereas the variant proteins AGW27531 and AGW27534 (AlphaFold model U3NE87) were derived from the *O. sinensis* strains CS70-1212 and CS71-1219, respectively. Panel (**A**) displays sequence alignments of the MATα_HMGbox domains (underlined segment) of the MAT1-1-1 proteins; hyphens indicate identical amino acid residues, and blank spaces denote unmatched sequence alignment gaps. Panels (**B**–**F**) show ExPASy ProtScale plots illustrating changes in hydrophobicity and predicted secondary structural characteristics of the proteins are in for hydropathy, α-helices, β-sheets, β-turns, and coils, respectively; while the blue rectangles in the ExPASy plots highlight the N-terminally truncated region. Panel (**G**) shows pairwise structural superposition of AlphaFold-predicted 3D structures performed using the Template-Model align (TM-align) algorithm to compare the overall folds of the 2 tertiary structural models (U3N942 vs. U3NE87). The 3 critical α-helices within the DNA-binding domain of the reference protein AGW27560 are highlighted with light green outlines. Panel (**H**) shows independent structural superpositions of the MATα_HMGbox domains using the Smith-Waterman 3D (SW 3D) algorithm to optimize local structural alignment and enable clear visualization of regional structural differences, focusing on the 3 α-helices that form the hydrophobic core.

Panel (A) of Figure 3 presents the Group-I *O. sinensis* strain CS70-1219, which is reported to contain only Group-A ITS sequences, and the Group-II strain CS70-1212, which lacks an ITS record in GenBank (Table 1 and Table 2). These 2 strains produced the MAT1-1-1 proteins AGW27534 and AGW27531, respectively, each carrying 110- and 8-residue truncations at the N- and C-termini, respectively (Figure S1; Table 2). The hydrophobic properties and structural features of the MATα_HMGbox domains of these truncated MAT1-1-1 proteins, assigned to the same AlphaFold-predicted 3D structural morphotype U3NE87 (designated by the suffix symbol “*” in Table 1 and Table 2), were compared with those of the reference protein AGW27560 (AlphaFold model U3N942), as displayed in Figure 4.

Panel (A) of Figure 4 shows that the MATα_HMGbox domains of the proteins AGW27534 and AGW27531 each contain 60-residue truncations at the N-terminus. These truncations within the DNA-binding domains are associated with altered hydrophobicity and predicted secondary structural features, as reflected by changes in the topological profiles and waveform patterns of the ExPASy ProtScale plots for hydropathy, α-helices, β-sheets, β-turns, and coils (Panels (B–F) of Figure 4, respectively); while the blue rectangles in the plots highlight the N-terminally truncated region. A Template-Model align (TM-align) structural superposition algorithm was used to compare overall folding [75,76]. Panel (G) shows completely separated overall stereostructures of UniProt codes U3N942 (reference protein AGW27560) and U3NE87 (variant proteins AGW27531 and AGW27534). Panel (H) shows the further maximization of the local structure similarity/dissimilarity performed using the Smith–Waterman 3D (SW 3D) algorithm and reveals non-overlaid structures of the locally magnified 3 α-helix regions within the MATα_HMGbox domains of the 2 AlphaFold models (U3N942 vs. U3NE87) [75,77].

In addition, the Group-I *O. sinensis* strain CS71-1219 simultaneously coproduces the MAT1-2-1 protein AGW27554 [14], which contains a 65-residue truncation at the C-terminus (AlphaFold model U3NEA9), compared with the reference MAT1-2-1 protein AEH27625 (AlphaFold model D7F2E9) derived from the *H. sinensis* strain CS2 [70] (Figure 2 and Figure S2; Table 1 and Table 2).

**Figure 5:**
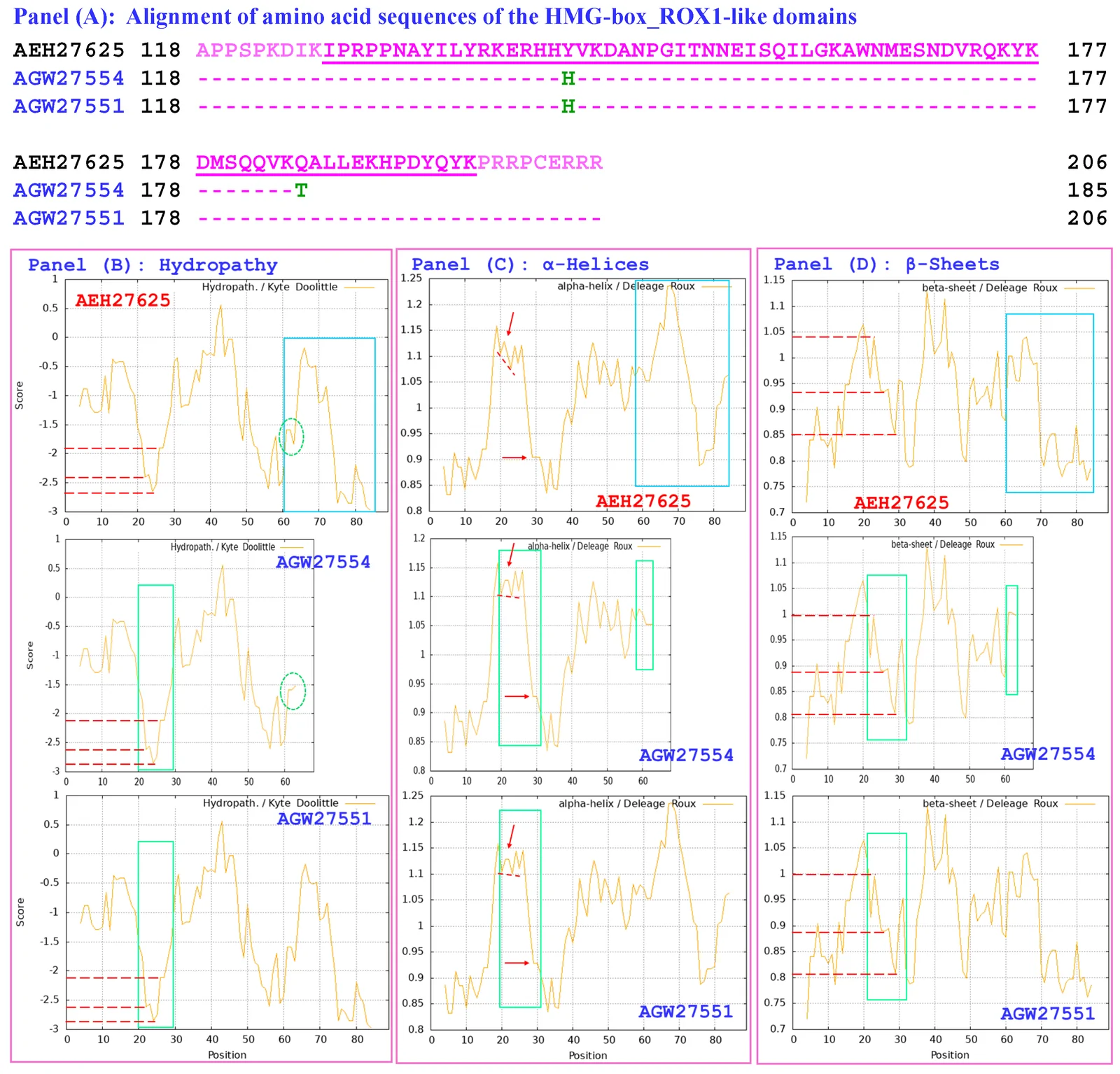

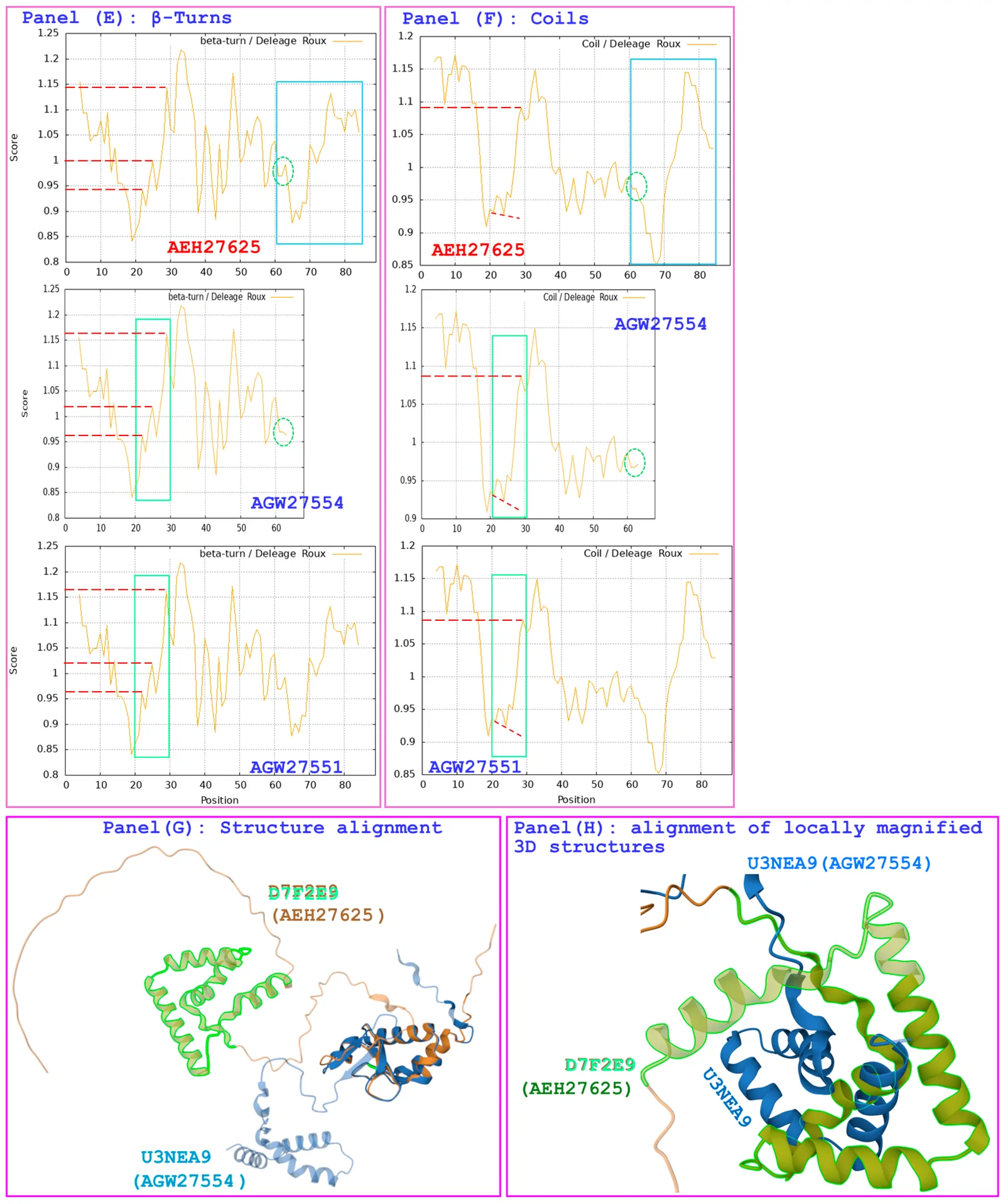
Correlations among changes in hydrophobicity and the primary, secondary, and tertiary structures of the HMG-box_ROX1-like domains of MAT1-2-1 proteins. The reference protein AEH27625 (AlphaFold model D7F2E9) was derived from the *H. sinensis* strain CS2, whereas the variant MAT1-2-1 proteins AGW27554 (AlphaFold model U3NEA9) and AGW27551 (no AlphaFold 3D model assigned) were derived from the *O. sinensis* strains CS71-1219 and CS70-1212, respectively. Panel (**A**) shows alignments of the amino acid sequences of the HMG-box_ROX1-like domains (underlined segment) in MAT1-2-1 proteins. Amino acid substitutions are highlighted in green, hyphens indicate identical amino acid residues, and blank spaces denote unmatched sequence gaps. ExPASy ProtScale analyses are shown in Panels (**B**–**F**), illustrating changes in hydrophobicity and predicted secondary structural characteristics, including hydropathy, α-helices, β-sheets, β-turns, and coils. The open blue rectangles highlight the C-terminal truncation regions in the ExPASy plots, whereas the open green rectangles and circles with red dashed auxiliary lines or directional arrows highlight changes in the topological configuration and waveform patterns due to amino acid substitutions. Panel (**G**) shows pairwise structural superposition of AlphaFold models (D7F2E9 vs. U3NEA9) using the TM-align algorithm to compare the overall folds of the 2 tertiary structures. The 3 core α-helix regions within the DNA-binding domain of the reference protein AEH27625 are highlighted with light green outlines. Panel (**H**) shows independent structural superpositions of the HMG-box_ROX1-like domains using the SW 3D algorithm to optimize local structural alignment and enable clear visualization of regional structural differences, focusing on the 3 α-helices that form the hydrophobic core.

Panel (A) of Figure 5 shows that AGW27554 contains a 12-residue C-terminal truncation within the HMG-box_ROX1-like domain. This protein also has 2 amino acid substitutions: tyrosine^144^→histidine^144^ (Y^144^→H^144^; hydropathy index shift from −1.3 to −3.2; Table 2 and Table S3) and glutamine^185^→threonine^185^ (Q^185^→T^185^; hydropathy index shift from −3.5 to −0.7) [73]. On the other hand, the Group-II strain CS70-1212 simultaneously coproduces the full-length MAT1-2-1 protein AGW27551, which carries a single tyrosine^144^→histidine^144^ (Y^144^→H^144^) substitution within the HMG-box_ROX1-like domain (hydropathy index shift from −1.3 to −3.2 [73]) (Figure S2; Table 2 and Table S3). Consistent with these sequence alterations, Panels (B–F) of Figure 5 demonstrate corresponding differences in the topological profiles and waveform patterns of the ExPASy ProtScale plots for hydropathy, α-helices, β-sheets, β-turns, and coils, respectively; while the open green rectangles and circles with red dashed auxiliary lines or directional arrows highlight these changes caused by amino acid substitutions. Panel (G) of Figure 5 presents the TM-align structural superposition of the MAT1-2-1 proteins corresponding to UniProt codes U3N942 (reference protein AGW27560) and U3NE87 (variant protein AGW27554) [75,76]. The 2 AlphaFold models are distinctly folded with complete separation of the spatial structures of the 3 α-helix regions within the MATα_HMGbox domains. Further maximizing the local structural similarity/dissimilarity by using the SW 3D algorithm, Panel (H) demonstrates non-overlaid structures for the locally magnified 3 α-helix regions of the 2 AlphaFold models (D7F2E9 vs. U3NEA9) [75,77]. Because AGW27551 was not assigned an AlphaFold 3D model, it was excluded from the 3D structural alignment analysis.

#### 2.4.2. MAT1-1-1 Proteins Sharing the Same Tertiary Structure as the AlphaFold Model U3N9T9 Are Naturally Paired with MAT1-2-1 Proteins That Exhibit Diverse 3D Structures

Among the 8 *O. sinensis* strains shown in Panel (B) of Figure 3, 4 strains (CS37-295, CS18-266, CS91-1291, and CS68-2-1229) were reported to contain only Group-A ITS sequences (highlighted in brown in Table 2); 2 strains (CS6-251 and CS34-291) were reported to contain both Group-A and Group-C ITS sequences (highlighted in red), whereas no ITS sequence records were deposited in GenBank for the remaining 2 strains (CS36-1294 and CS26-277; highlighted in black). These strains produce variant MAT1-1-1 proteins (AGW27517–AGW27521, AGW27523, AGW27524, and AGW27528) that each contain 63- and 8-residue truncations at the N- and C-termini, respectively, and share the same AlphaFold-predicted 3D structural morphotype U3N9T9 (designated by the suffix symbol “⁋” in Table 2). The reference MAT1-1-1 protein AGW27560 (AlphaFold model U3N942) originates from the strain CS68-2-1229 [14].

Panel (A) of Figure S3 shows that the MATα_HMGbox domains of all 8 MAT1-1-1 proteins contain a common 13-residue N-terminal truncation. Compared with the reference protein AGW27560 (AlphaFold model U3N942), this truncation is associated with changes in the topological profiles and waveform patterns of the ExPASy ProtScale plots for hydropathy, α-helices, β-sheets, β-turns, and coils (Panels (B–F) of Figure S3, respectively); while the open blue rectangles in the plots highlight the N-terminally truncated region. Panel (G) of Figure S3 presents the TM-align structural superposition of the tertiary structures of UniProt models U3N942 and U3N9T9 to compare the overall folds of the 2 protein structures [75,76]. The 3 α-helix region of the MATα_HMGbox domain of the reference model U3N942 are highlighted with light green outlines and not overlaid with that of the subject protein model U3N9T9. The local similarity of the 3 α-helix regions of the 2 AlphaFold models was further maximized using the SW 3D algorithm, and the non-overlaid structures of the locally magnified 3 α-helices are illustrated in Panel (H) [75,77].

These 8 strains also simultaneously coproduced paired MAT1-2-1 proteins corresponding to distinct 3D structural morphotypes. The 2 Group-I strains (CS68-2-1229 and CS91-1291; highlighted in blue in Figure 3) coproduced the MAT1-2-1 proteins AGW27548 and AGW27543, which carry 74- and 64-residue C-terminal truncations, respectively (Table 2; Figure S2). Each of the proteins harbors a tyrosine^144^→histidine^144^ (Y^144^→H^144^) substitution within the HMG-box_ROX1-like domains, corresponding to a hydropathy index shift from −1.3 to −3.2; Table 2 and Table S3; Figures S2 and S4) [73]. Consistent with these sequence alterations, Panels (B–F) of Figure S4 demonstrate corresponding differences in the topological profiles and waveform patterns of the ExPASy ProtScale plots for hydropathy, α-helices, β-sheets, β-turns, and coils, respectively; while the open green rectangles with red dashed auxiliary lines and directional arrows highlight these changes caused by amino acid substitutions. Panel (G) of Figure S4 presents the TM-align structural superposition of the MAT1-2-1 proteins corresponding to UniProt model D7F2E9 (reference protein AEH27625) vs. U3N9W5 and U3N9W0 (variant proteins AGW27548 and AGW27543, respectively) [75,76]. The 3 AlphaFold models are distinctly folded, with complete separation of the spatial structures of the 3 α-helix regions within the MATα_HMGbox domains. Further maximizing the local structural similarity/dissimilarity by using the SW 3D algorithm, Panel (H) demonstrates non-overlaid structures for the locally magnified 3 α-helix regions of the 3 AlphaFold models (D7F2E9 vs. U3N9W5/U3N9W0) [75,77].

The remaining 6 *O. sinensis* strains in Panel (B) of Figure 3 belong to Group II (highlighted in green in Table 2). Two strains (CS37-295 and CS18-266) contain only Group-A ITS sequences deposited in GenBank (shown in brown in Table 2), 2 strains (CS6-251 and CS34-291) contain both Group-A and Group-C ITS sequences (shown in red), and 2 strains (CS26-277 and CS36-1294) lack deposited ITS records (shown in black). Although all 6 strains produce truncated MAT1-1-1 proteins with the same predicted 3D structural morphotype (AlphaFold code U3N9T9, marked with the suffix symbol “⁋” in Table 2), they simultaneously produce full-length MAT1-2-1 proteins that either correspond to different AlphaFold 3D structural morphs or lack AlphaFold 3D structural models. The 6 full-length MAT1-2-1 proteins can be categorized into 3 subgroups:

1. The coproduced full-length MAT1-2-1 proteins AGW27539, AGW27541, and AGW27538 share 100% sequence identity with the reference MAT1-2-1 protein AEH27625 (Figure S2). These proteins were generated by the strain CS37-295 (with Group-A ITS sequences) and by the strains CS26-277 and CS36-1294 (no ITS records in GenBank) (Table 1 and Table 2; Panel (B) of Figure 3). The 3D structures are identical and correspond to the AlphaFold code **D7F2E9** (suffix symbol “♦” in Table 2).
2. The full-length MAT1-2-1 protein AGW27537 was coproduced by strain CS6-251 (reported to contain both Group-A and Group-C ITS sequences; Table 1 and Table 2; Panel (B) of Figure 3). This protein has 2 amino acid substitutions: a valine^78^→isoleucine^78^ (V^78^→I^78^) substitution upstream of the HMG-box_ROX1-like domain (hydropathy index shift from 4.2 to 4.5; Table S3) and a tyrosine^144^→histidine^144^ (Y^144^→H^144^) substitution (hydropathy index changed from −1.3 to −3.2) within the DNA-binding domain (Figure S2; Table 2 and Table S3; *cf*. Figure 12 of Ref. [65]) [73]. In addition to the detailed comparison of the full-length protein variant AGW27537 (AlphaFold code U3N6V5) and the reference protein AEH27625 (AlphaFold code D7F2E9) previously reported (*cf*. Figure 12 of Ref. [65]), TM-align structural superposition analysis revealed distinct overall folding of the 2 proteins (Panel (A) of Figure S5). SW 3D structural superposition analysis revealed incomplete overlay of the locally magnified 3 α-helix regions within the MATα_HMGbox domains of the proteins (Panel (B) of Figure S5).
3. The full-length MAT1-2-1 proteins AGW27540 and AGW27544 were coproduced by strains CS18-266 (with deposited Group-A ITS sequences) and CS34-291 (containing both Group-A and Group-C ITS sequences), respectively (Table 1 and Table 2; Panel (B) of Figure 3). Each of them harbors a tyrosine^144^→histidine^144^ (Y^144^→H^144^) substitution within its HMG-box_ROX1-like domain (hydropathy index shift from −1.3 to −3.2; Table S3). They share 99.6% sequence similarity with the authentic MAT1-2-1 protein AEH27625 (Figure S2). No AlphaFold-predicted 3D structural models were assigned to these 2 proteins.

#### 2.4.3. MAT1-1-1 Proteins with the Same Tertiary Structure as the AlphaFold 3D Structural Code U3N6U8 Naturally Paired with MAT1-2-1 Proteins with Different Tertiary Structures

Panel (C) of Figure 3 presents 3 *O. sinensis* strains: the Group-I strains CS71-1220 and CS68-5-1216 and the Group-II strain CS71-1218. According to the reported ITS records [14,36] (Table 1), only Group-A ITS sequences have been deposited in GenBank for strain CS71-1218; for strain CS71-1220, both Group-A and Group-C ITS sequences are present, while there are no deposited ITS records in GenBank for strain CS68-5-1216 (Table 1). These strains produced the MAT1-1-1 proteins AGW27535, AGW27532, and AGW27533 (Figure 3 and Figure S1), each carrying 97- and 8-residue truncations at the N- and C-termini, respectively, and sharing the same predicted 3D structural morphotype (AlphaFold code U3N6U8; suffix symbol “†” in Table 2).

Panel (A) of Figure S6 shows that the MATα_HMGbox domains of the MAT1-1-1 proteins AGW27535, AGW27532, and AGW27533 (AlphaFold model U3N6U8) contain a 47-residue N-terminal truncation relative to the reference protein AGW27560 (AlphaFold model U3N942). This truncation is associated with changes in the topological profiles and waveform patterns of the ExPASy ProtScale plots for hydropathy, α-helices, β-sheets, β-turns, and coils (Panels (B–F) of Figure S6, respectively); the open blue rectangles in the plots highlight the N-terminally truncated region. Panel (G) presents the TM-align structural superposition of the MAT1-1-1 proteins corresponding to UniProt models U3N942 and U3N6U8 [75,76], showing distinct conformations of the 2 AlphaFold 3D structural models. The local structural dissimilarity was further maximized using the SW 3D algorithm; Panel (H) shows the incomplete overlaid structures for the locally magnified 3 α-helix regions of the 2 AlphaFold monotypes (U3N942 vs. U3N6U8) [75,77].

The 2 Group-I strains, CS68-5-1216 and CS71-1220, also produced the MAT1-2-1 proteins AGW27552 and AGW27555 (AlphaFold models U3N6W6 and U3N9W5), which carry 75- and 74-residue C-terminal truncations, respectively (Table 2; Figure S2). In contrast, the Group-II strain CS71-1218 produced the full-length MAT1-2-1 protein AGW27553 (AlphaFold model U3N9X0).

Panel (A) of Figure S7 shows that AGW27552 and AGW27555 contain 23- and 22-residue truncations, respectively, at the C-termini of their HMG-box_ROX1-like domains. All 3 MAT1-2-1 proteins contain tyrosine^144^→histidine^144^ (Y^144^→H^144^) substitutions within the HMG-box_ROX1-like domain, corresponding to a hydropathy index shift from −1.3 to −3.2; Table S3) [73]. AGW27553 contains an extra lysine^175^→ residue^175^ unidentified (K^175^→X^175^) substitution within the DNA-binding domain (Figures S2 and S7). For AGW27552 and AGW27555, the truncations and amino acid substitutions are associated with corresponding differences in hydrophobicity and secondary structural features within the HMG-box_ROX1-like domains, as reflected by altered topological profiles and waveform patterns of the ExPASy ProtScale plots for hydropathy, α-helices, β-sheets, β-turns, and coils (Panels (B–F) of Figure S7, respectively), while the open green rectangles with red dashed auxiliary lines or directional arrows highlight these changes caused by amino acid substitutions. Panel (G) of Figure S7 presents TM-align structure superposition of the MAT1-2-1 proteins under UniProt codes D7F2E9 (reference protein AEH27625) vs. models U3N6W6 and U3N9W5 (variant proteins AGW27552 and AGW27555, respectively) [75,76]. The 3 AlphaFold models are distinctly folded with completely separation of spatial structures of the 3 α-helix regions within the MATα_HMGbox domains, with the 3 α-helix regions of the reference protein AEH27625 are highlighted with light green outlines. Further maximizing the local structural dissimilarity using the SW 3D algorithm, Panel (H) demonstrates incompletely overlayed patterns for the locally magnified 3 α-helix regions of the 3 AlphaFold models (D7F2E9 vs. U3N6W6/U3N9W5) [75,77].

The strain CS71-1218 coproduced the truncated MAT1-1-1 AGW27533 (AlphaFold model U3N6U8; Figure S6) and the full-length MAT1-2-1 protein AGW27553 (AlphaFold model U3N9X0). However, because of existence of an unknown amino acid substitution (K^175^→X^175^) in the protein AGW27553, it was excluded from structural overlay analysis.

The strains CS71-1220 and CS68-2-1229 produced the MAT1-2-1 proteins AGW27555 and AGW27548, respectively, which share the identical structures (AlphaFold model U3N9W5; suffix symbol “⁑” in Table 2) and analyzed in Figure S7. These 2 strains also produced MAT1-1-1 proteins AGW27535 and AGW27528 with distinct structures (AlphaFold models U3N6U8 and U3N9T9; suffix symbols “†” and “⁋” in Table 2), which are further discussed in Section 2.5.1 below.

#### 2.4.4. MAT1-1-1 Proteins with the Same 3D Structure as the AlphaFold Structural Model U3NE79 Naturally Paired with MAT1-2-1 Proteins with Different Tertiary Structures

Panel (D) of Figure 3 presents 2 Group-II strains, CS70-1211 and CS70-1208 (Table 1 and Table 2). CS70-1208 contains both Group-A and Group-C ITS sequences, whereas CS70-1211 lacks a deposited ITS record in GenBank. These strains produced the MAT1-1-1 proteins AGW27530 and AGW27529, each of which carried 97- and 8-residue truncations at the N- and C-termini, respectively (Figure S1; Table 2), and shared the same AlphaFold structural morph, U3NE79 (suffix symbol “‡” in Table 1 and Table 2).

Panel (A) of Figure S8 shows that the MATα_HMGbox domains of AGW27530 and AGW27529 contain a 46-residue truncation at the N-terminus and have a phenylalanine^97^→valine^1^ (F^97^→V^1^) substitution (Figure S1), corresponding to a hydropathy index shift from 2.8 to 4.2; Table S3), indicating increased hydrophobicity [73]. Panels (B–F) of Figure S8 show associated alterations in secondary structural features, as reflected by changes in the topological profiles and waveform patterns of the ExPASy ProtScale plots for hydropathy, α-helices, β-sheets, β-turns, and coils, respectively. Panel (G) of Figure S8 presents the TM-align structural superposition of the tertiary structures of UniProt models U3N942 and U3NE79 and shows distinct overall folds of the 2 tertiary structural models [75,76]. The local dissimilarity of the 3 α-helix regions of the 2 AlphaFold models was further maximized using the SW 3D algorithm, and Panel (H) shows the incomplete overlaid structures of the locally magnified 3 α-helix regions within the MATα_HMGbox domains of the 2 AlphaFold models (U3N942 vs. U3NE79) [75,77].

These 2 *O. sinensis* strains also coproduce the full-length MAT1-2-1 proteins AGW27550 and AGW27549, each of which contain a tyrosine^144^→histidine^144^ (Y^144^→H^144^) substitution within the HMG-box_ROX1-like domain (hydropathy index shift from −1.3 to −3.2), indicating reduced hydrophobicity (Table 2 and Table S3; Figure S2) [73]. AlphaFold-predicted 3D structural models are not assigned to AGW27550 and AGW27549.

### 2.5. O. sinensis Strains Simultaneously Produce MAT1-2-1 Proteins with the Same Tertiary Structure and Truncated MAT1-1-1 Proteins with Different 3D Structures

#### 2.5.1. Truncated MAT1-2-1 Proteins Have Identical Tertiary Structures (AlphaFold Code U3N9W5) Paired with Diverse Truncated MAT1-1-1 Proteins with Different 3D Structures

As shown in Table 2, the Group-I strains CS68-2-1229 (with deposited Group-A ITS sequences) and CS71-1220 (reported to contain both Group-A and Group-C ITS sequences) produced the MAT1-2-1 proteins AGW27548 and AGW27555, respectively, each of which carried a 74-residue truncation at the C-terminus (Figure S2; Panels (B,C) of Figure 3). These proteins share the same predicted 3D structural morph corresponding to the AlphaFold code U3N9W5 (suffix symbol “⁑” in Table 1 and Table 2).

Panel (A) of Figure S4 shows that the HMG-box_ROX1-like domains of AGW27548 and AGW27555 contain a tyrosine^144^→histidine^144^ (Y^144^→H^144^) substitution and a 22-residue C-terminal truncation. The Y^144^→H^144^ substitution corresponds to a hydropathy index shift from −1.3 to −3.2, indicating reduced hydrophobicity (Table S3) [73]. Structural variations are associated with alterations in the secondary structural features surrounding the sites of variation within the HMG-box_ROX1-like domains, which are reflected by changes in the topological profiles and waveform patterns of the ExPASy ProtScale plots for hydropathy, α-helices, β-sheets, β-turns, and coils in Panels (B–F) of Figure S4, respectively. Panel (G) of Figure S4 presents the TM-align structural superposition of the MAT1-2-1 proteins corresponding to UniProt codes D7F2E9 (reference protein AEH27625) vs. U3N9W5 (variant proteins AGW27548 and AGW27555), showing distinct overall folding of the 2 AlphaFold morphs (D7F2E9 vs. U3N9W5) [75,76]. The local structural dissimilarity was further maximized using the SW 3D algorithm, and Panel (H) presents incomplete overlaid structures for the locally magnified 3 α-helix regions of the 3 AlphaFold models [75,77].

These 2 Group-I strains also coproduced the MAT1-1-1 proteins AGW27528 and AGW27535, which carry 63- and 8-residue truncations and 97- and 8-residue truncations at the N- and C-termini, respectively (Figure S1; Table 2). These proteins correspond to distinct 3D structural morphs assigned to AlphaFold models U3N9T9 and U3N6U8 (suffix symbols “⁋” and “†”, respectively, in Table 1 and Table 2) and are separately included in Panels (B,C) of Figure 3, respectively. In addition to the analyses shown in Figures S3 and S6, TM-align structural superposition revealed distinct overall folding of the 3 predicted 3D structural models (U3N942 vs. U3N9T9/U3N6U8) (Panel (A) of Figure S9). SW 3D structural superposition revealed incomplete overlay of the locally magnified 3 α-helix regions within the MATα_HMGbox domains of the proteins (Panel (B) of Figure S9).

#### 2.5.2. Full-Length MAT1-2-1 Proteins with Identical Structures Naturally Paired with Differentially Truncated MAT1-1-1 Proteins That Exhibit Diverse Tertiary Structures

Ten full-length MAT1-2-1 proteins (AGW27540, AGW27542, AGW27544–AGW27547, AGW27549–AGW27551, and AGW27556) share identical primary structures (Table 2; Figure S2). Each protein has a tyrosine^144^→histidine^144^ (Y^144^→H^144^) substitution located within the first of the 3 core α-helices of the HMG-box_ROX1-like domain, corresponding to a hydropathy index shift from −1.3 to −3.2 (Figure S2; Table 2 and Table S3) [73]. These proteins were simultaneously produced by the Group-II strains CS18-266, CS560-961, CS34-291, CS76-1284, CS561-964, CS25-273, CS70-1208, CS70-1211, CS70-1212, and CS68-2-1228 shown in Panel (E) of Figure 3. Among these proteins, only AGW27542 was assigned an AlphaFold 3D structural model (**D7F2E3**) (*cf*. Figure 12 of Ref. [65]), whereas the remaining 9 full-length MAT1-2-1 proteins do not have available AlphaFold structural models.

These 10 strains also simultaneously produced MAT1-1-1 proteins (AGW27520, AGW27522, AGW27524–AGW27527, AGW27529–AGW27531, and AGW27536), which exhibit variable N- and C-terminal truncations and diverse amino acid substitutions (Figure S1; Table 2). The associated structural variations correspond to AlphaFold 3D structural codes (U3N6U0, U3N919, U3N7G5, U3N6U4, U3NE79, U3NE87, and U3N7H7) and exhibit significant overall folding differences, as determined using the TM-align structural superposition algorithm, and subtle variations in the tertiary structure in the locally magnified 3 core α-helix regions of DNA-binding domains, as revealed by the SW 3D structure alignment algorithm (Table 2; Figure 4, Figures S3, S8 and S10). Collectively, these observations indicate that MAT1-2-1 proteins with identical primary structures differentially naturally pair with structurally diverse MAT1-1-1 proteins across *O. sinensis* strains.

### 2.6. Differences Between the Presence/Absence Patterns of Mating-Type Genes in H. sinensis Genome Assemblies and Naturally Paired MAT1-1-1 and MAT1-2-1 Proteins Simultaneously Produced by the Analyzed O. sinensis Strains

Table 3 summarizes the reported co-occurrence and differential occurrence patterns of MAT1-1-1 and MAT1-2-1 proteins across *C. sinensis* insect-fungal complexes, wild-type *C. sinensis* isolates, and *O. sinensis* strains. The MAT1-1-1 and MAT1-2-1 proteins individually occurred in 52.7% and 22.3% of the 184 samples, respectively, but co-occurred in only 25.0% of the samples.

The *MAT1-1-1* and *MAT1-2-1* genes coexist in the genome assemblies ANOV00000000 and LKHE00000000 of the *H. sinensis* strains Co18 and 1229, respectively [64,78]. However, the *MAT1-1-1* gene is absent from genome assemblies LWBQ00000000 and NGJJ00000000 of the *H. sinensis* strains ZJB12195 and CC1406-20395, respectively, whereas the *MAT1-2-1* gene is absent from genome assemblies JAAVMX000000000 and JBISKU000000000 of the *H. sinensis* strains IOZ07 and PAD37595, respectively [65,78,79,80,81,82]. This differential presence/absence pattern observed at the genomic level in *H. sinensis* strains, however, was not replicated at the protein level in the dataset analyzed herein. All 20 *O. sinensis* strains simultaneously produced naturally paired MAT1-1-1 and MAT1-2-1 proteins without omissions (Table 1 and Table 2; Figure 3). Taken together, the contrasts between the genomic and protein-level occurrence patterns suggest the possibility that multiple co-occurring fungi within these impure *O. sinensis* strains contributed to the naturally paired mating proteins, including both authentic and variant forms.

Tyrosine^81^→methionine (Y^81^→M) and threonine^353^→serine (T^353^→S) substitutions were identified within the MAT1-1-1 proteins encoded by the genome assemblies LKHE00000000, JAAVMX000000000 and JBISKU010000031 of the *H. sinensis* strains 1229, IOZ07, and PAD37595, respectively [65,78,81,82]. In addition, serine^158^→alanine (S^158^→A) substitutions were detected within the MAT1-2-1 proteins encoded by the genome assemblies ANOV00000000, LKHE00000000, LWBQ00000000, and NGJJ00000000 of the *H. sinensis* strains Co18, 1229, ZJB12195, and CC16-20395, respectively [64,65,78,79,80]. In contrast, neither the Y^81^→M, T^353^→S, nor the S^158^→A substitutions were observed in the MAT1-1-1 and MAT1-2-1 protein variants coproduced by the 20 mycologically impure *O. sinensis* strains analyzed here.

The above observations indicate that the 20 *O. sinensis* strains simultaneously produced naturally paired MAT1-1-1 and MAT1-2-1 protein variants, which exhibited N- and/or C-terminal truncations together with diverse amino acid substitutions at distinct sites. These findings underscore the extensive genetic variation within the MAT loci of distinct fungal genomes residing within the impure *O. sinensis* cultures, irrespective of their designation as pure strains previously reported by Li et al. [36]. Unlike the variable presence/absence patterns detected across the genome assemblies of the 6 *H. sinensis* strains, all 20 *O. sinensis* strains analyzed in this study consistently co-expressed MAT1-1-1 and MAT1-2-1 proteins as natural pairs with divergent domain architectures. Given that no repetitive copies of the *MAT1-1-1* or *MAT1-2-1* genes exist in the *H. sinensis* genome [69], the differential pairing patterns of MAT1-1-1 and MAT1-2-1 proteins observed herein are compatible with contributions from multiple co-occurring fungal taxa to the observation of differential natural pairings of the mating proteins within these strains.

## 3. Discussion

### 3.1. The DNA-Binding Domains of Mating Proteins Coproduced in Pairs by O. sinensis Strains Adopt Altered Tertiary Structures

Li et al. [67,68] reported the differential occurrence and expression of *MAT1-1-1*, *MAT1-2-1*, and pheromone receptor genes in *H. sinensis*. Full-length MAT1-1-1 and MAT1-2-1 proteins and their DNA-binding domains have also been shown to adopt diverse stereostructures and to cluster into multiple Bayesian clusters/branches [65,69]. Taken together, these genetic, transcriptional, and protein-structural findings are inconsistent with a self-fertilizing reproductive model but consistent with self-sterility for *O. sinensis*, potentially involving heterothallic mating, hybrid reproduction, or parasexual processes during the lifecycle of the *C. sinensis* insect–fungal complex [65,67,68,69].

The present study examined the differential natural pairing patterns of MAT1-1-1 and MAT1-2-1 protein variants simultaneously coproduced by each of 20 *O. sinensis* strains. The MATα_HMGbox and HMG-box_ROX1-like domains of these proteins exhibit diverse N- and/or C-terminal truncations and 1–4 amino acid substitutions (Figure 4, Figure 5 and Figures S1–S10; Table 2). The identified variations are associated with changes in hydrophobicity and secondary/tertiary structures, including the altered 3D conformations of the hydrophobic cores formed by 3 α-helices of each functional domain.

The HMGbox domains in mating proteins are multifunctional motifs that play essential roles in the transcriptional regulation of mating-related genes, *O. sinensis* sexual reproduction, and host adaptation within the *C. sinensis* insect-fungal complex [58,59,65,66,67,68,69]. A conservative continuation model has proposed that the MATα-HMGBox domain of the MAT1-1-1 protein derives from a conserved ancestral mating-type HMG lineage, whereas the HMG-box_ROX1-like domain of the MAT1-2-1 protein reflects evolutionary recruitment of a distinct ROX1-like regulatory lineage through functional convergence [62,63,83,84,85,86].

The MATα_HMGbox and HMG-box_ROX1-like domains of the mating proteins each contain a hydrophobic core assembled by 3 tightly packed α-helices in an asymmetric configuration. These helices fold into a characteristic L-shaped architecture, conferring structural stability and tolerance against conformational perturbations that can trigger domain unfolding or distortion [73,87,88]. This conformation enables high-affinity, sequence-dependent binding to short AT-rich motifs located within the promoter regions of target mating-related genes [61,89,90,91,92,93,94]. This interaction is established as the initial step in the transcriptional regulation of mating-type cascades, governing mating identity and gene expression related to sexual reproduction [62,83,95,96].

Although the DNA-binding domains of MAT1-1-1 and MAT1-2-1 proteins belong to the HMG-box superfamily and share the characteristic L-shaped architecture, they are associated with distinct target-gene regulatory programs in mating-type regulation. The MATα_HMGbox domain primarily activates *MAT1-1*-associated transcriptional pathways as a transcriptional activator of cognate mating-type genes. In contrast, the HMG-box_ROX1-like domain recognizes motifs within *MAT1-2*-associated promoters, indicating both the repression of genes associated with the opposite mating type (e.g., “a-specific” genes) and the activation of *MAT1-*2-specific transcriptional programs. Thus, these DNA-binding domains may act through complementary regulatory pathways that coordinate mating-type specification and downstream reproductive development [96,97,98,99,100]. Therefore, the MATα_HMGbox and HMG-box_ROX1-like domains regulate complementary target gene sets that establish mating-type identity during the sexual reproduction of *O. sinensis*.

It has been hypothesized that the MAT1-1-1 and MAT1-2-1 proteins may form heterodimers that promote outbreeding and initiate the sexual cycle in heterothallic fungi [101,102,103]; however, direct experimental evidence in *O. sinensis* remains limited. The interplay of the functional domains of 2 mating proteins may be mediated through complementary electrostatic properties, providing a conceivable basis for cooperative DNA binding. Such in vivo interactions may facilitate the transition from asexual growth to sexual development by modulating DNA-binding specificity and downstream transcriptional programs associated with mating and meiosis in *O. sinensis* [104]. Relative to the reference MAT1-1-1 and MAT1-2-1 proteins in pure *H. sinensis*, these structural variations in naturally paired mating proteins revealed in the present study may indicate their heterogeneous fungal origins and alter the precise recognition of target DNA segments and thus affect transcriptional regulation, with either reduced or enhanced activity of mating-type-specific genes that coordinate heterothallic or hybrid reproduction throughout the lifecycle of the *C. sinensis* insect–fungal complex. Thus, experimental validation through protein biochemistry and reproductive physiology studies remains necessary to evaluate this hypothesis, clarify species-specific reproductive mechanisms, and determine how structural variations in mating proteins reduce or enhance complementary interactions and mating function in *O. sinensis* [65,66,67,68,69]. Such investigations are particularly important given that the genome-independent and multigenotypic nature of *O. sinensis* is not yet fully understood [9,10,11,12,13,19,24,27,30,35,37,38,54,105].

### 3.2. Complex Phylogenetic Heterogeneity of O. sinensis Strains

Bushley et al. [14] used a dual-fluorescence microscopy approach and observed multicellular heterokaryotic microstructures within *C. sinensis* ascospores and hyphae, including mono-, bi-, tri-, and tetra-nucleate cellular configurations. Zhang & Zhang [72] questioned whether such polynucleate cells might inconsistently harbor heterogeneous hereditary materials. Related observations from culture-dependent and culture-independent studies [12,13,36,37,38] have revealed the presence of multiple fungal components within *C. sinensis* ascospores and ascocarps and even in some strains described as pure *H. sinensis*. These findings support the hypothesis proposed by Zhang & Zhang [72] that multicellular heterokaryotic cells may contain heterogeneous hereditary substances.

For phylogenetic assessment, as shown in Table 1, Li et al. [36] deposited ITS sequences for 15 of the 20 *O. sinensis* strains in GenBank but not for the remaining five strains. Among the 15 sequenced strains, only Group-A (GC-biased Genotype #1 *H. sinensis*) ITS sequences were reported for 10 strains, whereas both Group-A and Group-C (AT-biased Genotype #17) ITS sequences were reported for the remaining 5 strains (Table 1, Tables S1 and S2) [9,10,12,13,36,37]. The culture-dependent methodology employed by Li et al. [36] included (1) 4 primer pairs for ITS amplification (Table S4), (2) different annealing temperatures (53 °C, 55 °C, and 60 °C) used for different primer pairs, and (3) direct sequencing of mixed amplicon pools. The comparative results presented in Table S4 indicate differences in primer similarity and coverage among representative GC- and AT-biased *O. sinensis* genotypes and are consistent with variable amplification efficiencies under the reported experimental conditions [36]. Accordingly, some co-occurring fungal taxa or low-abundance ITS amplicons present in mixed samples may not have been fully captured. In addition, because culture-dependent methods do not recover nonculturable fungi and because Li et al. [36] used direct sequencing of mixed amplicons rather than extensive amplicon cloning–sequencing with large colony sampling, low-abundance ITS variants may have been underrepresented [36,37]. Notably, Table S4 also shows identical primer similarity and coverage values for the ITS5/ITS4 primer pair when aligned with Genotypes #1 and #13–14, which have previously been reported to co-occur in *C. sinensis* ascospores [13]. This observation raises the possibility that low-abundance Genotypes #13–14 sequences could also have been coamplified in certain ascospore-derived strains.

In contrast, Li et al. [12,13,37] employed culture-independent approaches that included multiple primer pairs, touch-down PCR protocols, cloning–sequencing with sampling of >30 white colonies, and MALDI-TOF SNP mass spectrum genotyping using multiple extension primers to characterize the fungal composition of *C. sinensis* ascospores and strains described as pure *H. sinensis*. These studies have revealed the co-occurrence of psychrophilic, genome-independent GC-biased Genotypes #1 and #13–14 and AT-biased Genotypes #5–6 and #16 of *O. sinensis*, and mesophilic *Samsoniella hepiali* (formerly classified as *Paecilomyces hepiali*) [106]. Together with the methodological considerations noted above, these findings suggest that certain lower-abundance amplicons and nonculturable taxa may not have been captured in the study conducted by Li et al. [36].

Li et al. [36] proposed that all 20 *O. sinensis* strains represented pure cultures and interpreted the detected AT-biased sequences as repetitive ITS copies as nonfunctional pseudogenes within a single GC-biased *H. sinensis* genome. Li et al. [107,108] further hypothesized that nonfunctional ITS or rRNA pseudogenes may arise through repeat-induced point (RIP) mutation. However, other studies have indicated that GC- and AT-biased Genotypes #2–17 of *O. sinensis* are absent from the genome assemblies of 5 *H. sinensis* strains and instead represent genome-independent lineages, despite these *O. sinensis* genotypes possibly sharing a common heredity ancestor [9,10,11,12,13,24,38,64,74,78,79,80,81]. Multiple *O. sinensis* genotypes have also been reported to differentially coexist within different compartments of the *C. sinensis* insect-fungal complex, with their relative abundances varying dynamically and reciprocally during *C. sinensis* maturation, thereby providing additional evidence supporting the genomically independent nature of these genotypes [9,10,11,12,13,27,30,35,37]. In summary, these findings are inconsistent with the pseudogene hypothesis that AT-biased sequences represent RIP-derived repeats within a single genome of GC-biased Genotype #1 *H. sinensis*. Instead, they suggest that these GC- and AT-biased genotypic sequences represent genome-independent fungal taxa.

### 3.3. Heterogeneous Fungal Sources of MAT1-1-1 and MAT1-2-1 Proteins Simultaneously Produced in Paired Form by the Analyzed O. sinensis Strains

#### 3.3.1. Inconsistency Between Co-Occurring Mating Proteins in Purportedly “Pure” *O. sinensis* Strains and the Differential Presence of Mating-Type Genes in Pure *H. sinensis* Strains

As analyzed in Section 2.6, the *MAT1-1-1* and *MAT1-2-1* genes exhibit differential presence/absence patterns across the genome assemblies of Genotype #1 *H. sinensis* strains [64,69,78,79,80,81,82]. In contrast, the present study revealed the simultaneous production of MAT1-1-1 and MAT1-2-1 proteins in pairs across all 20 *O. sinensis* strains, despite substantial variations in N- and/or C-terminal truncations and amino acid substitutions among these proteins (Table 2; Figure 3) [14,36]. The observed differential natural pairing patterns among highly divergent MAT1-1-1 and MAT1-2-1 variants are not consistent with derivation from a single uniform genetic background. When considered together with the methodological issues discussed in Section 2.3 and Section 3.2, these protein-level observations are compatible with contributions from multiple co-occurring fungal taxa (including the identified *O. sinensis* genotypes and taxa that may not be fully captured using culture-dependent approaches) to the pool of mating proteins detected in these cultures. Further unbiased, culture-independent characterization may help clarify the composition and co-occurrence patterns of genomically independent *O. sinensis* genotypes and heterospecific fungal taxa across these strains [12,13,27,35,37].

#### 3.3.2. Potential Heterogeneity Among Reported Pure *O. sinensis* Strains

The highly anticipated challenge concerns the strain CS68-2-1229 in Group I (highlighted in dark green and underlined in Figure 1 and Figure 2), which reportedly contains only GC-biased Genotype #1 in this monoascospore-derived culture [36]. Two MAT1-1-1 protein sequences are available in GenBank for this strain: AGW27560 (full-length; 372 amino acids) and AGW27528 (truncated; 301 amino acids) (Figures S1 and S3). These 2 sequences are 100% identical throughout their shared region, whereas AGW27528 exhibits 81% query coverage relative to the authentic full-length MAT1-1-1 protein because of 63- and 8-residue truncations at the N- and C-termini, respectively (Table 2), which is consistent with amplification using different primer sets targeting *MAT1-1-1* transcripts. In addition, this strain was reported to simultaneously produce the truncated MAT1-2-1 protein AGW27548 (175 amino acids; Figure 2, Figures S2 and S4), which shares 99.4% sequence similarity with the authentic full-length MAT1-2-1 protein but displays only 70% sequence coverage because of a 74-residue C-terminal truncation relative to the authentic 249-amino-acid MAT1-2-1 protein (Table 2; Figure S2). Notably, all 3 mating protein records from CS68-2-1229 were released in GenBank on the same date (28-SEP-2013). As shown in Figure 1, the MATα_HMGbox domain (162 amino acids) of AGW27528 is among the 9 longest truncated MAT1-1-1 proteins. In contrast, the HMG-box_ROX1-like domain (49 amino acids) of AGW27548 is 10 residues shorter than the longest truncated HMG-box_ROX1-like domain (59 amino acids) observed in AGW27543 (Figure 2 and Figure S2) [65,70]. Moreover, AGW27548 has a tyrosine^144^→histidine^144^ (Y^144^→H^144^) substitution and exhibits altered secondary and tertiary structural characteristics in the AlphaFold 3D model U3N9W5 (Figures S2 and S4; Table 1 and Table 2). Because no repetitive copies of the *MAT1-1-1* or *MAT1-2-1* genes are present in the genome assemblies of 6 *H. sinensis* strains [69], these observations suggest that AGW27528 may be encoded by the authentic *MAT1-1-1* gene in the *H. sinensis* genome, whereas AGW27548 may have been derived either from a variant *MAT1-2-1* gene within the same *H. sinensis* genome or from a genome-independent taxon co-occurring within the CS68-2-1229 culture. Whether strain CS68-2-1229 represents a single-taxon culture with the significantly variant *MAT1-2-1* gene or instead contains multiple fungal taxa warrants re-evaluation.

Among the 10 purportedly homogeneous *O. sinensis* strains that reportedly contain only GC-biased Genotype #1 *H. sinensis* (Table 1), strain CS561-964 in Group II represents another special example (Table 2) [14,36]. It produced the MAT1-1-1 protein AGW27526, which contains 63- and 13-residue truncations at the N- and C-termini, respectively, together with amino acid substitutions (CDRA^65–68^→SSFT^2–5^) within the MATα_HMGbox domain (Figure 1, Figures S1 and S10; Table 2). The authentic peptide segment “CDRA^65–68^” of the reference MAT1-1-1 AGW27560 (encoded by the DNA segment “TGTGATCGAGCG” in the *MAT1-1-1* gene KC437356) is replaced with the variant peptide segment “SSFT^2–5^” in AGW27526 (encoded by the DNA segment “TCCTCTTTCACG” in the *MAT1-1-1* gene KC429528). In combination with the N- and C-terminal truncations, these sequence variations suggest that AGW27526 was derived from a genome-independent, co-occurring fungal taxon within the CS561-964 culture rather than from a single Genotype #1 *H. sinensis*; the ITS sequences associated with the contributing taxon may not have been captured by Li et al. [36], given the methodological limitations discussed in Section 2.3 and Section 3.2.

Re-analysis of the previously reported homogeneous *O. sinensis* strains raises discussions concerning fungal purification, in vitro culture conditions, and unbiased, comprehensive mycological and molecular characterization of co-occurring fungal taxa in such cultures. (1) Do the homogeneous strains listed in Table 1 genuinely harbor only GC-biased Genotype #1, or do they contain additional fungal taxa? (2) Do the strains previously reported to contain Genotypes #1 and #17 (Tables S1 and S2) also contain additional co-occurring taxa that were not captured under the culture-dependent settings? One plausible interpretation is that at least some of the differentially naturally paired MAT1-1-1 and MAT1-2-1 proteins, exhibiting various truncations and diverse amino acid substitutions, may not necessarily be produced by GC-biased Genotype #1 *H. sinensis*. Instead, they may originate from additional co-occurring fungal taxa that were not captured under the culture-dependent and suboptimal experimental conditions employed by Li et al. [36]. Thus, such co-occurring taxa may symbiotically accomplish heterothallic or hybrid reproduction-related processes during the lifecycle of the *C. sinensis* insect-fungal complex.

### 3.4. Co-Occurring Fungal Taxa as Potential Mating Partners for Self-Sterile O. sinensis During Heterothallic or Hybrid Reproduction

*H. sinensis* (GC-biased Genotype #1 of *O. sinensis*) was previously postulated to be the sole anamorph of the *O. sinensis* teleomorph [45]. The anamorphic name *H. sinensis* was replaced by the teleomorphic name *O. sinensis* in accordance with the IMA’s “One Fungus = One Name” nomenclature principle [49,51,52,53]. Although the longstanding fungal taxonomic name *C. sinensis* was revised to *O. sinensis* in 2007 using the *H. sinensis* strain EFCC7287 as the nomenclature reference [48], the new name *O. sinensis* has been uniformly applied to all 17 genomically independent genotypes of *O. sinensis*, including 11 GC-biased and 6 AT-biased genotypes that may share a common ancestral origin [9,10,24,48,49,50].

To support the sole anamorph hypothesis for *H. sinensis* [45], *O. sinensis* has been proposed to be self-fertile (homothallic or pseudohomothallic) on the basis of the findings of both the *MAT1-1-1* and *MAT1-2-1* genes within the genome assemblies of 2 *H. sinensis* strains [14,64]. However, several studies [64,109,110] have reported unsuccessful attempts to artificially cultivate fruiting bodies and ascospores of *C. sinensis* insect–fungal complexes using single-species fungal cultures. Zhang et al. [51] summarized the multidecade history of unsuccessful cultivation attempts performed in academic research-oriented settings, and Qin et al. [111] discussed technical obstacles associated with artificial cultivation of *C. sinensis* fruiting bodies and ascospores. The repeated failures have been attributed, at least partially, to the extremely low and unstable infection rates of *H. sinensis* [37,112,113,114]. In contrast to the nearly noninfectious characteristics reported for pure *H. sinensis* cultures, Li et al. [37] advanced an alternative inoculation strategy employing cocultures composed of natural fungal clusters isolated directly from the intestines of living *Hepialus lagii* larvae. These cocultures reflected the native abundance ratios of multiple co-occurring taxa, including several GC- and AT-biased genotypes of *O. sinensis* together with *S. hepiali*. This coculture-based approach (n = 100) resulted in up to 39-fold higher infectivity against *Hepialus armoricanus* larvae than did conidia or mycelia (n = 100 each group) from pure *H. sinensis* (*p* < 0.001). Recent studies have further explored the microbial interactions during fungal infection of bat moth larvae [115,116,117]. These observations suggest that single-species cultivation strategies are likely insufficient to reproduce the complete lifecycle of *C. sinensis* under artificial cultivation conditions and therefore motivate a re-evaluation of the contentious sole-anamorph and self-fertilization hypotheses for *H. sinensis* in accordance with all 4 criteria of Koch’s postulates.

With respect to reproductive strategies, homothallic or pseudohomothallic reproduction has been proposed for *O. sinensis* [14,64]. However, subsequent investigations revealed extensive genetic diversity within *O. sinensis*, including at least 17 genome-independent genotypes representing distinct fungal lineages [9,10,12,13,24,27,30,35,54]. Zhang and Zhang [72] and Li et al. [67,68] reported the differential occurrence and expression of *MAT1-1-1*, *MAT1-2-1*, and pheromone receptor genes across >200 wild-type *C. sinensis* isolates and *H. sinensis* strains. More recent studies [65,69] have demonstrated heteromorphic tertiary structures among variant MAT1-1-1 and MAT1-2-1 proteins, particularly variations within their DNA-binding domains. The results of the present study further revealed differential natural pairing patterns of the mating proteins with diverse truncations and various amino acid substitutions that were simultaneously coproduced by each of the 20 *O. sinensis* strains. The observations of differential natural pairings of the variant mating proteins suggest divergent origins for the proteins, involving at least GC-biased Genotypes #1 and #3 and AT-biased Genotype #17 (or #5) of *O. sinensis* [65]. Because the *MAT1-1-1* and *MAT1-2-1* genes each occur as a single copy within the *H. sinensis* genome with no repetitive copies [69], these structural observations provide protein-level evidence supporting heterogeneous fungal origins of mating-type loci among numerous wild-type *C. sinensis* isolates and *O. sinensis* strains. In brief, the available genetic, transcriptional, and protein structural evidence is inconsistent with a self-fertilization reproductive model for *O. sinensis* and instead consistent with self-sterility, which requires heterothallic mating, hybridization, or parasexual outcrossing to accomplish sexual reproduction [13,65,67,68,69,118,119,120,121,122,123,124,125,126].

A species discrepancy between the GC-biased *H. sinensis* strains used as inoculants and an AT-biased Genotype #4 teleomorph detected in cultivated fruiting bodies was observed in the successful industrial cultivation of *C. sinensis* insect–fungal complexes [46]. Notably, AT-biased *O. sinensis* sequences are absent from genome assemblies ANOV00000000, JAAVMX000000000, LKHE00000000, LWBQ00000000, and NGJJ00000000 of the *H. sinensis* strains Co18, IOZ07, 1229, ZJB12195, and CC1406-20395, respectively [9,10,11,12,13,24,30,35,36,37,38,64,74,78,79,80,81]. Moreover, multiple studies have provided genomic and transcriptomic evidence supporting the genome-independent nature of *O. sinensis* genotypes [27,30,35,36,37,38,74]. These findings do not support the hypotheses that AT-biased genotypes represent nonfunctional ITS or rRNA pseudogenes generated through RIP mutation within a single GC-biased *H. sinensis* genome [36,107,108]. Therefore, the success of industrial cultivation, together with the inoculant/teleomorph discrepancy, suggests that the sole-anamorph hypothesis for *H. sinensis* may not satisfy all 4 criteria of Koch’s postulates.

Subsequently, Li et al. [112] reinterpreted the inoculant used in the industrial cultivation project as a conidial suspension derived from cultures of *C. sinensis* ascospores, thereby revising the previous report that the inoculant was pure *H. sinensis* strains [46]. The revised report, however, did not provide a detailed description of the fungal components of the inoculant or clarify the teleomorphic fungus/fungi in the cultivated insect–fungal complexes. Interestingly, several independent studies failed to detect AT-biased Genotype #4 in *C. sinensis* ascospores [9,12,13,36,37], arguing whether Genotype #4 indeed participates in the sexual reproduction of *O. sinensis*. Thus, the inoculant–teleomorph discrepancy reported in cultivated insect–fungal complexes [46] remains unresolved on the basis of the currently available evidence.

Several observations support the use of multifungal cocultures as inoculants as a key factor in the industrial success of cultivating insect–fungal complexes. (1) Li et al. [112] described the use of an inoculant derived from cultures of *C. sinensis* ascospores in an effort to overcome the weak infectivity of pure *H. sinensis*, and other studies have demonstrated that *C. sinensis* ascospores harbor multiple genotypic and heterospecific fungal taxa [12,13,36]. (2) AT-biased Genotype #4 of *O. sinensis* is absent in *C. sinensis* ascospores [10,12,13,36,38]. Thus, the sole Genotype #4 teleomorph in the cultivated *C. sinensis* insect–fungal complex [46] may instead originate from the coculture of additional components of the natural complex, such as the stroma and stromal fertile portion that indeed harbor Genotype #4, alongside other fungal taxa [9,10,12,13,24,30,35,105]. Consistent with this possibility, Li et al. [37] reported that compared with the conidia and mycelia of pure *H. sinensis*, cocultures containing multiple genotypic and heterospecific fungal taxa derived from the intestine of living *Hepialus lagii* larvae significantly increased the infectivity to *H. armoricanus* larvae. (3) The industrial cultivation system was supplemented with soil collected from natural *C. sinensis* production regions on the Qinghai-Tibet Plateau [46], introducing additional environmental microbial components [29,127,128].

Because reports on the purification of GC- and AT-biased Genotypes #2–17 of *O. sinensis* are currently limited, the genomic and transcriptomic characterization of diverse *MAT1-1-1* and *MAT1-2-1* genes across genome-independent *O. sinensis* genotypes remain unknown, particularly with respect to structural variation within key DNA-binding domains. Naturally paired mating proteins are widely regarded as key determinants of mating compatibility and the initiation of fungal sexual reproduction [56,57,58,59,60]. Evidence demonstrating the differential occurrence and expression of mating-type and pheromone receptor genes in *H. sinensis* suggests self-sterility rather than self-fertilization for *O. sinensis* [14,45,63,66,67,68,69]. Furthermore, observations of heteromorphic stereostructures within variant MATα_HMGbox (MAT1-1-1) and HMG-box_ROX1-like (MAT1-2-1) domains provide additional protein-level support for models that favor heterothallic or hybrid reproduction over strict homothallism, involving divergent symbiotic fungal taxa within heterogeneous cultures. Thus, self-sterility offers a trustworthy explanation for the repeated inability of single-species cultures to reproduce key lifecycle stages under artificial conditions and further underscores the need for continued investigation of multi-fungal symbiosis across both natural and artificially cultivated *C. sinensis* insect–fungal complexes.

## 4. Materials and Methods

### 4.1. MAT1-1-1 and MAT1-2-1 Protein Sequences from O. sinensis Strains

AI-predicted 3D structural morphs for 20 truncated MAT1-1-1 proteins (AGW27517–AGW27536) derived from 20 *O. sinensis* strains were obtained from the AlphaFold database (https://alphafold.com/, Cambridgeshire, UK; accessed from 18 June 2025 to 10 July 2026) (Table 1) [14]. These strains simultaneously produced MAT1-2-1 proteins (AGW27537–AGW27556). Predicted 3D structural morphs for 5 truncated and 6 full-length MAT1-2-1 proteins, but not for the remaining 9 full-length MAT1-2-1 proteins, were also obtained from the AlphaFold database.

Li et al. [36] reported that 11 of the 20 *O. sinensis* strains were obtained from fresh caterpillar body samples (marked as “TS” in Table 1) harvested from various *C. sinensis* production regions on the Qinghai-Tibet Plateau. The remaining 9 strains were from the culture of monoascospores of *C. sinensis* insect-fungal complexes (marked as “SS” in Table 1) harvested from Maqên, Guoluo, Qinghai Province, China.

The MAT1-1-1 protein sequences were encoded by the *MAT1-1-1* cDNAs, which were amplified from total RNA extracted from the *O. sinensis* strains using the primer pair *m1F3/m1R3*, while the *MAT1-2-1* cDNAs were amplified using the primer pair *Mat1-2F*/*Mat1-2R* [14].

Li et al. [36] deposited ITS sequences in GenBank for 15 of the 20 *O. sinensis* strains for phylogenetic positioning and reported the detection of only Group-A ITS sequences (GC-biased Genotype #1 of *O. sinensis*) for 10 *O. sinensis* strains (shown in brown in Table 1). For 5 other strains (shown in red in Table 1), they reported the ITS sequences from both Group-A (GC-biased Genotype #1) and Group-C (AT-biased Genotype #5). The remaining 5 *O. sinensis* strains shown in black in Table 1 lack ITS sequence information in GenBank. Tables S1 and S2 show the alignment results between the ITS sequences from the *O. sinensis* strains reported by Li et al. [36] and the representative ITS sequences of the GC- and AT-biased *O. sinensis* genotypes.

### 4.2. Alignment of the DNA-Binding Domain Sequences of the Mating Proteins

The amino acid sequences of the MAT1-1-1 and MAT1-2-1 proteins were aligned using the GenBank Blastp program (https://blast.ncbi.nlm.nih.gov/ (Bethesda, MD, USA), accessed from 18 June 2025 to 10 July 2026).

### 4.3. Amino Acid Properties and Scale Analysis

The amino acid sequences of the DNA-binding domains of the mating proteins were scaled according to the general physicochemical properties of their side chains using the ExPASy ProtScale tool (https://web.expasy.org/protscale/; Basel, Switzerland; accessed from 18 May 2025 to 10 July 2026) (Table S3) [65,68,69,73,129,130,131,132]. The MATα_HMGbox domain of MAT1-1 proteins (amino acids 51→225 of the reference sequence AGW27560 derived from the *H. sinensis* strain CS68-2-1229 [14]) and the HMG-box_ROX1-like domain of the MAT1-2-1 proteins (amino acids 127→197 of the reference sequence AEH27625 derived from the *H. sinensis* strain CS2 [70]) were each extended by 9 additional amino acid residues upstream and downstream of the domains to ensure complete domain coverage. These sequences were then plotted sequentially using the ExPASy ProtScale algorithm with a window size of 9 amino acid residues and a linear weight variation model [65,68,69,73,130,131] to generate ExPASy ProtScale plots and predict variations in the hydrophobic properties (hydropathy index) and secondary structural features, including α-helices, β-sheets, β-turns, and random coils. The topological profiles and waveform patterns derived from the ProtScale outputs were compared to characterize variations in hydrophobicity and 2D structural properties within the DNA-binding domains of the mating proteins. For improved comparability and readability, red dashed auxiliary lines and directional arrows were superimposed onto the ProtScale plots to highlight subtle topological profile shifts and waveform changes between variant DNA-binding domains and authentic reference proteins.

### 4.4. Tertiary Structures of the Mating Proteins Predicted by AlphaFold and Superposition Analysis of the 3D Structures

To examine heteromorphic stereostructures in this study, the 3D structures of the MAT1-1-1 and MAT1-2-1 proteins from *O. sinensis* strains were predicted computationally from their amino acid sequences using AlphaFold, an artificial intelligence (AI)-driven machine learning platform [69,133,134,135,136,137,138,139,140,141,142]. The AlphaFold database reports per-residue model confidence using the predicted local distance difference test (pLDDT), which assigns a confidence score ranging from 0 to 100 to each residue [65,69,133,134,135,136,138,139,140,141,142,143].

Structural superposition for the AlphaFold-predicted MAT1-1-1 or MAT1-2-1 protein structures was performed using the Pairwise Structure Alignment tools provided by the RCSB Protein Data Bank (https://www.rcsb.org/alignment; accessed from 1 June 2026 to 31 July 2026) [75]. The Template-Model align (TM-align) algorithm was applied to the pairwise alignment of overall protein folding patterns of the reference and variant proteins [76]. The Smith-Waterman 3D (SW 3D) algorithm was used to optimize the resolution of the local folding similarity/dissimilarity of the locally magnified DNA-binding domains of the proteins and facilitate visualization of local structural differences [77].

### 4.5. Differential Natural Pairing of Variant MAT1-1-1 and MAT1-2-1 Proteins Simultaneously Produced by the 20 O. sinensis Strains and Correlations Among the Primary, Secondary, and Tertiary Structures of the DNA-Binding Domains

The MAT1-1-1 and MAT1-2-1 proteins that exhibit diverse structural variations with different N- and/or C-terminal truncations and various amino acid substitutions at different sites were assigned heteromorphic AlphaFold 3D structural models and naturally paired differentially. Natural pairs of the variant mating proteins simultaneously coproduced by each of the *O. sinensis* strains were compared to evaluate their potential complex fungal origins.

## 5. Conclusions

Across the 20 *O. sinensis* strains analyzed here, MAT1-1-1 and MAT1-2-1 protein variants exhibited diverse truncations and amino acid substitutions, particularly within their key DNA-binding domains. These primary structural variations were associated with corresponding changes in the hydrophobic properties and in the secondary and tertiary conformations of the differentially naturally paired, structurally divergent MAT1-1-1 and MAT1-2-1 proteins. Such structural heterogeneity among naturally paired mating proteins suggests that heterogeneous fungal sources co-occur within purportedly homogeneous *O. sinensis* strains. Together with previous genetic and transcriptional findings (including differential occurrence, alternative splicing, and variable transcription of the *MAT1-1-1*, *MAT1-2-1*, and pheromone receptor genes), the protein heterogeneity and differential natural pairing patterns identified here are more consistent with the participation of multiple co-occurring fungal taxa within these *O. sinensis* strains. These findings accord with the *O. sinensis* self-sterility and with reproductive models involving heterothallic mating or hybridization, rather than strictly homothallic or pseudohomothallic self-fertilization. Taken together, these findings refine the current understanding of *O. sinensis* sexual reproduction and further support the view that compatible mating partners and multitaxon synergistic symbioses are critical for sexual reproduction during the lifecycle of the *C. sinensis* insect–fungi complex on the Qinghai–Tibet Plateau.

## Figures and Tables

**Table 1:** *O. sinensis* strains and their tissue sources [36], GenBank accession numbers for the internal transcribed spacer (ITS) nucleic acid sequences and mating proteins, and AlphaFold UniProt model for the truncated and full-length MAT1-1-1 and MAT1-2-1 proteins derived from the *O. sinensis* strains.

*O. sinensis* Strain Code	Strain Source	GenBank Accession Number						AlphaFold Mating Protein UniProt Code	
		ITS Nucleotide Sequence			Protein Sequence				
		GC-Biased Group-A	AT-Biased Group-C		MAT1-1-1	MAT1-2-1		MAT1-1-1	MAT1-2-1
CS71-1219	SS	JQ900151	—		AGW27534	AGW27554		U3NE87 *	U3NEA9
CS91-1291	TS	JQ900166	—		AGW27523	AGW27543		U3N9T9 ⁋	U3N9W0
CS68-2-1229	SS	JQ900158	—		AGW27528	AGW27548		U3N9T9 ⁋	U3N9W5 ⁑
CS37-295	TS	JQ900169	—		AGW27519	AGW27539		U3N9T9 ⁋	D7F2E9 ♦
CS18-266	TS	JQ900168	—		AGW27520	AGW27540		U3N9T9 ⁋	—
CS560-961	TS	JQ900143	—		AGW27522	AGW27542		U3N6U0	D7F2E3
CS71-1218	SS	JQ900150	—		AGW27533	AGW27553		U3N6U8 †	U3N9X0
CS76-1284	TS	JQ900159	—		AGW27525	AGW27545		U3N919	—
CS561-964	TS	JQ900144	—		AGW27526	AGW27546		U3N7G5	—
CS25-273	TS	JQ900167	—		AGW27527	AGW27547		U3N6U4	—
CS71-1220	SS	JQ900152	JQ900175		AGW27535	AGW27555		U3N6U8 †	U3N9W5 ⁑
CS6-251	TS	JQ900163	JQ900180		AGW27517	AGW27537		U3N9T9 ⁋	U3N6V5
CS34-291	TS	JQ900162	JQ900179		AGW27524	AGW27544		U3N9T9 ⁋	—
CS70-1208	SS	JQ900146	JQ900172		AGW27529	AGW27549		U3NE79 ‡	—
CS68-2-1228	SS	JQ900157	JQ900178		AGW27536	AGW27556		U3N7H7	—
CS68-5-1216	SS	—	—		AGW27532	AGW27552		U3N6U8 †	U3N6W6
CS26-277	TS	—	—		AGW27521	AGW27541		U3N9T9 ⁋	D7F2E9 ♦
CS36-1294	TS	—	—		AGW27518	AGW27538		U3N9T9 ⁋	D7F2E9 ♦
CS70-1211	SS	—	—		AGW27530	AGW27550		U3NE79 ‡	—
CS70-1212	SS	—	—		AGW27531	AGW27551		U3NE87 *	—

Notes: The *O. sinensis* strains shown in brown possess ITS sequences belonging to GC-biased Group-A (GC-biased Genotype #1), those shown in red contain ITS sequences corresponding to both GC-biased Group-A and AT-biased Group-C (Genotype #17), and those shown in black do not have ITS sequences deposited in GenBank [14,36]. “TS” indicates that the *O. sinensis* strain was isolated from a caterpillar body sample, whereas “SS” indicates that the strain was obtained from cultures of monoascospores collected from mature *C. sinensis* insect-fungal complexes. ITS sequences shown in dark blue correspond to the GC-biased Group-A of *O. sinensis*, whereas those shown in light blue belong to the AT-biased Group-C of *O. sinensis* (Tables S1 and S2) [9,10,36]. The GenBank protein accession numbers shown in green correspond to full-length MAT1-2-1 protein sequences, whereas the protein accession numbers shown in pink denote truncated mating proteins. AlphaFold identifiers sharing the same suffix symbol (*, ⁋, †, ‡, ⁑, or ♦) indicate assignment to identical AlphaFold-predicted 3D structural morphotypes; identifiers lacking a suffix symbol indicate unique morphs. “—” indicates that the corresponding ITS sequences and/or AlphaFold-predicted 3D structural models are unavailable in the GenBank or AlphaFold database for the respective entry.

**Table 2:** The 20 *O. sinensis* strains were divided into two groups on the basis of the differential co-occurrent patterns of the paired MAT1-1-1 and MAT1-2-1 proteins with different truncations and amino acid substitutions.

Grp.	*O. sinensis* Strains	MAT1-1-1 Protein						MAT1-2-1 Protein			
		GenBank Accession #	Number of Residues Truncated at		Amino Acid Substitution	AlphaFold Identifier		GenBank Accession #	Number of Residue(s) Truncated at C Terminus	Amino Acid Substitution	AlphaFold Identifier
			N termin.	C termin.							
**I**	**CS71-1219**	AGW27534	110	8		U3NE87 *		AGW27554	65	Y^144^→H^144^, Q^185^→T^185^	U3NEA9
	**CS68-5-1216**	AGW27532	97	8		U3N6U8 **†**		AGW27552	75	Y^144^→H^144^	U3N6W6
	**CS71-1220**	AGW27535	97	8		U3N6U8 **†**		AGW27555	74	Y^144^→H^144^	U3N9W5 **⁑**
	**CS68-2-1229**	AGW27528	63	8		U3N9T9 **⁋**		AGW27548	74	Y^144^→H^144^	U3N9W5 **⁑**
	**CS91-1291**	AGW27523	63	8		U3N9T9 **⁋**		AGW27543	64	Y^144^→H^144^	U3N9W0
**II**	**CS37-295**	AGW27519	63	8		U3N9T9 **⁋**		AGW27539			D7F2E9 **♦**
	**CS36-1294**	AGW27518	63	8		U3N9T9 **⁋**		AGW27538			D7F2E9 **♦**
	**CS26-277**	AGW27521	63	8		U3N9T9 **⁋**		AGW27541			D7F2E9 **♦**
	**CS6-251**	AGW27517	63	8		U3N9T9 **⁋**		AGW27537		V^78^→I^78^, Y^144^→H^144^	U3N6V5
	**CS18-266**	AGW27520	63	8		U3N9T9 **⁋**		AGW27540		Y^144^→H^144^	−−
	**CS34-291**	AGW27524	63	8		U3N9T9 **⁋**		AGW27544		Y^144^→H^144^	−−
	**CS71-1218**	AGW27533	97	8		U3N6U8 **†**		AGW27553	1	Y^144^→H^144^, K^175^→X^175^	U3N9X0
	**CS70-1211**	AGW27530	97	8	F^97^→V^1^	U3NE79 **‡**		AGW27550		Y^144^→H^144^	−−
	**CS70-1208**	AGW27529	97	8	F^97^→V^1^	U3NE79 **‡**		AGW27549		Y^144^→H^144^	−−
	**CS70-1212**	AGW27531	110	8		U3NE87 *		AGW27551		Y^144^→H^144^	−−
	**CS560-961**	AGW27522	63	8	P^362^→L^298^	U3N6U0		AGW27542		Y^144^→H^144^	D7F2E3
	**CS76-1284**	AGW27525	66	10		U3N919		AGW27545		Y^144^→H^144^	−−
	**CS561-964**	AGW27526	63	13	CDRA^65−68^→ SSFT^2−5^	U3N7G5		AGW27546		Y^144^→H^144^	−−
	**CS25-273**	AGW27527	77	18		U3N6U4		AGW27547		Y^144^→H^144^	−−
	**CS68-2-1228**	AGW27536	95	8	S^95^→E^1^, L^98^ deleted	U3N7H7		AGW27556		Y^144^→H^144^	−−

Note: The *O. sinensis* strains shown in brown have only Group-A (GC-biased Genotype #1) ITS sequences reported, corresponding to GC-biased Genotype #1 of *O. sinensis*. The strains shown in red possess ITS sequences corresponding to both Group-A and Group-C (AT-biased Genotype #17). Those shown in black lack deposited ITS records in GenBank (Table 1, Tables S1 and S2) [9,10,14,36]. The GenBank protein accession numbers shown in pink denote truncated mating proteins, whereas those in green indicate full-length MAT1-2-1 proteins. **Group-I** strains simultaneously coproduce naturally paired truncated MAT1-1-1 and MAT1-2-1 proteins. **Group-II** strains coproduce truncated MAT1-1-1 proteins naturally paired with full-length MAT1-2-1 proteins. AlphaFold identifiers sharing the same suffix symbol (*, ⁋, †, ⁑, ‡, or ♦) correspond to identical predicted 3D structural morphotypes, whereas identifiers lacking a suffix symbol indicate unique structural morphs.

**Table 3:** Summary of the co-occurrence and differential occurrence of the MAT1-1-1 and MAT1-2-1 proteins in the *C. sinensis* insect-fungal complex, wild-type *C. sinensis* isolates, and *O. sinensis* strains listed in the GenBank database.

	Co-Occurrence of MAT1-1-1 and MAT1-2-1 Proteins	Differential Occurrence of Mating Proteins		Total
		MAT1-1-1	MAT1-2-1	
*C. sinensis* insect-fungal complexes	2 (33.3%)	2 (33.3%)	2 (33.3%)	6
Wild-type *C. sinensis* isolates	31 (20.5%)	85 (56.3%)	35 (23.2%)	151
*O. sinensis* strains of different genotypes	13 (46.4%)	11 (39.3%)	4 (14.3%)	28
**Total**	46 (25.0%)	97 (52.7%)	41 (22.3%)	184

## Data Availability

The original contributions presented in this study are included in the article/Supplementary Material. Further inquiries can be directed to the corresponding author.

## Supplementary Materials

The following supporting information can be downloaded at: https://www.mdpi.com/article/10.3390/ijms27167191/s1.
