# Supplementary material for: Structural Diversity and Differential Natural Pairings of MAT1-1-1 and MAT1-2-1 Proteins Essential for Sexual Reproduction in Ophiocordyceps sinensis Strains
Source: Int J Mol Sci. 2026 Aug 11;27(16):7191. doi: 10.3390/ijms27167191 (PMC13513268; doi:10.3390/ijms27167191)
Supplement: Supplementary file 1 [file ijms-27-07191-s001.zip › ijms-4393153-supplementary.pdf]

Structural diversity and differential natural pairings of MAT1-1-1 and MAT1-2-1 proteins essential for sexual reproduction in *Ophiocordyceps sinensis* strains

Xiu-Zhang Li [1,2], Yu-Ling Li [1], Wei Liu [3], Jian-Zhao Qi [4], and Jia-Shi Zhu [1,*]

[1] State Key Laboratory of Plateau Ecology and Agriculture, Qinghai Academy of Animal and Veterinary Sciences, Qinghai University, Xining, 810016, China; xiuzhang11@163.com, yulingli2000@163.com, zhujosh@163.com
[2] Qinghai Provincial Grassland Engineering Technology Research Center, Xining, 810016, China; xiuzhang11@163.com
[3] Institute of Immunology, Army Medical University, Chongqing, 400038, China; weiliu@tmmu.edu.cn
[4] Pharmacy, Northwest A&F University, Yangling, 712100, China; qjz@nwafu.edu.cn
* Correspondence: zhujosh@163.com (JSZ)

* Corresponding author

E-mail: zhujosh@163.com (JSZ)

```
AGW27560 1 MTTRNEVMQRLSSVRADVLLNFLTDDAIFQLASRYHESTTEADVLTPVSTAAASRATRQTKE  62
AGW27517
AGW27518
AGW27519
AGW27520
AGW27521
AGW27523
AGW27524
AGW27528
AGW27522
AGW27525
AGW27526
AGW27527
AGW27532
AGW27533
AGW27535
AGW27529
AGW27530
AGW27536
AGW27531
AGW27534

AGW27560 63 ASCDRAKRPLNAFMAFRSYYLKLFPDVQQKTASGFLTTLWHKDPFRNKWALIAKVYSFVRD  123
AGW27517 1   ------------------------------------------------------------  60
AGW27518 1   ------------------------------------------------------------  60
AGW27519 1   ------------------------------------------------------------  60
AGW27520 1   ------------------------------------------------------------  60
AGW27521 1   ------------------------------------------------------------  60
AGW27523 1   ------------------------------------------------------------  60
AGW27524 1   ------------------------------------------------------------  60
AGW27528 1   ------------------------------------------------------------  60
AGW27522 1   ------------------------------------------------------------  60
AGW27525 1   ------------------------------------------------------------  60
AGW27526 1   -SSFT-------------------------------------------------------  60
AGW27527 1                 ----------------------------------------------  46
AGW27532 1                                     --------------------------  26
AGW27533 1                                     --------------------------  26
AGW27535 1                                     --------------------------  26
AGW27529 1                                    V--------------------------  27
AGW27530 1                                    V--------------------------  27
AGW27536 1                                  E-- -------------------------  28
AGW27531 1                                                 -------------  13
AGW27534 1                                                 -------------  13

AGW27560 124 QIGKDKVSLSYFMSLACPTMTIIEPAAYLNALGWCVQDDDAGSQKLFQDESSANLDQSSL  183
AGW27517 61  ------------------------------------------------------------  120
AGW27518 61  ------------------------------------------------------------  120
AGW27519 61  ------------------------------------------------------------  120
AGW27520 61  ------------------------------------------------------------  120
AGW27521 61  ------------------------------------------------------------  120
AGW27523 61  ------------------------------------------------------------  120
AGW27524 61  ------------------------------------------------------------  120
AGW27528 61  ------------------------------------------------------------  120
AGW27522 61  ------------------------------------------------------------  120
AGW27525 61  ------------------------------------------------------------  120
AGW27526 61  ------------------------------------------------------------  120
AGW27527 47  ------------------------------------------------------------  106
AGW27532 27  ------------------------------------------------------------  86
AGW27533 27  ------------------------------------------------------------  86
AGW27535 27  ------------------------------------------------------------  86
AGW27529 28  ------------------------------------------------------------  87
AGW27530 28  ------------------------------------------------------------  87
AGW27536 29  ------------------------------------------------------------  88
AGW27531 14  ------------------------------------------------------------  73
AGW27534 14  ------------------------------------------------------------  73

AGW27560 184 LSAEYPSTEIELLSALVNIGYFPDHGADLVERMGSSHSGIMAPRAANCTPPVSYTKEKID  243
AGW27517 121 ------------------------------------------------------------  180
AGW27518 121 ------------------------------------------------------------  180
AGW27519 121 ------------------------------------------------------------  180
AGW27520 121 ------------------------------------------------------------  180
AGW27521 121 ------------------------------------------------------------  180
AGW27523 121 ------------------------------------------------------------  180
AGW27524 121 ------------------------------------------------------------  180
AGW27528 121 ------------------------------------------------------------  180
AGW27522 121 ------------------------------------------------------------  180
AGW27525 121 ------------------------------------------------------------  180
AGW27526 121 ------------------------------------------------------------  180
AGW27527 107 ------------------------------------------------------------  166
AGW27532 87  ------------------------------------------------------------  146
AGW27533 87  ------------------------------------------------------------  146
AGW27535 87  ------------------------------------------------------------  146
AGW27529 88  ------------------------------------------------------------  147
AGW27530 88  ------------------------------------------------------------  147
AGW27536 69  ------------------------------------------------------------  148
AGW27531 74  ------------------------------------------------------------  133
AGW27534 74  ------------------------------------------------------------  133
```

```
AGW27560 244 FINTIRSDPVQATKEILGDCYDETTIKLLGVKSHNVESVDSITHLSMQREYQAPRFFYDY 304
AGW27517 181 ------------------------------------------------------------ 240
AGW27518 181 ------------------------------------------------------------ 240
AGW27519 181 ------------------------------------------------------------ 240
AGW27520 181 ------------------------------------------------------------ 240
AGW27521 181 ------------------------------------------------------------ 240
AGW27523 181 ------------------------------------------------------------ 240
AGW27524 181 ------------------------------------------------------------ 240
AGW27528 181 ------------------------------------------------------------ 240
AGW27522 181 ------------------------------------------------------------ 240
AGW27525 181 ------------------------------------------------------------ 240
AGW27526 181 ------------------------------------------------------------ 240
AGW27527 167 ------------------------------------------------------------ 226
AGW27532 147 ------------------------------------------------------------ 206
AGW27533 147 ------------------------------------------------------------ 206
AGW27535 147 ------------------------------------------------------------ 206
AGW27529 148 ------------------------------------------------------------ 207
AGW27530 148 ------------------------------------------------------------ 207
AGW27536 149 ------------------------------------------------------------ 208
AGW27531 134 ------------------------------------------------------------ 193
AGW27534 134 ------------------------------------------------------------ 193

AGW27560 305 SVSYAGMDFGGSNEPVMNLNNLPENETFDIDSPFDLDKILGQSQSEGERTSHLPPSPPHN 364
AGW27517 241 ------------------------------------------------------------ 300
AGW27518 241 ------------------------------------------------------------ 300
AGW27519 241 ------------------------------------------------------------ 300
AGW27520 241 ------------------------------------------------------------ 300
AGW27521 241 ------------------------------------------------------------ 300
AGW27523 241 ------------------------------------------------------------ 300
AGW27524 241 ------------------------------------------------------------ 300
AGW27528 241 ------------------------------------------------------------ 300
AGW27522 241 ---------------------------------------------------------L-- 300
AGW27525 241 -----------------------------------------------------------  299
AGW27526 241 --------------------------------------------------------     296
AGW27527 227 ---------------------------------------------------          277
AGW27532 207 ------------------------------------------------------------ 266
AGW27533 207 ------------------------------------------------------------ 266
AGW27535 207 ------------------------------------------------------------ 266
AGW27529 208 ------------------------------------------------------------ 267
AGW27530 208 ------------------------------------------------------------ 267
AGW27536 209 ------------------------------------------------------------ 268
AGW27531 194 ------------------------------------------------------------ 254
AGW27534 194 ------------------------------------------------------------ 254

AGW27560 365 PLDFYFAF                                                     372
AGW27517 301 -                                                            301
AGW27518 301 -                                                            301
AGW27519 301 -                                                            301
AGW27520 301 -                                                            301
AGW27521 301 -                                                            301
AGW27523 301 -                                                            301
AGW27524 301 -                                                            301
AGW27528 301 -                                                            301
AGW27522 301 -                                                            301
AGW27525 299
AGW27526 296
AGW27527 277
AGW27532 267 -                                                            267
AGW27533 267 -                                                            267
AGW27535 267 -                                                            267
AGW27529 268 -                                                            268
AGW27530 268 -                                                            268
AGW27536 269 -                                                            269
AGW27531 254
AGW27534 254
```

Figure S1. Alignment of the sequences of the query MAT1-1-1 protein AGW27560 derived from the *H. sinensis* strain CS68-2-1229 and 20 subject MAT1-1-1 proteins (AGW27517−AGW27536 [14]) revealed truncation at both the N- and C-termini. The MATα_HMGbox domain (amino acids 51→225 in AGW27560) is highlighted in pink, whereas residues located outside this domain are shown in blue. Amino acid substitutions are highlighted in green. Hyphens indicate identical amino acids, whereas blank spaces denote unmatched sequence alignment gaps.

```
AEH27625 1    MANPINMIPNPQWNATDYEAIWKGLEAQVNPFSQILCLEGDFFRQLDDAAKLFIARKLME  60
AGW27538 1    ------------------------------------------------------------  60
AGW27541 1    ------------------------------------------------------------  60
AGW27539 1    ------------------------------------------------------------  60
AGW27556 1    ------------------------------------------------------------  60
AGW27547 1    ------------------------------------------------------------  60
AGW27546 1    ------------------------------------------------------------  60
AGW27545 1    ------------------------------------------------------------  60
AGW27542 1    ------------------------------------------------------------  60
AGW27549 1    ------------------------------------------------------------  60
AGW27550 1    ------------------------------------------------------------  60
AGW27551 1    ------------------------------------------------------------  60
AGW27544 1    ------------------------------------------------------------  60
AGW27540 1    ------------------------------------------------------------  60
AGW27537 1    ------------------------------------------------------------  60
AGW27553 1    ------------------------------------------------------------  60
AGW27543 1    ------------------------------------------------------------  60
AGW27554 1    ------------------------------------------------------------  60
AGW27555 1    ------------------------------------------------------------  60
AGW27548 1    ------------------------------------------------------------  60
AGW27552 1    ------------------------------------------------------------  60

AEH27625 61   HVQESVLYVNDGNGPDRVYLGAPRHFVVGGGMILQISGYAPYWIRRSVSKVVTATVLAPP  120
AGW27538 61   ------------------------------------------------------------  120
AGW27541 61   ------------------------------------------------------------  120
AGW27539 61   ------------------------------------------------------------  120
AGW27556 61   ------------------------------------------------------------  120
AGW27547 61   ------------------------------------------------------------  120
AGW27546 61   ------------------------------------------------------------  120
AGW27545 61   ------------------------------------------------------------  120
AGW27542 61   ------------------------------------------------------------  120
AGW27549 61   ------------------------------------------------------------  120
AGW27550 61   ------------------------------------------------------------  120
AGW27551 61   ------------------------------------------------------------  120
AGW27544 61   ------------------------------------------------------------  120
AGW27540 61   ------------------------------------------------------------  120
AGW27537 61   -----------------I------------------------------------------  120
AGW27553 61   ------------------------------------------------------------  120
AGW27543 61   ------------------------------------------------------------  120
AGW27554 61   ------------------------------------------------------------  120
AGW27555 61   ------------------------------------------------------------  120
AGW27548 61   ------------------------------------------------------------  120
AGW27552 61   ------------------------------------------------------------  120

AEH27625 121  SPKDIKIPRPPNAYILYRKERHHYVKDANPGITNNEISQILGKAWNMESNDVRQKYKDMS  180
AGW27538 121  ------------------------------------------------------------  180
AGW27541 121  ------------------------------------------------------------  180
AGW27539 121  ------------------------------------------------------------  180
AGW27556 121  -----------------------H------------------------------------  180
AGW27547 121  -----------------------H------------------------------------  180
AGW27546 121  -----------------------H------------------------------------  180
AGW27545 121  -----------------------H------------------------------------  180
AGW27542 121  -----------------------H------------------------------------  180
AGW27549 121  -----------------------H------------------------------------  180
AGW27550 121  -----------------------H------------------------------------  180
AGW27551 121  -----------------------H------------------------------------  180
AGW27544 121  -----------------------H------------------------------------  180
AGW27540 121  -----------------------H------------------------------------  180
AGW27537 121  -----------------------H------------------------------------  180
AGW27553 121  -----------------------H------------------------------X-----  180
AGW27543 121  -----------------------H------------------------------------  180
AGW27554 121  -----------------------H------------------------------------  180
AGW27555 121  -----------------------H-------------------------------       175
AGW27548 121  -----------------------H-------------------------------       175
AGW27552 121  -----------------------H------------------------------        174
```

```
AEH27625 181  QQVKQALLEKHPDYQYKPRRPCERRRRRRASPNQNPKQSTSRNAATRDAAISSEDTSTAT  240
AGW27538 181  ------------------------------------------------------------  240
AGW27541 181  ------------------------------------------------------------  240
AGW27539 181  ------------------------------------------------------------  240
AGW27556 181  ------------------------------------------------------------  240
AGW27547 181  ------------------------------------------------------------  240
AGW27546 181  ------------------------------------------------------------  240
AGW27545 181  ------------------------------------------------------------  240
AGW27542 181  ------------------------------------------------------------  240
AGW27549 181  ------------------------------------------------------------  240
AGW27550 181  ------------------------------------------------------------  240
AGW27551 181  ------------------------------------------------------------  240
AGW27544 181  ------------------------------------------------------------  240
AGW27540 181  ------------------------------------------------------------  240
AGW27537 181  ------------------------------------------------------------  240
AGW27553 181  ------------------------------------------------------------  240
AGW27543 181  -----                                                         185
AGW27554 181  ----T                                                         185
AGW27555 175
AGW27548 175
AGW27552 174

AEH27625 241  GDTNTANGF                                                     249
AGW27538 241  ---------                                                     249
AGW27541 241  ---------                                                     249
AGW27539 241  ---------                                                     249
AGW27556 241  ---------                                                     249
AGW27547 241  ---------                                                     249
AGW27546 241  ---------                                                     249
AGW27545 241  ---------                                                     249
AGW27542 241  ---------                                                     249
AGW27549 241  ---------                                                     249
AGW27550 241  ---------                                                     249
AGW27551 241  ---------                                                     249
AGW27544 241  ---------                                                     249
AGW27540 241  ---------                                                     249
AGW27537 241  ---------                                                     249
AGW27553 241  ---------                                                     248
AGW27543 185
AGW27554 185
AGW27555 175
AGW27548 175
AGW27552 174
```

Figure S2. Alignment of the sequences of the 20 studied MAT1-2-1 proteins (AGW27537−AGW27556; including 15 full-length and 5 truncated proteins [14]) derived from 20 *O. sinensis* strains and the reference query full-length MAT1-2-1 protein AEH27625 from the *H. sinensis* strain CS2 (AlphaFold code D7F2E9). Amino acid residues within the HMG-box_ROX1-like domain (residues 127→197 in AEH27625) are shown in pink, whereas residues located outside this domain are shown in blue. Amino acid substitutions are shown in green. Hyphens indicate identical amino acids, whereas blank spaces denote unmatched sequence alignment gaps.

Panel (A): Alignment of amino acid sequences of the MATα_HMGbox domains

```
AGW27560  42   ADVLTPVSTAAASRATRQTKEASCDRAKRPLNAFMAFRSYYLKLFPDVQQKTASGFLTT  100
AGW27517  1                          -------------------------------------  37
AGW27518  1                          -------------------------------------  37
AGW27519  1                          -------------------------------------  37
AGW27520  1                          -------------------------------------  37
AGW27521  1                          -------------------------------------  37
AGW27523  1                          -------------------------------------  37
AGW27524  1                          -------------------------------------  37
AGW27528  1                          -------------------------------------  37

AGW27560  101 LWHKDPFRNKWALIAKVYSFVRDQIGKDKVSLSYFMSLACPTMTIIEPAAYLNALGWCVQ  160
AGW27517  38  ------------------------------------------------------------  97
AGW27518  38  ------------------------------------------------------------  97
AGW27519  38  ------------------------------------------------------------  97
AGW27520  38  ------------------------------------------------------------  97
AGW27521  38  ------------------------------------------------------------  97
AGW27523  38  ------------------------------------------------------------  97
AGW27524  38  ------------------------------------------------------------  97
AGW27528  38  ------------------------------------------------------------  97

AGW27560  161 DDDAGSQKLFQDESSANLDQSSLLSAEYPSTEIELLSALVNIGYFPDHGADLVERMGSSH  220
AGW27517  98  ------------------------------------------------------------  157
AGW27518  98  ------------------------------------------------------------  157
AGW27519  98  ------------------------------------------------------------  157
AGW27520  98  ------------------------------------------------------------  157
AGW27521  98  ------------------------------------------------------------  157
AGW27523  98  ------------------------------------------------------------  157
AGW27524  98  ------------------------------------------------------------  157
AGW27528  98  ------------------------------------------------------------  157

AGW27560  221 SGIMAPRAANCTPP                                                234
AGW27517  158 --------------                                                171
AGW27518  158 --------------                                                171
AGW27519  158 --------------                                                171
AGW27520  158 --------------                                                171
AGW27521  158 --------------                                                171
AGW27523  158 --------------                                                171
AGW27524  158 --------------                                                171
AGW27528  158 --------------                                                171
```

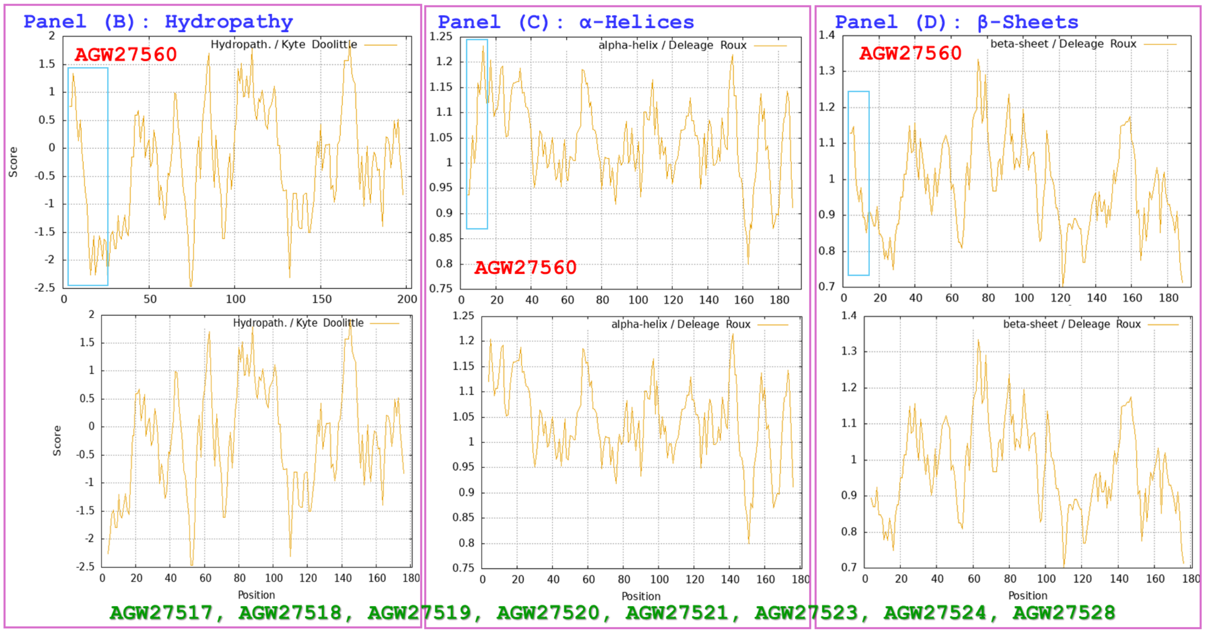

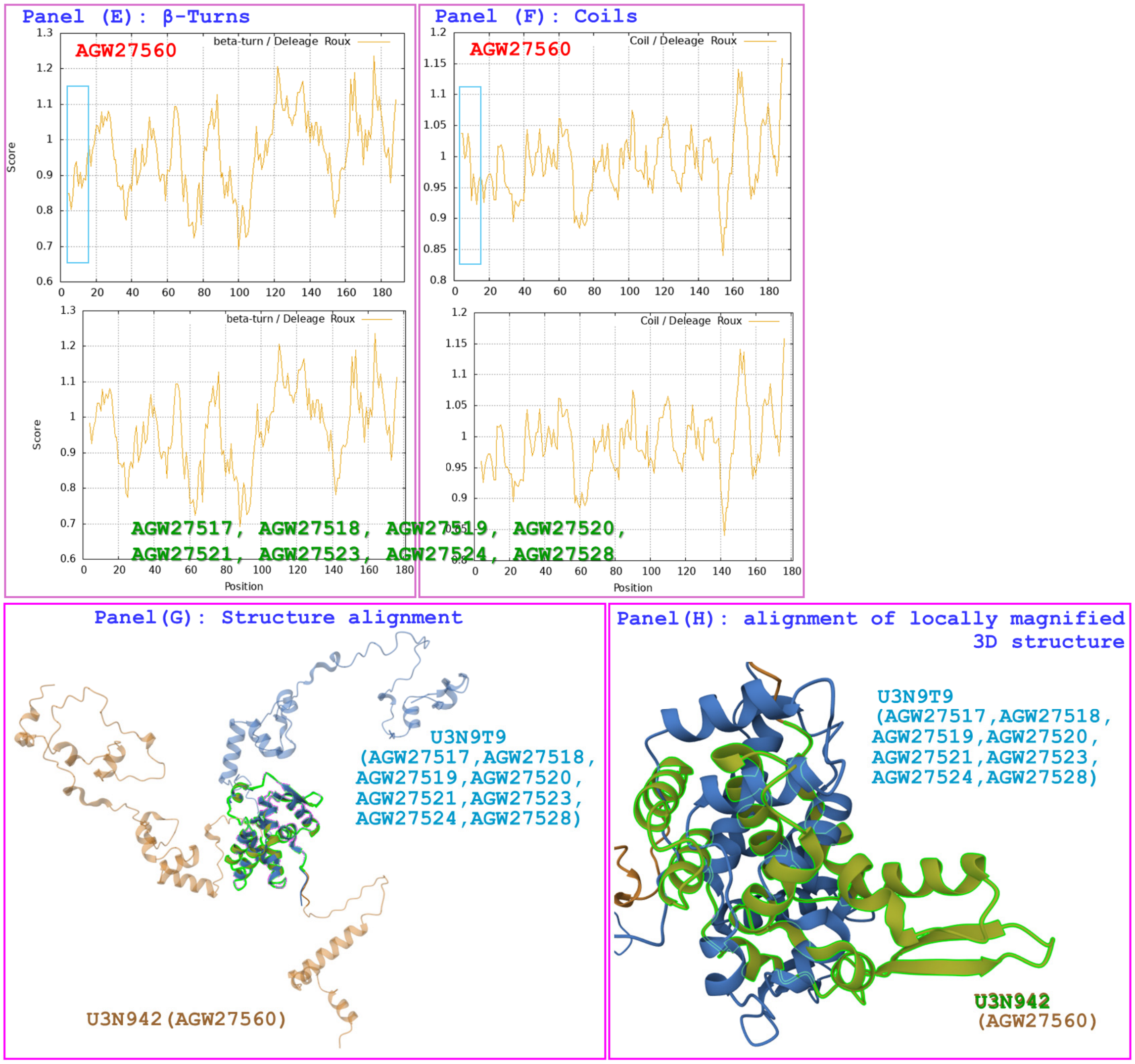

Figure S3. Correlations among changes in hydrophobicity and the primary, secondary, and tertiary structures of the MATα_HMGbox domains of MAT1-1-1 proteins. The reference protein AGW27560 (AlphaFold model U3N942) was derived from the *H. sinensis* strain CS68-2-1229, whereas the truncated MAT1-1-1 proteins (AlphaFold model U3N9T9) were derived from the *O. sinensis* strains CS6-251, CS36-1294, CS37-295, CS18-266, CS26-277, CS91-1291, CS34-291, and CS68-2-1229. Panel (A) presents alignments of the amino acid sequences of the MATα_HMGbox domains of the MAT1-1-1 proteins. Hyphens indicate identical amino acid residues, whereas blank spaces denote unmatched sequence alignment gaps. ExPASy ProtScale analyses illustrating changes in hydrophobicity and predicted 2D structural characteristics are shown in Panels (B)–(F), including hydropathy, α-helices, β-sheets, β-turns, and coils of the proteins. The open blue rectangles in the ExPASy plots denote the N-terminally truncated regions. Panel (G) shows pairwise structural superposition of AlphaFold-predicted 3D structures performed using the TM-align algorithm to compare the overall protein folds of the 2 tertiary structural models (U3N942 *vs*. U3N9T9) of proteins, with the 3 critical α-helix regions within the DNA-binding domain of the reference protein AGW27560 are highlighted with light green outlines. Panel (H) shows independent structural superpositions of the MATα_HMGbox domains using the SW 3D algorithm to optimize local structural alignment and enable clear visualization of regional structural differences, focusing on the 3 α-helices that form the hydrophobic core.

Panel (A): Alignment of amino acid sequences of the HMG-box_ROX1-like domains

```
AEH27625 118 APPSPKDIKIPRPPNAYILYRKERHHYVKDANPGITNNEISQILGKAWNMESNDVRQKYK  177
AGW27548 118 --------------------------H-------------------------------    175
AGW27555 118 --------------------------H-------------------------------    175
AGW27543 118 --------------------------H---------------------------------  177

AEH27625 178 DMSQQVKQALLEKHPDYQYKPRRPCERRR                                 206
AGW27548 175
AGW27555 175
AGW27543 178 --------                                                      185
```

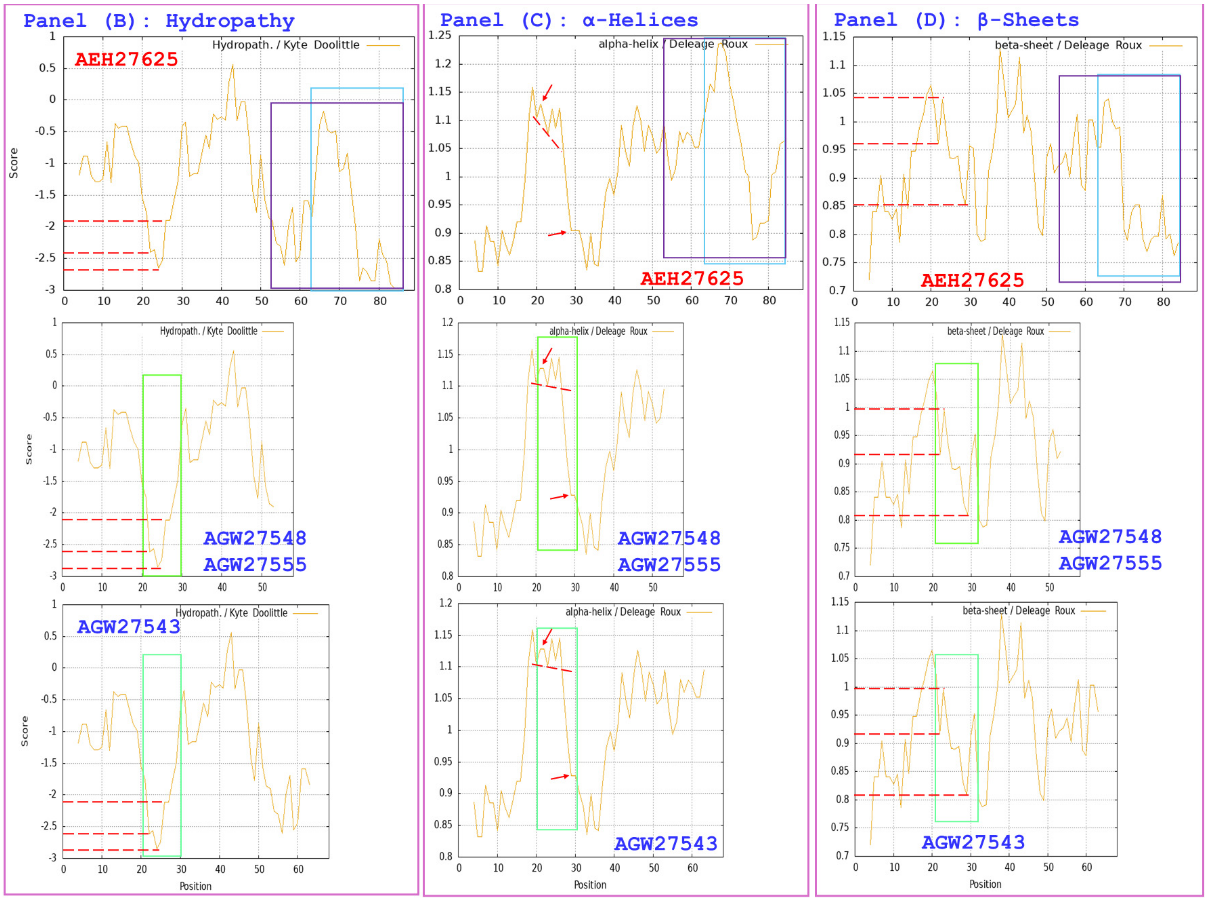

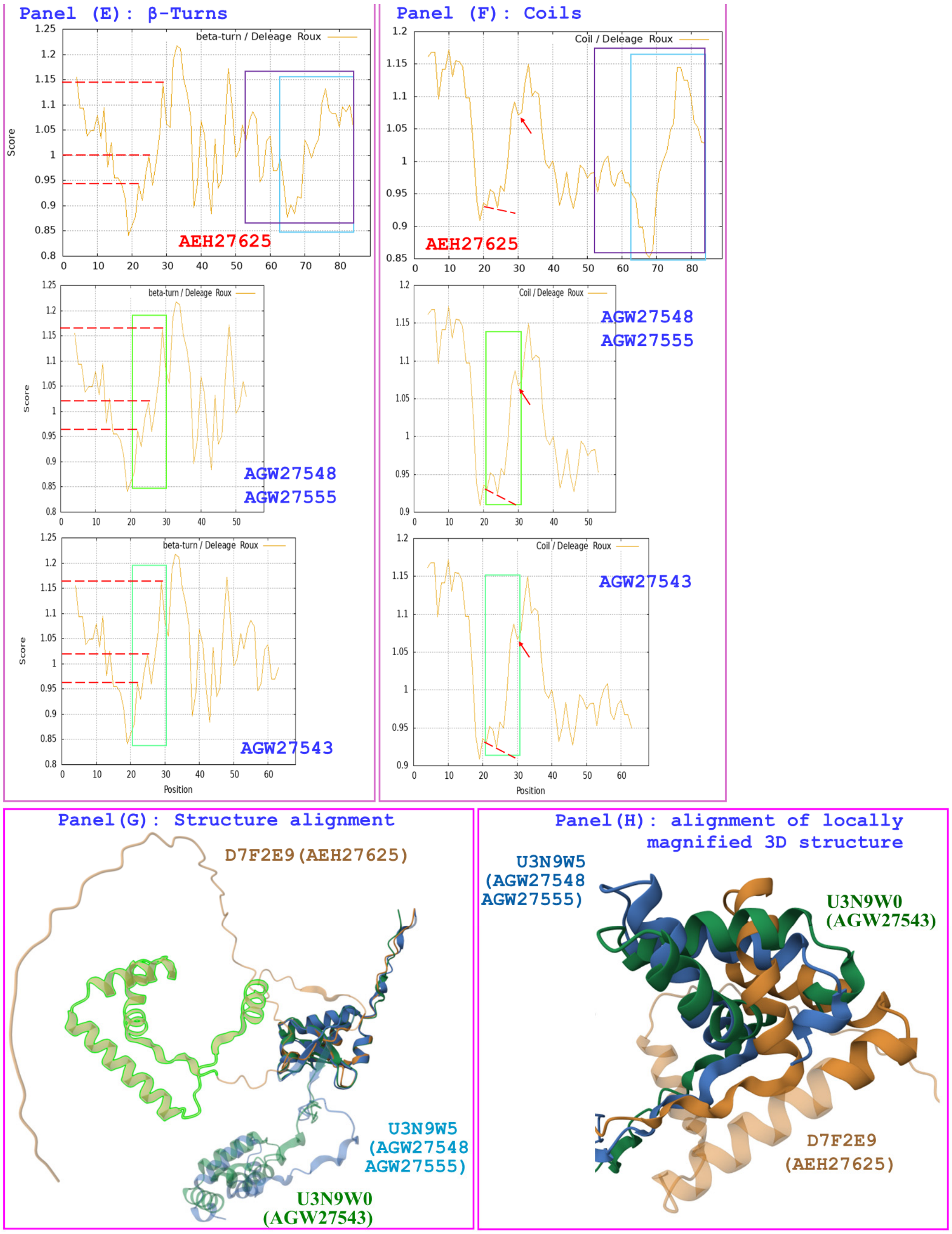

Figure S4. Correlations among changes in hydrophobicity and the primary, secondary, and tertiary structures of the HMG-box_ROX1-like domains of MAT1-2-1 proteins. The reference protein AEH27625 (AlphaFold model D7F2E9) was derived from the *H. sinensis* strain CS2, whereas the variant MAT1-2-1 proteins AGW27548 and AGW27555 (AlphaFold model U3N9W5), AGW27542 (AlphaFold model U3N6W6), and protein AGW27543 (AlphaFold model U3N9W0) were derived from the *O. sinensis* strains CS68-2-1229, CS71-1220, CS68-5-1216, and CS91-1291, respectively. Panel (A) presents alignments of the amino acid sequences of the HMG-box_ROX1-like domains of the MAT1-2-1 proteins. Amino acid substitutions are highlighted in green, hyphens indicate identical amino acid residues, and blank spaces denote unmatched sequence alignment gaps. ExPASy ProtScale analyses illustrating changes in the hydrophobicity and predicted secondary structural characteristics of the proteins are shown in Panels (B)–(F), including hydropathy, α-helices, β-sheets, β-turns, and coils, respectively. The open purple and blue rectangles highlight the C-terminally truncated regions in the ExPASy plots, whereas the open green rectangles with red dashed auxiliary lines or directional arrows highlight alterations in topological configuration and waveform patterns. Panel (G) shows pairwise structural superposition of AlphaFold-predicted 3D structures performed using the TM-align algorithm to compare the overall protein folds of the 3 tertiary structural models

(D7F2E9 *vs*. U3N9W5/U3N9W0) of proteins, with the 3 critical α-helix regions within the DNA-binding domain of the reference protein AEH27625 are highlighted with light green outlines. Panel (H) shows independent structural superpositions of the HMG-box_ROX1-like domains using the SW 3D algorithm to optimize local structural alignment and enable clear visualization of regional structural differences, focusing on the 3 α-helices that form the hydrophobic core.

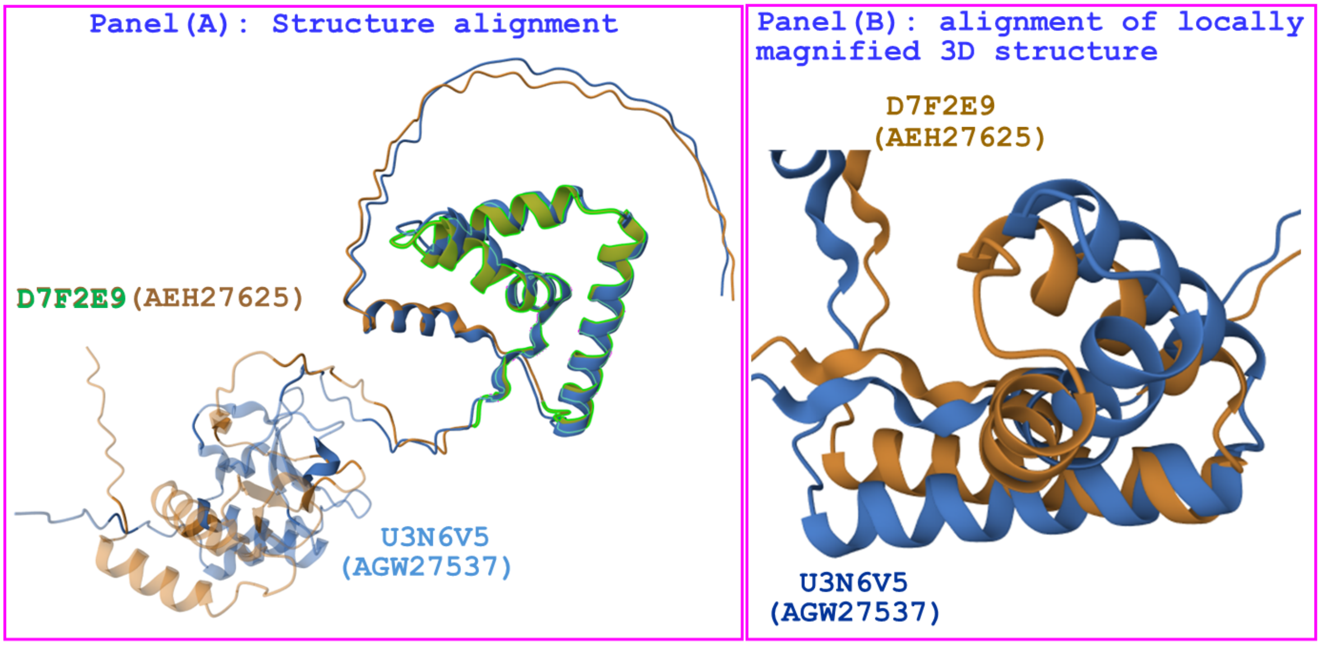

Figure S5. Structures superposition of the MAT1-2-1 proteins. The reference protein AEH27625 (AlphaFold model D7F2E9) was derived from the *H. sinensis* strain CS2, whereas the variant MAT1-2-1 protein AGW27537 (AlphaFold code U3N6V5) was derived from the *O. sinensis* strain CS6-251. Panel (A) shows pairwise structural superposition of AlphaFold-predicted 3D structures performed using the TM-align algorithm to compare the overall protein folds of the 2 tertiary structural models (D7F2E9 *vs*. U3N6V5) of proteins, with the 3 critical α-helix regions within the DNA-binding domain of the reference protein AEH27625 are highlighted with light green outlines. Panel (B) shows independent structural superpositions of the HMG-box_ROX1-like domains using the SW 3D algorithm to optimize local structural alignment and enable clear visualization of regional structural differences, focusing on the 3 α-helices that form the hydrophobic core.

Panel (A): Alignment of the amino acid sequences of the MATα_HMGbox domains

```
AGW27560  42    ADVLTPVSTAAASRATRQTKEASCDRAKRPLNAFMAFRSYYLKLFPDVQQKTASGFLTT  100
AGW27532  1                                                            ---  3
AGW27533  1                                                            ---  3
AGW27535  1                                                            ---  3

AGW27560  101  LWHKDPFRNKWALIAKVYSFVRDQIGKDKVSLSYFMSLACPTMTIIEPAAYLNALGWCVQ  160
AGW27532  4    ------------------------------------------------------------  63
AGW27533  4    ------------------------------------------------------------  63
AGW27535  4    ------------------------------------------------------------  63

AGW27560  161  DDDAGSQKLFQDESSANLDQSSLLSAEYPSTEIELLSALVNIGYFPDHGADLVERMGSSH  220
AGW27532  64   ------------------------------------------------------------  123
AGW27533  64   ------------------------------------------------------------  123
AGW27535  64   ------------------------------------------------------------  123

AGW27560  221  SGIMAPRAANCTPP                                                234
AGW27532  124  --------------                                                137
AGW27533  124  --------------                                                137
AGW27535  124  --------------                                                137
```

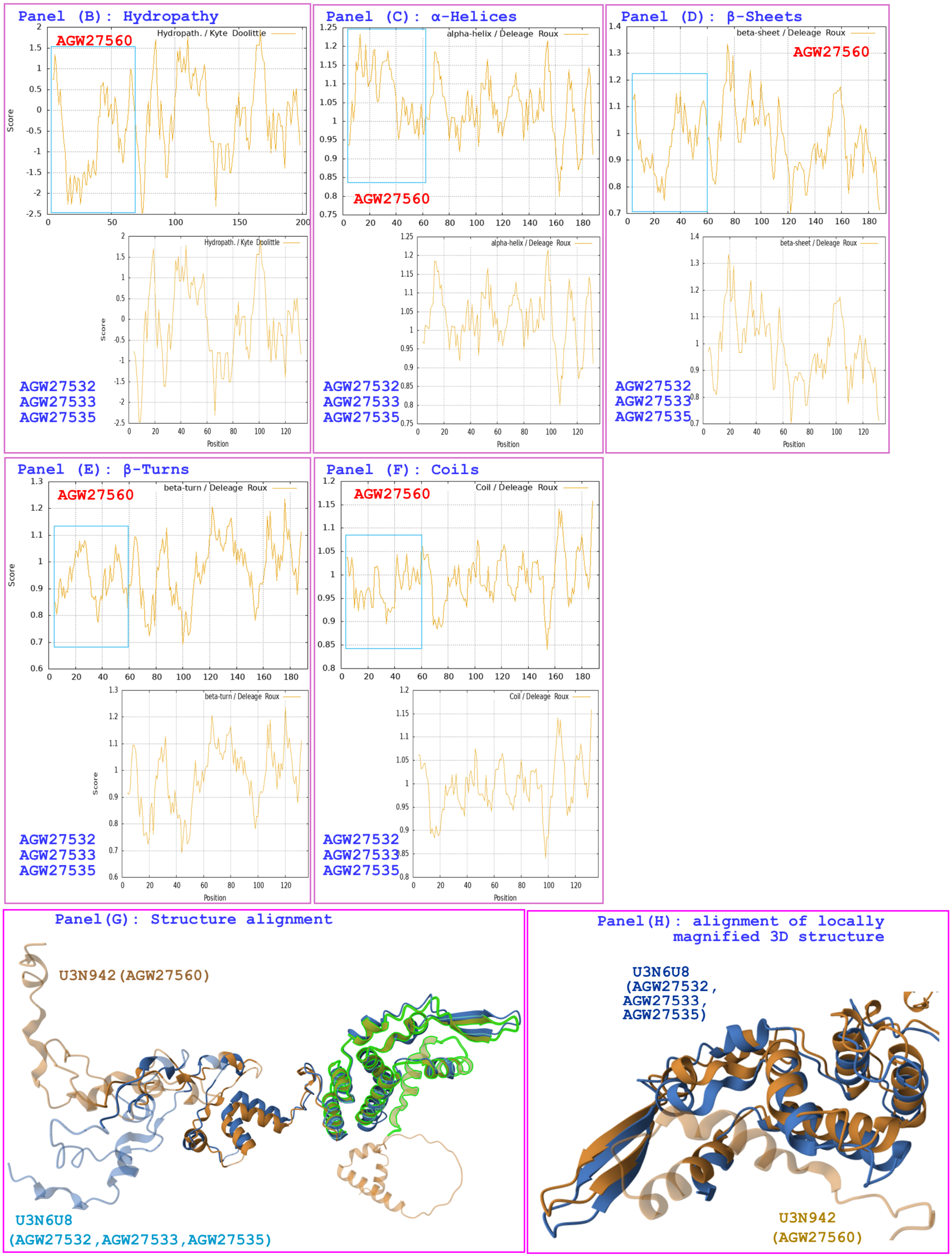

Figure S6. Correlations among alterations in hydrophobicity and the primary, secondary, and tertiary structures of the MATα_HMGbox domains of the MAT1-1-1 proteins. The reference protein AGW27560 (AlphaFold model U3N942) was derived from the *H. sinensis* strain CS68-2-1229, whereas the variant proteins AGW27532, AGW27533, and AGW27535 (AlphaFold model U3N6U8) were derived from the *O. sinensis* strains CS68-5-1216,

CS71-1218, and CS71-1220, respectively. Panel (A) presents alignments of the amino acid sequences of the MATα_HMGbox domains of the MAT1-1-1 proteins. Hyphens indicate identical amino acid residues, whereas blank spaces denote unmatched sequence alignment gaps. ExPASy ProtScale analyses illustrating changes in hydrophobicity and predicted secondary structure characteristics are shown in Panels (B)–(F), including hydropathy, α-helices, β-sheets, β-turns, and coils of the protein, respectively. The open blue rectangles in the ExPASy plots highlight the N-terminally truncated regions. Panel (G) shows pairwise structural superposition of AlphaFold-predicted 3D structures performed using the TM-align algorithm to compare the overall protein folds of the 2 tertiary structural models (U3N942 *vs*. U3N6U8) of proteins, with the 3 critical α-helix regions within the DNA-binding domain of the reference protein AGW27560 are highlighted with light green outlines. Panel (H) shows independent structural superpositions of the MATα_HMGbox domains using the SW 3D algorithm to optimize local structural alignment and enable clear visualization of regional structural differences, focusing on the 3 α-helices that form the hydrophobic core.

```
Panel (A):  Alignment of amino acid sequences of the HMG-box_ROX1-like domains
AEH27625 118 APPSPKDIKIPRPPNAYILYRKERHHYVKDANPGITNNEISQILGKAWNMESNDVRQKYK  177
AGW27555 118 --------------------------H-------------------------------    175
AGW27548 118 --------------------------H-------------------------------    175
AGW27552 118 --------------------------H------------------------------     174
AGW27553 118 --------------------------H------------------------------X--  177

AEH27625 178 DMSQQVKQALLEKHPDYQYKPRRPCERRR                                206
AGW27555 175
AGW27548 175
AGW27552 174
AGW27553 178 -----------------------------                                206
```

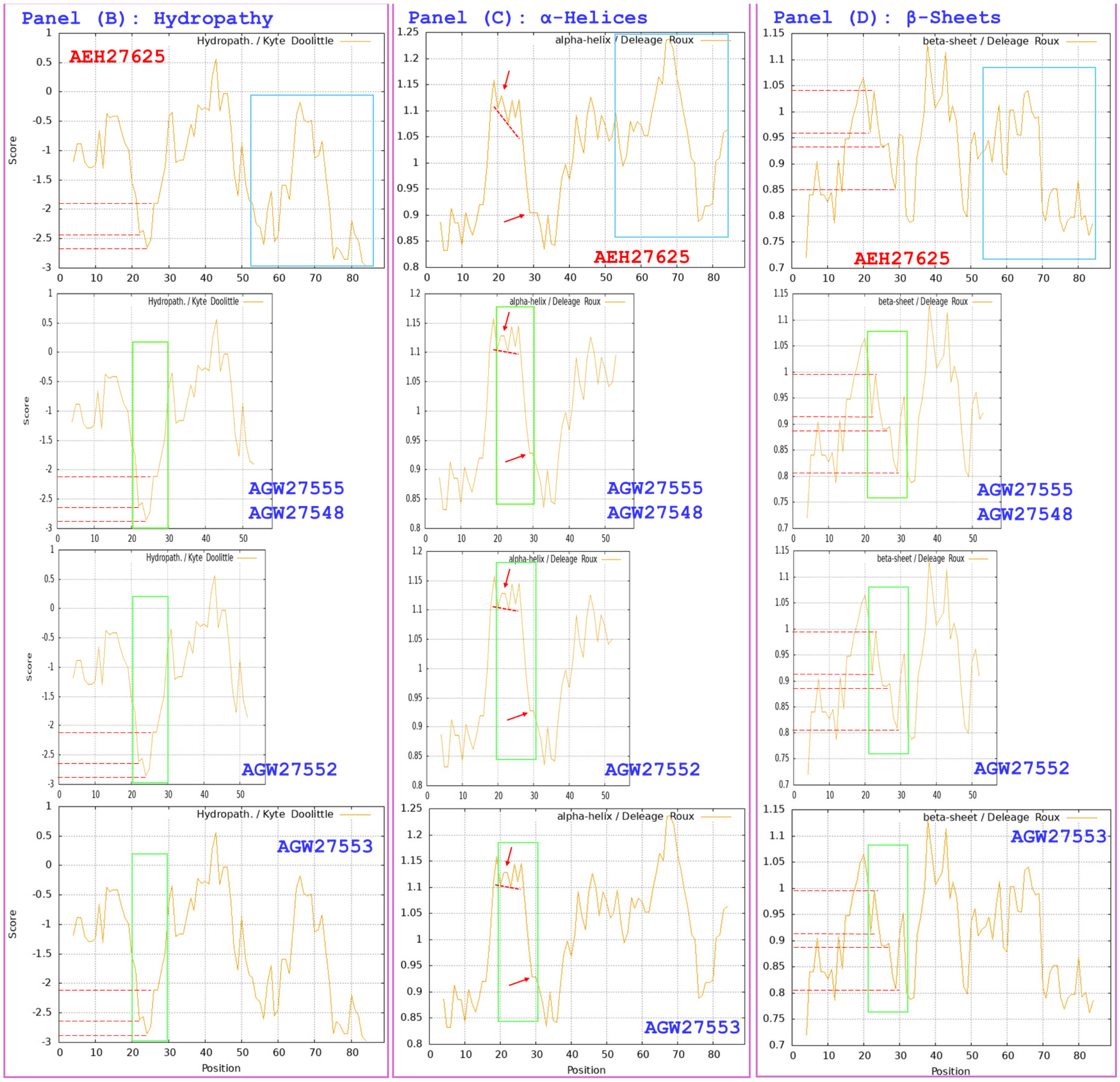
Panel (B): Hydropathy
Panel (C): α-Helices
Panel (D): β-Sheets
Hydropath. / Kyte Doolittle
alpha-helix / Deleage Roux
beta-sheet / Deleage Roux
AEH27625
AGW27555
AGW27548
AGW27552
AGW27553
Score
Position

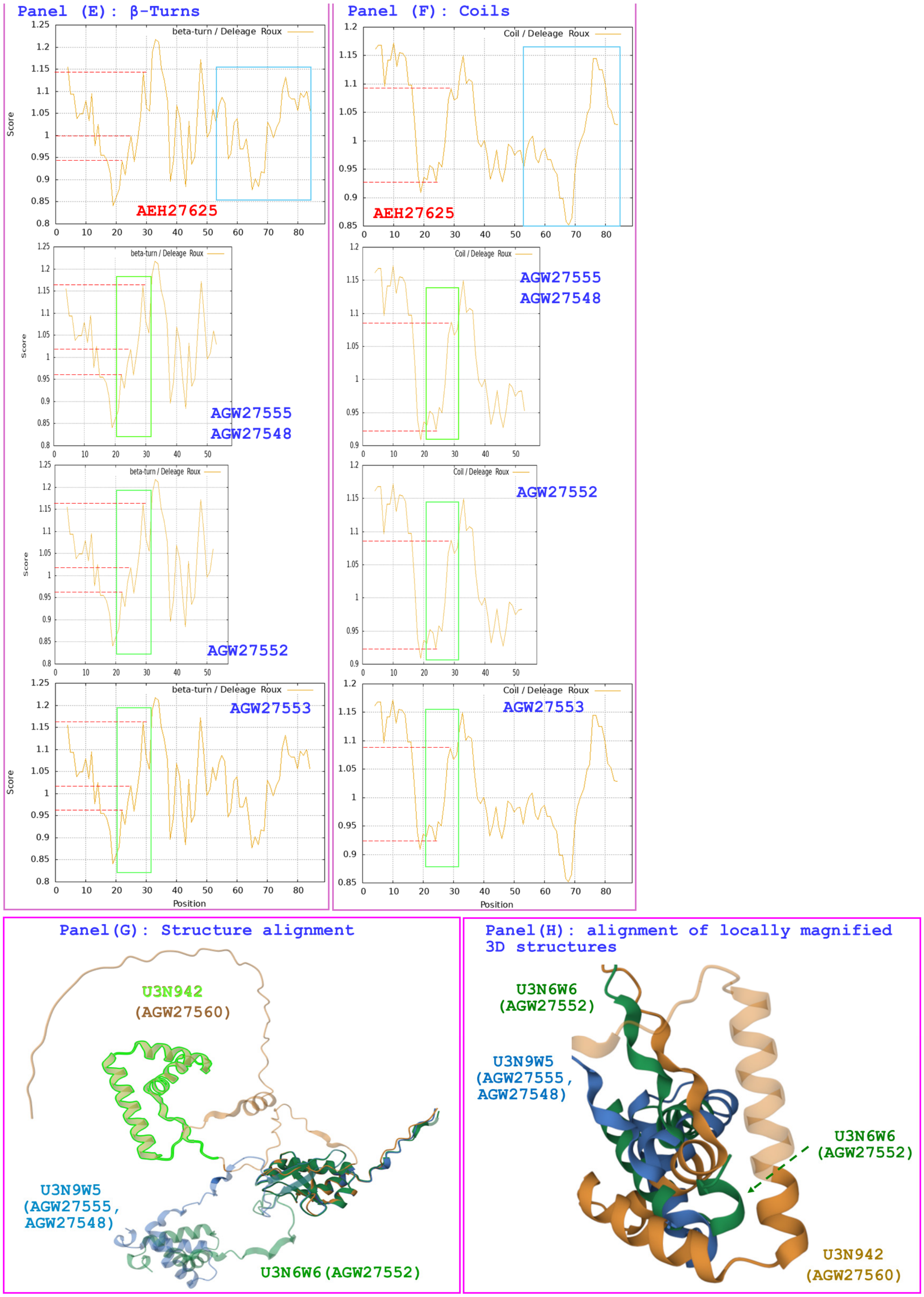

Figure S7. Correlations among alterations in hydrophobicity and the primary, secondary, and tertiary structures of the HMG-box_ROX1-like domains of the MAT1-2-1 proteins. The reference protein AEH27625 (AlphaFold model D7F2E9) was derived from the *H. sinensis* strain CS2, whereas the variant MAT1-2-1 proteins AGW27552 (AlphaFold model U3N6W6), AGW27553 (AlphaFold model U3N9X0), and AGW27555 and AGW27548 (AlphaFold model U3N9W5) were derived from the *O. sinensis* strains CS68-5-1216, CS71-1218, CS71-1220, and CS68-2-1229, respectively. Panel (A) presents alignments of the amino acid sequences of the HMG-box_ROX1-like

domains of the MAT1-2-1 proteins. Amino acid substitutions are highlighted in green, hyphens indicate identical amino acid residues, and blank spaces denote unmatched sequence alignment gaps. ExPASy ProtScale analyses illustrating changes in the hydrophobicity and predicted secondary structural characteristics of the proteins are shown in Panels (B)–(F), including hydropathy, α-helices, β-sheets, β-turns, and coils. The open purple and blue rectangles highlight the C-terminally truncated regions in the ExPASy plots, whereas the open green rectangles with red dashed auxiliary lines or directional arrows highlight the changes in topological configuration and waveform patterns. Panel (G) shows pairwise structural superposition of AlphaFold-predicted 3D models performed using the TM-align algorithm to compare the overall protein folds of the 4 tertiary structural models (D7F2E9 *vs.* U3N6W6/U3N9X0/U3N9W5), with the 3 critical α-helix regions within the DNA-binding domain of the reference protein AEH27625 are highlighted with green-outlines. Panel (H) shows independent structural superpositions of the HMG-box_ROX1-like domains using the SW 3D algorithm to optimize local structural alignment and enable clear visualization of regional structural differences, focusing on the 3 α-helices that form the hydrophobic core.

Panel (A): Alignment of the amino acid sequences of the MATα_HMGbox domains

```
AGW27560  42   ADVLTPVSTAAASRATRQTKEASCDRAKRPLNAFMAFRSYYLKLFPDVQQKTASGFLTT  100
AGW27529  1                                                           V---  4
AGW27530  1                                                           V---  4

AGW27560  101  LWHKDPFRNKWALIAKVYSFVRDQIGKDKVSLSYFMSLACPTMTIIEPAAYLNALGWCVQ  160
AGW27529  5    ------------------------------------------------------------  64
AGW27530  5    ------------------------------------------------------------  64

AGW27560  161  DDDAGSQKLFQDESSANLDQSSLLSAEYPSTEIELLSALVNIGYFPDHGADLVERMGSSH  220
AGW27529  65   ------------------------------------------------------------  124
AGW27530  65   ------------------------------------------------------------  124

AGW27560  221  SGIMAPRAANCTPP                                                234
AGW27529  125  --------------                                                138
AGW27530  125  --------------                                                138
```

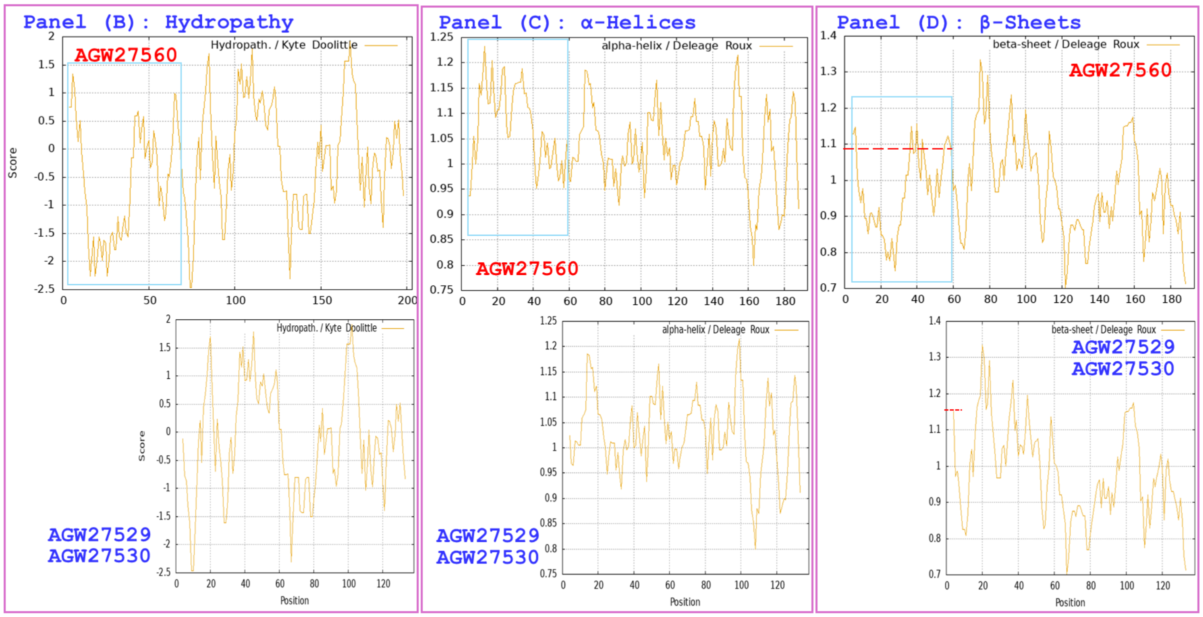

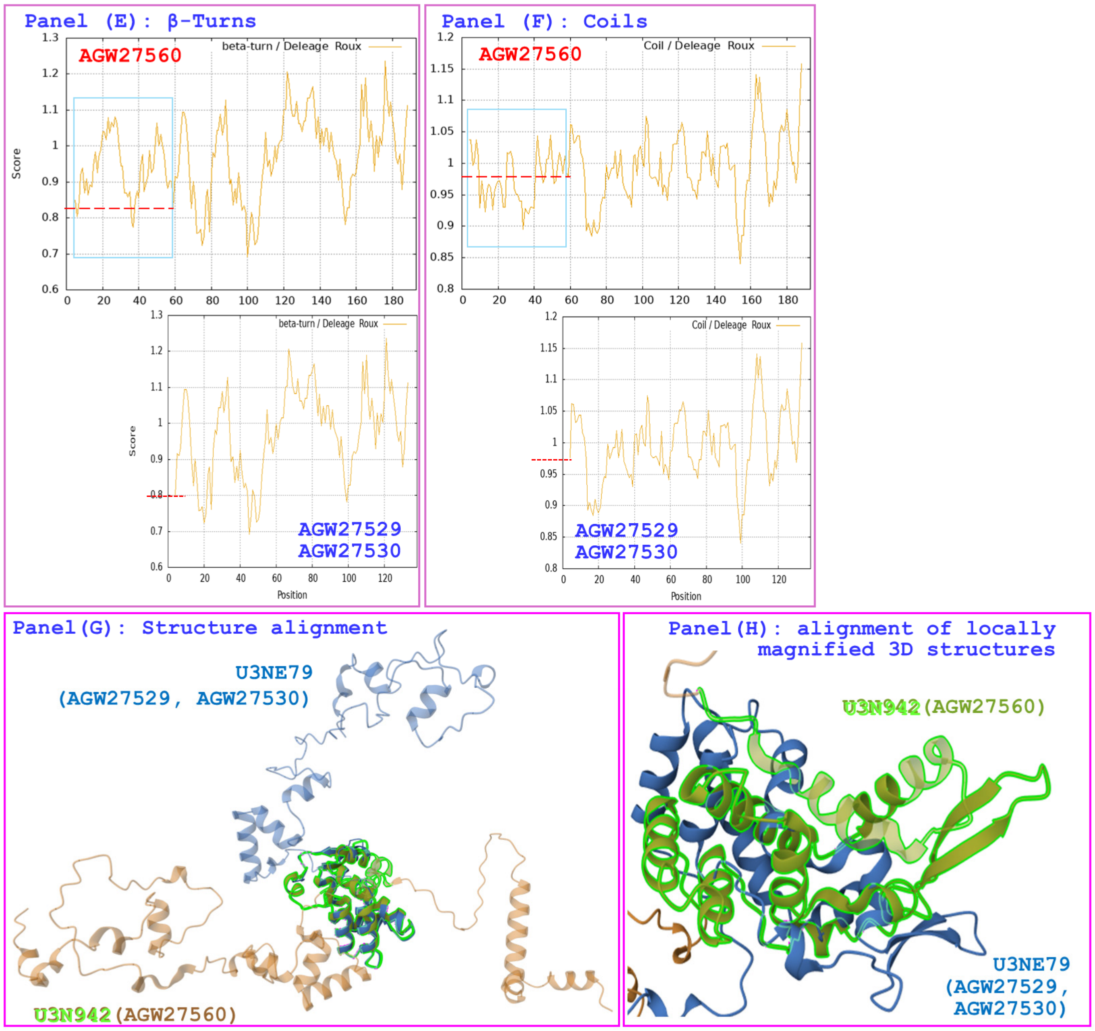

Figure S8. Correlations among alterations in hydrophobicity and the primary, secondary, and tertiary structures of the MATα_HMGbox domains of the MAT1-1-1 proteins. The reference protein AGW27560 (AlphaFold model U3N942) was derived from the *H. sinensis* strain CS68-2-1229, whereas the variant proteins AGW27529 and AGW27530 (AlphaFold model U3NE79) were derived from the *O. sinensis* strains CS70-1208 and CS70-1211, respectively. Panel (A) presents alignments of the amino acid sequences of the MATα_HMGbox domains of the MAT1-1-1 proteins. Amino acid substitutions are highlighted in green, hyphens indicate identical amino acid residues, and blank spaces denote unmatched sequence alignment gaps. ExPASy ProtScale analyses illustrating changes in hydrophobicity and predicted secondary structure characteristics are shown in Panels (B)–(F), including hydropathy, α-helices, β-sheets, β-turns, and coils of the protein. The open blue rectangles in the ExPASy plots highlight the N-terminally truncated regions, whereas the red dashed auxiliary lines highlight the changes in topological configuration and waveform patterns. Panel (G) shows pairwise structural superposition of the AlphaFold-predicted 3D structures performed using the TM-align algorithm to compare the overall protein folds of the 2 tertiary structural models (U3N942 *vs*. U3NE79) of proteins, with the 3 critical α-helix regions within the DNA-binding domain of the reference protein AEH27625 are highlighted with green-outlines. Panel (H) shows independent structural superpositions of the MATα_HMGbox domains using the SW 3D algorithm to optimize local structural alignment and enable clear visualization of regional structural differences, focusing on the 3 α-helices that form the hydrophobic core.

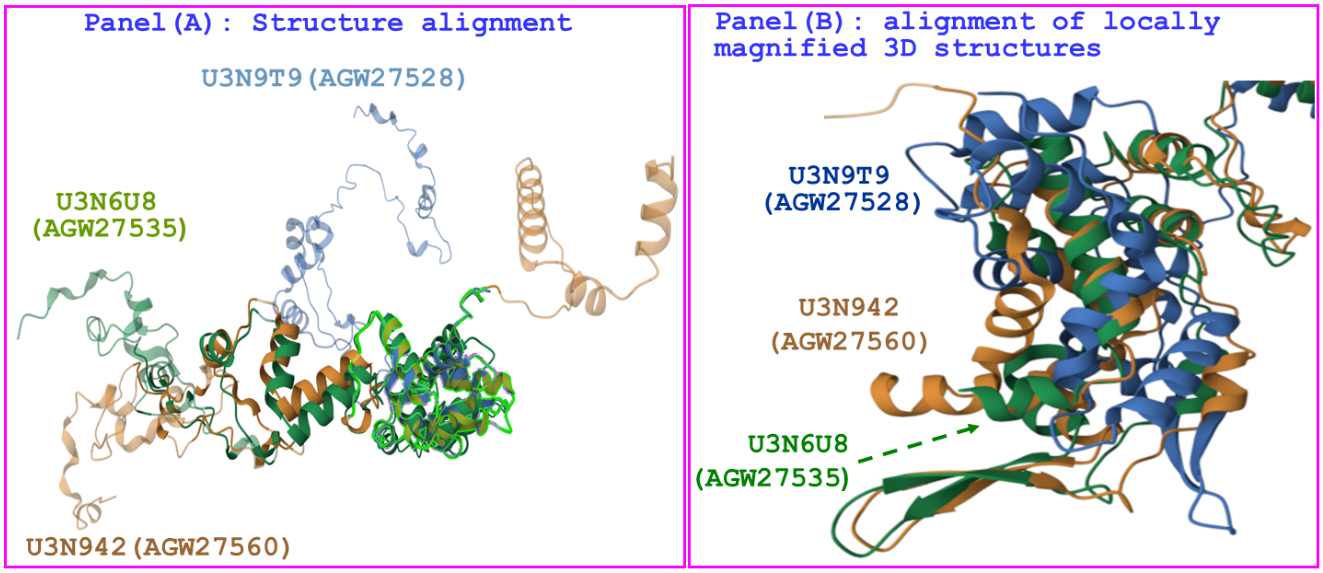

Figure S9. Structures superposition of the MAT1-1-1 proteins. The reference protein AGW27560 (AlphaFold model U3N942) was derived from the *H. sinensis* strain CS68-2-1229, whereas the variant MAT1-1-1 proteins AGW27528 (AlphaFold code U3N9T9) and AGW27535 (AlphaFold code U3N6U8) were derived from the *O. sinensis* strains CS68-2-1229 and CS71-1220. Panel (A) shows pairwise structural superposition of AlphaFold-predicted 3D structures performed using the TM-align algorithm to compare the overall protein folds of the 3 tertiary structural models (U3N942 *vs*. U3N9T9/U3N6U8), with the 3 critical α-helix regions within the DNA-binding domain of the reference protein AGW27560 are highlighted with green-outlines. Panel (B) shows independent structural superpositions of the MATα_HMGbox domains using the SW 3D algorithm to optimize local structural alignment and enable clear visualization of regional structural differences, focusing on the 3 α-helices that form the hydrophobic core.

Panel (A): Alignment of the amino acid sequences of the MATα_HMGbox domains

```
AGW27560  42   ADVLTPVSTAAASRATRQTKEASCDRAKRPLNAFMAFRSYYLKLFPDVQQKTASGFLTT  100
AGW27522  1                         -------------------------------------  37
AGW27525  1                            ----------------------------------  34
AGW27526  1                         -SSFT--------------------------------  37
AGW27527  1                                        -----------------------  23
AGW27536  1                                                         E-- --  5

AGW27560  101  LWHKDPFRNKWALIAKVYSFVRDQIGKDKVSLSYFMSLACPTMTIIEPAAYLNALGWCVQ  160
AGW27522  38   ------------------------------------------------------------  97
AGW27525  35   ------------------------------------------------------------  94
AGW27526  38   ------------------------------------------------------------  97
AGW27527  24   ------------------------------------------------------------  83
AGW27536  6    ------------------------------------------------------------  65

AGW27560  161  DDDAGSQKLFQDESSANLDQSSLLSAEYPSTEIELLSALVNIGYFPDHGADLVERMGSSH  220
AGW27522  98   ------------------------------------------------------------  157
AGW27525  95   ------------------------------------------------------------  154
AGW27526  98   ------------------------------------------------------------  157
AGW27527  84   ------------------------------------------------------------  143
AGW27536  66   ------------------------------------------------------------  125

AGW27560  221  SGIMAPRAANCTPP                                                234
AGW27522  158  --------------                                                171
AGW27525  155  --------------                                                168
AGW27526  158  --------------                                                171
AGW27527  144  --------------                                                157
AGW27536  126  --------------                                                139
```

Panel (B): Hydropathy

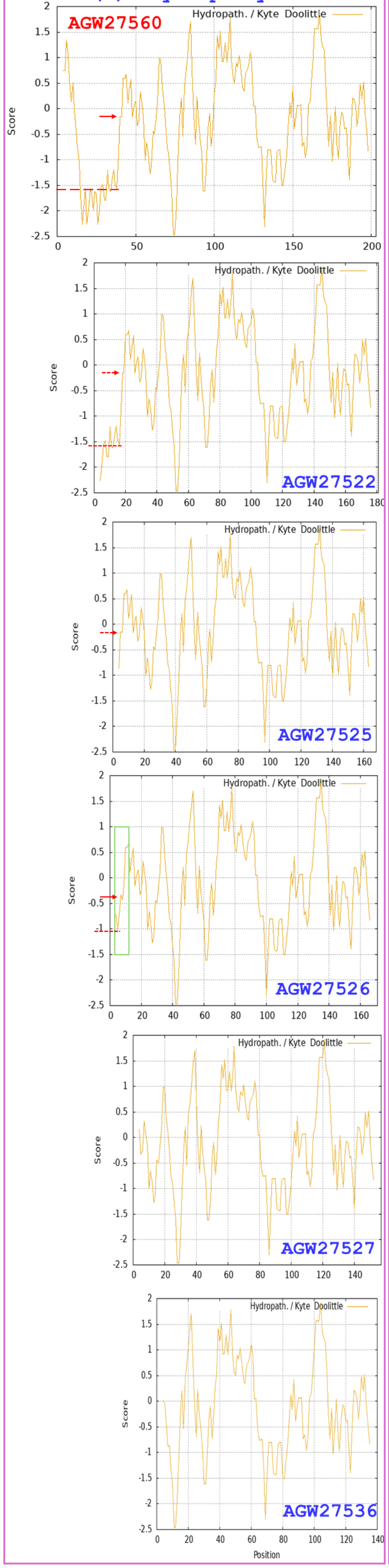

Panel (C): α-Helices

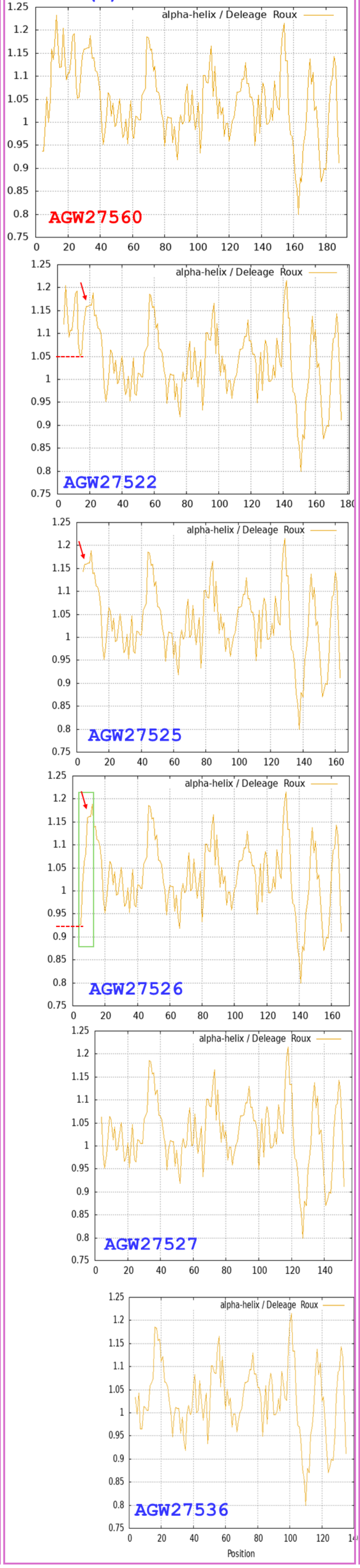

Panel (D): β-Sheets

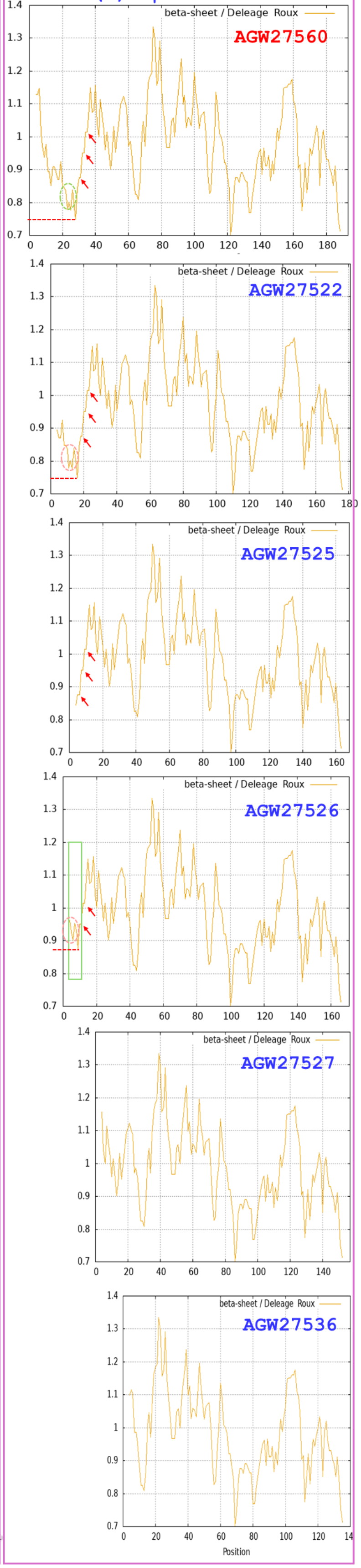

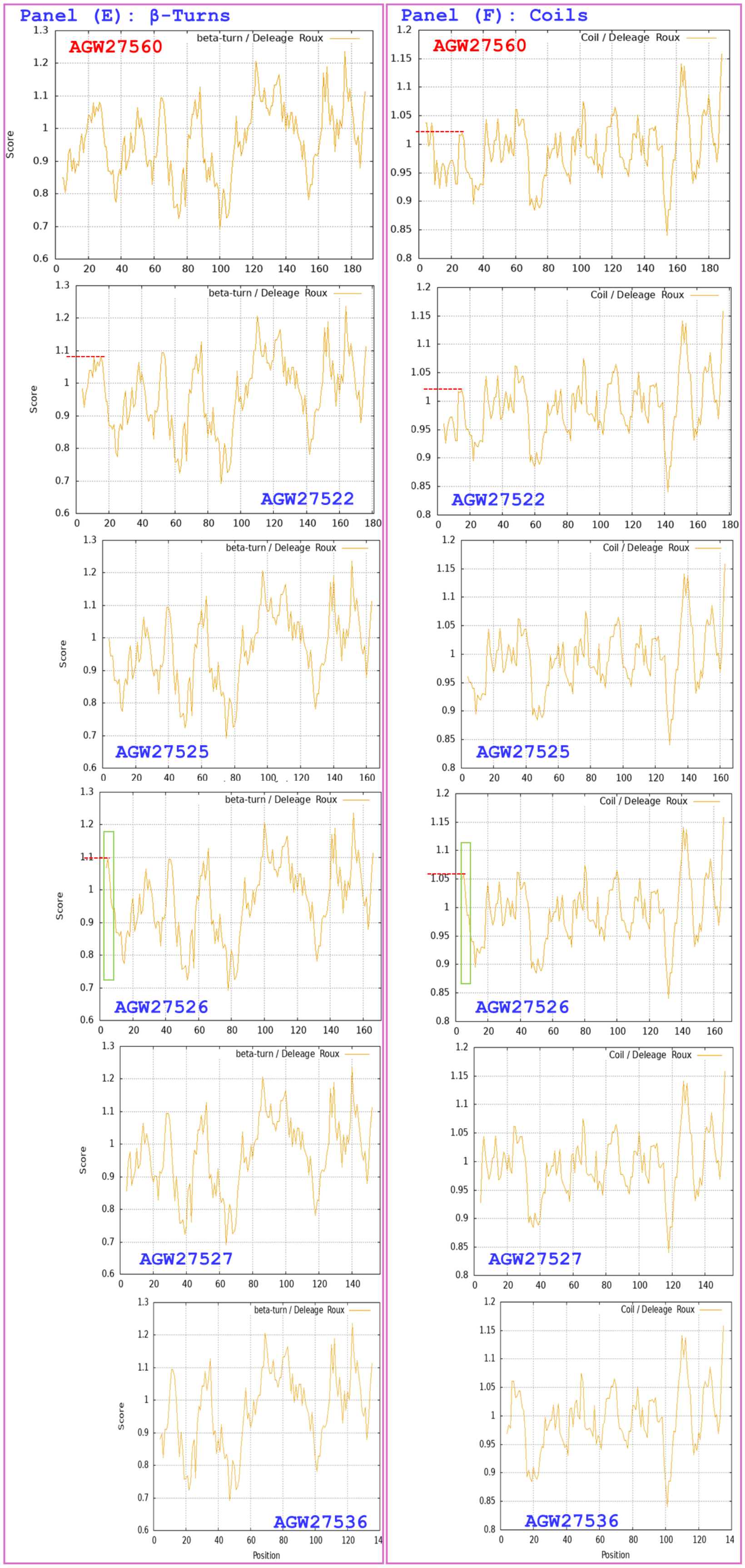
Panel (E): β-Turns
Panel (F): Coils
AGW27560
AGW27522
AGW27525
AGW27526
AGW27527
AGW27536
beta-turn / Deleage Roux
Coil / Deleage Roux
Score
Position

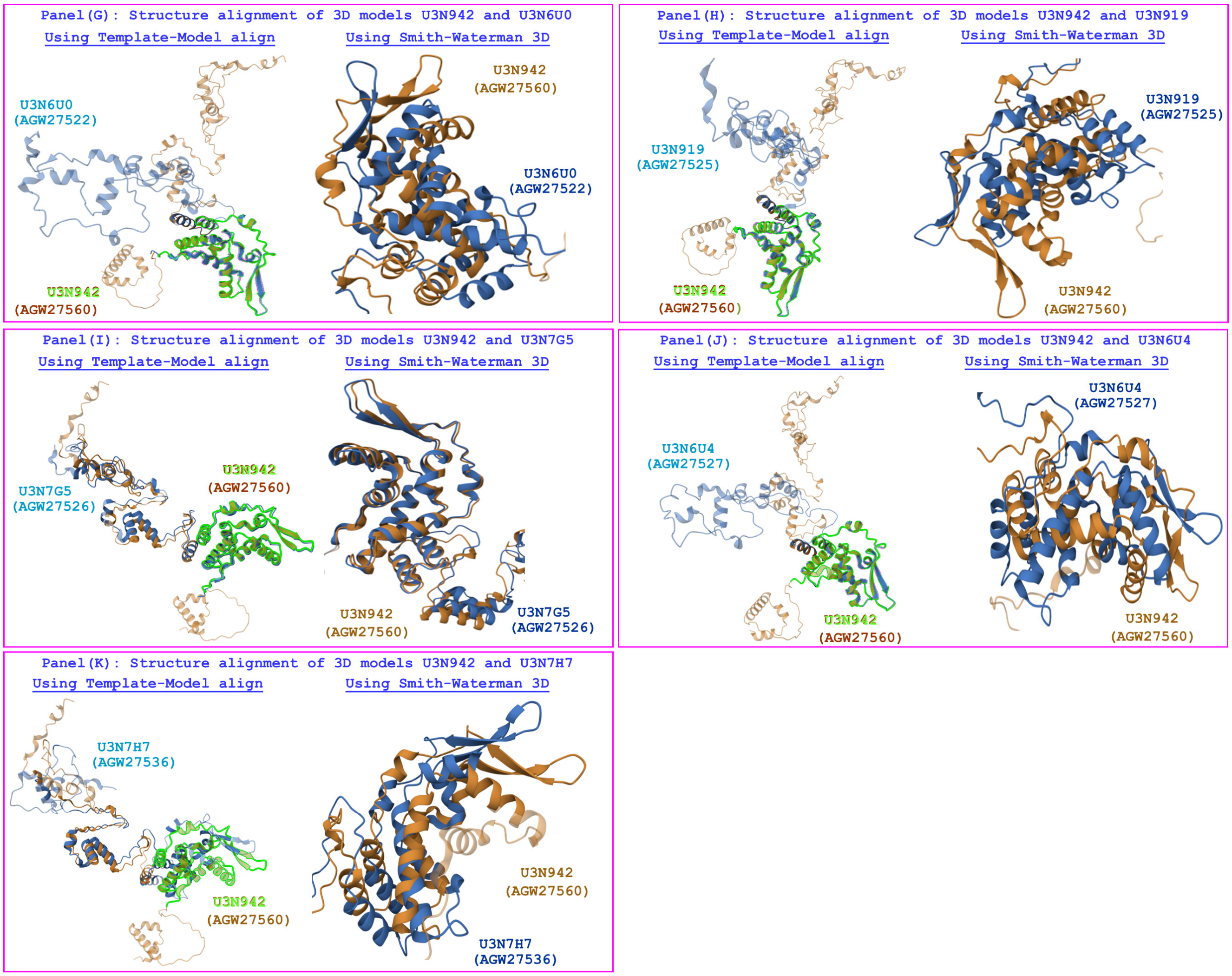

Figure S10. Correlations among alterations in hydrophobicity and the primary, secondary, and tertiary structures of the MATα_HMGbox domains of the MAT1-1-1 proteins. The reference protein AGW27560 (AlphaFold model U3N942) was derived from the *H. sinensis* strain CS68-2-1229, whereas the variant proteins AGW27522 (AlphaFold model U3N6U0), AGW27525 (AlphaFold model U3N919), AGW27526 (AlphaFold model U3N7G5), AGW27527 (AlphaFold model U3N6U4), and AGW27536 (AlphaFold model U3N7H7) were derived from the *O. sinensis* strains CS560-961, CS76-1284, CS561-964, CS25-273, and CS68-2-1228, respectively. Panel (A) presents alignments of the amino acid sequences of the MATα_HMGbox domains of the MAT1-1-1 proteins. Amino acid substitutions are highlighted in green, hyphens indicate identical amino acid residues, and blank spaces denote unmatched sequence alignment gaps. ExPASy ProtScale analyses illustrating changes in hydrophobicity and predicted secondary structural characteristics are shown in Panels (B)–(F), including hydropathy, α-helices, β-sheets, β-turns, and coils of the proteins, respectively. The open green rectangles with red dashed auxiliary lines and directory arrows highlight the changes in topological configuration and waveform patterns. Panels (G)–(K) present pairwise structural superpositions of the AlphaFold-predicted 3D structural models: (i) the left side of each panel shows structural overlays generated using the TM-align algorithm to compare the overall protein folds, and (ii) the right side of each panel shows independent structural superpositions of the MATα_HMGbox domains using the SW 3D algorithm to optimize local structural alignment and enable clear visualization of regional structural differences, focusing on the 3 α-helices that form the hydrophobic core. The AlphaFold-predicted 3D models were superposed pairwise by comparing the reference model U3N942 with the variant models U3N6U0, U3N919, U3N7G5, U3N6U4, and U3N7H7 in Panels (G)–(K), respectively.

Table S1. The *O. sinensis* strains, GenBank accession numbers for the ITS nucleic acid sequences corresponding to the GenBank accession numbers for the MAT1-1-1 and MAT1-2-1 proteins, and percent similarities *vs*. GC-biased Genotypes #1–3, #7–10, and #12 of *O. sinensis*.

***O. sinensis* strain**	**ITS1-5.8S-ITS2**	**Percent similarity between the sequences of ITS and the reference GC-biased Genotypes of *O. sinensis***							
		**#1**	**#2**	**#3**	**#7**	**#8**	**#9**	**#10**	**#12**
CS6-251	JQ900163	**98.2%**	**97.2%**	93.6%	93.2%	88.6%	93.9%	81.8%	93.1%
	JQ900180	87.3%	86.9%	85.9%	84.3%	83.2%	85.1%	76.1%	83.5%
CS34-291	JQ900162	**100%**	**97.2%**	95.3%	95.0%	89.5%	95.2%	83.1%	94.4%
	JQ900179	87.3%	86.9%	85.9%	84.3%	83.2%	85.1%	76.1%	83.5%
CS70-1208	JQ900146	**100%**	**97.2%**	95.3%	95.0%	89.5%	95.2%	83.1%	94.4%
	JQ900172	87.1%	86.3%	85.6%	84.3%	83.2%	85.3%	76.7%	83.7%
CS71-1220	JQ900152	**100%**	**97.2%**	95.3%	95.0%	89.5%	95.2%	83.1%	94.4%
	JQ900175	87.1%	86.3%	85.6%	84.3%	83.2%	85.3%	76.7%	83.7%
CS68-2-1228	JQ900157	**100%**	**97.2%**	95.3%	95.0%	89.5%	95.2%	83.1%	94.4%
	JQ900178	87.1%	86.3%	85.6%	84.3%	83.2%	85.3%	76.7%	83.7%
CS76-1284	JQ900159	**100%**	**97.2%**	95.3%	95.0%	89.5%	95.2%	83.1%	94.4%
CS68-2-1229	JQ900158	**100%**	**97.2%**	95.3%	95.0%	89.5%	95.2%	83.1%	94.4%
CS71-1218	JQ900150	**100%**	**97.2%**	95.3%	95.0%	89.5%	95.2%	83.1%	94.4%
CS71-1219	JQ900151	**100%**	**97.2%**	95.3%	95.0%	89.5%	95.2%	83.1%	94.4%
CS560-961	JQ900143	**99.8%**	96.8%	95.1%	94.8%	89.3%	95.0%	82.9%	94.2%
CS561-964	JQ900144	**99.8%**	96.8%	95.1%	94.7%	89.3%	95.0%	82.9%	94.2%
CS18-266	JQ900168	**98.9%**	**97.2%**	95.5%	94.0%	90.6%	95.7%	83.9%	95.1%
CS25-273	JQ900167	**98.9%**	**97.2%**	95.5%	94.0%	90.6%	95.7%	83.9%	95.1%
CS91-1291	JQ900166	**98.8%**	96.8%	95.3%	93.7%	90.4%	95.5%	83.7%	94.9%
CS37-295	JQ900169	**98.4%**	96.8%	95.1%	94.0%	90.4%	95.5%	83.5%	95.1%

Note: The percentage numbers in red refer to the similarities >97% between the sequences of ITS and reference GC-biased Genotypes #1–3, #7–10, and 12 of *O. sinensis*: Genotypes #1 (AB067721), #2 (MG770309), #3 (HM595984), #7 (AJ488254), #8 (GU246286), #9 (GU246288), #10 (GU246287), and #12 (GU246296) [9–10].

Table S2. The *O. sinensis* strains, GenBank accession numbers for the ITS nucleic acid sequences corresponding to the GenBank accession numbers for the MAT1-1-1 and MAT1-2-1 proteins, and percent similarities *vs*. AT-biased Genotypes #4–6 and #15–17 of *O. sinensis*.

***O. sinensis* strain**	**ITS1-5.8S-ITS2**	**Percent similarity comparing the sequences of ITS and the reference AT-biased genotypes of *O. sinensis***					
		**#4**	**#5**	**#6**	**#15**	**#16**	**#17**
CS6-251	JQ900163	87.5%	85.4%	84.2%	88.4%	86.7%	86.8%
	JQ900180	89.9%	**97.2%**	92.3%	93.3%	93.1%	**98.3%**
CS34-291	JQ900162	88.6%	86.1%	85.5%	89.5%	87.2%	87.5%
	JQ900179	89.9%	**97.2%**	92.3%	93.3%	93.1%	**98.3%**
CS70-1208	JQ900146	88.6%	86.1%	85.5%	89.5%	87.2%	87.5%
	JQ900172	90.2%	**97.6%**	93.4%	93.5%	93.3%	**98.1%**
CS71-1220	JQ900152	88.6%	86.1%	85.5%	89.5%	87.2%	87.5%
	JQ900175	90.2%	**97.6%**	93.4%	93.5%	93.3%	**98.1%**
CS68-2-1228	JQ900157	88.6%	86.1%	85.5%	89.5%	87.2%	87.5%
	JQ900178	90.2%	**97.6%**	93.4%	93.5%	93.3%	**98.1%**
CS76-1284	JQ900159	88.6%	86.1%	85.5%	89.5%	87.2%	87.5%
CS68-2-1229	JQ900158	88.6%	86.1%	85.5%	89.5%	87.2%	87.5%
CS71-1218	JQ900150	88.6%	86.1%	85.5%	89.5%	87.2%	87.5%
CS71-1219	JQ900151	88.6%	86.1%	85.5%	89.5%	87.2%	87.5%
CS560-961	JQ900143	88.4%	85.9%	85.2%	89.3%	87.0%	87.4%
CS561-964	JQ900144	88.4%	85.9%	85.2%	89.3%	87.0%	87.4%
CS18-266	JQ900168	88.3%	87.2%	85.5%	90.6%	86.8%	88.6%
CS25-273	JQ900167	88.3%	87.2%	85.5%	90.6%	86.8%	88.6%
CS91-1291	JQ900166	88.3%	87.0%	85.2%	90.4%	86.7%	88.4%
CS37-295	JQ900169	87.7%	86.7%	86.0%	90.0%	86.3%	88.1%

Note: The percentage numbers in red refer to the similarities >97% between the sequences of ITS and reference AT-biased Genotypes #4–6 and #15–17 of *O. sinensis*: Genotypes #4 (AB067744), #5 (AB067740), #6 (KJ720572), #15 (KT232017), #16 (KT232019), #and #17 (KT232010) [9–10].

Table S3. Amino acids are scaled on the basis of the general chemical characteristics of their side chains for ProtScale analysis (https://web.expasy.org/protscale/) to predict the hydrophobicity and secondary structures (α-helices, β-sheets, β-turns, and coils) of the proteins.

Amino acid		hydropathy index *	α-Helix	β-Sheet	β-Turn	Coil	Physicochemical property
Asp, D	Aspartic acid	-3.500	0.924	0.541	1.197	1.197	Acidic
Glu, E	Glutamic acid	-3.500	1.504	0.567	1.149	0.761	Acidic
Ile, I	Isoleucine	4.500	1.003	1.799	0.240	0.886	Aliphatic
Val, V	Valine	4.200	0.990	1.965	0.387	0.772	Aliphatic
Leu, L	Leucine	3.800	1.236	1.261	0.670	0.810	Aliphatic
Ala, A	Alanine	1.800	1.489	0.709	0.788	0.824	Aliphatic
Phe, F	Phenylalanine	2.800	1.195	1.393	0.624	0.797	Aromatic
Trp, W	Tryptophan	-0.900	1.090	1.306	0.546	0.941	Aromatic
Tyr, Y	Tyrosine	-1.300	0.787	1.266	0.795	1.109	Aromatic
His, H	Histidine	-3.200	1.003	0.863	0.970	1.068	Basic
Lys, K	Lysine	-3.900	1.172	0.721	1.302	0.897	Basic
Arg, R	Arginine	-4.500	1.224	0.920	0.912	0.893	Basic
Cys, C	Cysteine	2.500	0.966	1.191	0.965	0.953	with polar neutral side chains
Met, M	Methionine	1.900	1.363	1.210	0.436	0.810	with polar neutral side chains
Ser, S	Serine	-0.800	0.739	0.928	1.316	1.130	with polar neutral side chains
Thr, T	Threonine	-0.700	0.785	1.221	0.739	1.148	with polar neutral side chains
Asn, N	Asparagine	-3.500	0.772	0.604	1.572	1.167	with polar neutral side chains
Gln, Q	Glutamine	-3.500	1.164	0.840	0.997	0.947	with polar neutral side chains
Gly, G	Glycine	-0.400	0.510	0.657	1.860	1.251	Unique amino acid
Pro, P	Proline	-1.600	0.492	0.354	1.415	1.540	Unique amino acid

Note: An amino acid scale is defined at https://web.expasy.org/protscale/ by a numerical value assigned to each type of amino acid. The most frequently used scales are the hydrophobicity or hydrophilicity scales and the secondary structure conformational parameter scales, but many other scales exist, which are based on the different chemical and physical properties of the amino acids. The ExPASy ProtScale program provides 57 predefined scales based on the literature [74]. *, Hydropathy index [73]; the larger the value, the stronger the hydrophobicity; negative values indicate hydrophilicity.

Table S4. Similarity of the 4 pairs of primers that were used to amplify the ITS sequences of the *O. sinensis* strains [36] compared with the sequences of the representative GC- and AT-biased genotypes of *O. sinensis*.

***O. sinensis* genotype** and **accession number**	**Percent similarity/percent coverage of primer sequences aligned with the sequences of *O. sinensis* genotypes**							
	**Fungal universal primers**		**Specific for Group A ITS sequences**		**Specific for Group C ITS sequences**		**for amplifying 5.8S gene sequences**	
	**ITS5(F)**	**ITS4(R)**	**GAF**	**GAR**	**GCF**	**GCR**	**5.8S-F**	**5.8S-R**
#1 AB067721	**100%/100%**	**100%/100%**	**100%/100%**	**100%/100%**	92%/89%	93%/56%	95%/100%	93%/95%
#2 MG770309	—/—	—/—	—/—	—/—	—/—	—/—	95%/100%	93%/64%
#3 HM595984	—/—	45%/100%	86%/100%	96%/100%	92%/95%	—/—	95%/100%	93%/64%
#7 AJ488254	—/—	35%/100%	100%/68%	100%/48%	100%/68%	—/—	95%/100%	93%/64%
#8 GU246286	—/—	—/—	100%/77%	81%/100%	88%/93%	85%/80%	100%/62%	93%/82%
#9 GU246288	—/—	40%/100%	**100%/100%**	100%/48%	100%/43%	100%/40%	94%/85%	93%/68%
#10 GU246287	—/—	—/—	95%/100%	100%/74%	100%/25%	100%/28%	94%/81%	94%/73%
#11 JQ695935	—/—	**100%/100%**	100%/55%	100%/48%	92%/89%	—/—	95%/100%	93%/95%
#12 GU246296	—/—	100%/95%	**100%/100%**	81%/91%	92%/89%	100%/40%	95%/100%	93%/95%
#13 KT339190	**100%/100%**	**100%/100%**	**100%/100%**	81%/100%	92%/89%	100%/36%	95%/100%	100%/73%
#14 KT339178	**100%/100%**	**100%/100%**	100%/78%	**100%/100%**	100%/50%	93%/56%	95%/100%	93%/95%
#4 AB067744	**100%/100%**	**100%/100%**	100%/64%	87%/63%	92%/95%	100%/68%	95%/100%	100%/82%
#5 AB067740	100%/95%	—/—	100%/41%	100%/52%	**100%/100%**	**100%/100%**	**100%/100%**	95%/100%
#6 KJ720572	32%/100%	35%/100%	92%/55%	100%/26%	**100%/100%**	100%/60%	**100%/100%**	100%/50%
#15 KT232017	32%/100%	**100%/100%**	100%/64%	100%/52%	92%/96%	**100%/100%**	95%/100%	**100%/100%**
#16 KT232019	32%/100%	**100%/100%**	100%/64%	86%/78%	**100%/100%**	94%/68%	**100%/100%**	100%/82%
#17 KT232010	32%/100%	**100%/100%**	100%/64%	100%/52%	**100%/100%**	**100%/100%**	**100%/100%**	95%/100%

Note: Four pairs of primers were designed/selected and used by Li *et al.* [36] for amplification of ITS sequences for phylogenetic and genotypical determination of multiple *O. sinensis* genotypes: ITS5(F)/ITS4(R) shown in purple, GAF/GCF in green, GCF/GCR in blue, and 5.8S-F/R in pink.

1. The fungal universal primers **ITS5(F)** (GGAAGTAAAAGTCGTAACAAGG) and **ITS4(R)** (TCCTCCGCTTATTGATATGC) were originally described by Thomas J. White *et al*. [118];
2. The primers **GAF** (TCCCAAACCCCCTGCGAACACC) and **GAR** (AGGTCAACTGGAGGGTGTGGTGGTTTC) were designed to preferentially amplify GC-biased Group A sequences;
3. The primers **GCF** (TAGCAGTTGCCTTAGCGGGACCGCCCTA) and **GCR** (AATCCGAGGTTAACTAAAAGGCGTA) were designed to preferentially amplify AT-biased Group B sequences;
4. The primers **5.8S-F** (ACTTTTAACAACGGATCTCTT) and **5.8S-R** (AAGATAACGCTCGGATAAGCAT) were designed from the conserved 5.8S rRNA region and therefore are expected to amplify all paralogous ITS copies.

"—/—"indicates that no sequence identity or sequence coverage data were available for alignment of the primer sequence with the corresponding *O. sinensis* genotype sequence. Genotypes #1−3 and #7−14 are GC-biases, whereas Genotypes #4−6 and #15−17 are AT-biases.

[REFERENCES]

1. Zhu, J.-S.; Halpern, G.M.; Jones, K. The scientific rediscovery of a precious ancient Chinese herbal regimen: *Cordyceps sinensis*: Part I. *J. Altern. Complem. Med.* **1998a**, *4*, 289–303. https://doi.org/10.1089/acm.1998.4.3-289.
2. Zhu, J.-S.; Halpern, G.M.; Jones, K. The scientific rediscovery of an ancient Chinese herbal medicine: *Cordyceps sinensis*: Part II. *J. Altern. Complem. Med.* **1998b**, *4*, 429–457. https://doi.org/10.1089/acm.1998.4.429.

3. Zhu, J.-S.; Li, C.-L.; Tan, N.-Z.; Berger, J.L.; Prolla, T.A. Combined use of whole-gene expression profiling technology and mouse lifespan test in anti-aging herbal product study. In Proceedings of the 2011 New TCM Products Innovation and Industrial Development Summit, Hangzhou, China, 27 November **2011**; pp. 443–448. Available online: https://xueshu.baidu.com/usercenter/paper/show?paperid=08341c17fa58c8f85584b92572b90f75&site=xueshu_se (accessed on 30 January 2025).
4. Song, L.-R.; Hong, X.; Ding, X.-L.; Zang, Z.-Y. A comprehensive dictionary of modern pharmacy of Chinese traditional medicine. People's Medical Press, Beijing. 2001; pp 733–737.
5. China Ministry of Agriculture and Rural Affairs. Announcement (No. 15 of 2021) of National Forestry and Grassland Administration: List of National Key Protected Wild Plants. 7 September **2021**. Available online: https://m.163.com/dy/article/HHCVOJPU055360T7.html (accessed on 3 May 2025).
6. Ren, Y.; Wan, D.-G.; Lu, X.-M.; Guo, J.-L. The study of scientific name discussion for TCM Cordyceps. *LisShenzhen Med. Mater. Medica Res.* **2013**, *24*, 2211−2212.
7. Zhang, Y.-J.; Zhang, S.; Li, Y.-L.; Ma, S.-L.; Wang, C.-S.; Xiang, M.-C.; Liu, X.; An, Z.-Q.; Xu, J.-P.; Liu, X.-Z. Phylogeography and evolution of a fungal–insect association on the Tibetan Plateau. *Mol. Ecol.* **2014**, *23*, 5337−5355. https://doi.org/10.1111/mec.12940.
8. Lu, H.-L.; St. Leger, R.J. Chapter Seven—Insect Immunity to Entomopathogenic Fungi. In *Advances in Genetics*; Lovett, B., St. Leger, R.J., Eds.; Academic Press: Cambridge, MA, USA, **2016**, *94*, 251–285.
9. Zhu, J.-S.; Li, Y.-L. A Precious Transitional Chinese Medicine, *Cordyceps sinensis*: Multiple heterogeneous *Ophiocordyceps sinensis* in the insect–fungi complex. Lambert Academic Publishing, Saarbrüchen. Germany, **2017**.
10. Li, Y.-L.; Li, X.-Z.; Yao, Y.-S.; Xie, W.-D.; Zhu, J.-S. Molecular identification of *Ophiocordyceps sinensis* genotypes and the indiscriminate use of the Latin name for the multiple genotypes and the natural insect–fungi complex. *Am. J. BioMed. Sci.* **2022**, *14*, 115–135. https://doi.org/10.5099/aj220300115.
11. Li, M.-M.; Zhang, J.-H.; Qin, Q.-L.; Zhang, H.; Li, X.; Wang, H.-T.; Meng, Q. Transcriptome and Metabolome Analyses of *Thitarodes xiaojinensis* in Response to *Ophiocordyceps sinensis* Infection. *Microorganisms.* **2023a**, *11*, 2361. https://doi.org/10.3390/microorganisms11092361.
12. Li, Y.-L.; Gao, L.; Yao, Y.-S.; Wu, Z.-M.; Lou, Z.-Q.; Xie, W.-D.; Wu, J.-Y.; Zhu, J.-S. Altered GC- and AT-biased genotypes of *Ophiocordyceps sinensis* in the stromal fertile portions and ascospores of natural *Cordyceps sinensis*. *PLoS ONE.* **2023d**, *18*, e0286865. https://doi.org/10.1371/journal.pone.0286865.
13. Li, Y.-L.; Li, X.-Z.; Yao, Y.-S.; Wu, Z.-M.; Gao, L.; Tan, N.-Z.; Lou, Z.-Q.; Xie, W.-D.; Wu, J.-Y.; Zhu, J.-S. Differential cooccurrence of multiple genotypes of *Ophiocordyceps sinensis* in the stromata, stromal fertile portion (ascocarps) and ascospores of natural *Cordyceps sinensis*. *PLoS ONE.* **2023e**, *18*, e0270776. https://doi.org/10.1371/journal.pone.0270776.
14. Bushley, K.E.; Li, Y.; Wang, W.-J.; Wang, X.-L.; Jiao, L.; Spatafora, J.W.; Yao, Y.-J. Isolation of the MAT1-1 mating type idiomorph and evidence for selfing in the Chinese medicinal fungus *Ophiocordyceps sinensis*. *Fungal Biol.* **2013**, *117*, 599–610. https://doi.org/10.1016/j.funbio.2013.06.001.
15. Li, C.-L. A study of *Tolypocladium sinense* C.L. Li. sp. nov. and cyclosporin production. *Acta Mycol. Sin.* **1988**, *7*, 93−98.
16. Dai, R.-Q.; Lan, J.-L.; Chen, W.-H.; Li, X.-M.; Chen, Q.-T.; Shen, C.-Y. Discovery of a new fungus *Paecilomyces hepiali* Chen & Dai. *Acta Agricult. Univ. Pekin.* **1989**, *15*, 221−224.
17. Dai, R.-Q.; Li, X.-M.; Shao, A.-J.; Lin, S.-F.; Lan, J.-L.; Chen, W.-H.; Shen, C.-Y. Nomenclatural validation of *Paecilomyces hepiali*. *Mycosystema.* **2008**, *27*, 641–644.
18. Liu, X.-J.; Guo, Y.-L.; Yu, Y.-X.; Zeng, W. Isolation and identification of the anamorph of *Cordyceps sinensis* fungus. *Acta Mycol. Sin.* **1989**, *8*, 35–40.
19. Kinjo, N.; Zang, M. Morphological and phylogenetic studies on *Cordyceps sinensis* distributed in southwestern China. *Mycoscience.* **2001**, *42*, 567–574. https://doi.org/10.1007/BF02460956.
20. Jiang, Y.; Yao, Y.-J. A review for the debating studies on the anamorph of *Cordyceps sinensis*. *Mycosistema.* **2003**, *22*, 161–176.
21. Chen, Y.-Q.; Hu, B.; Xu, F.; Zhang, W.; Zhou, H.; Qu, L.-H. Genetic variation of *Cordyceps sinensis*, a fruit-body-producing entomopathogenic species from different geographical regions in China. *FEMS Microbiol. Lett.* **2004**, *230*, 153–158. https://doi.org/10.1016/S0378-1097(03)00889-9.
22. Chen, C.-S.; Hseu, R.-S.; Huang, C.-T. Quality Control of *Cordyceps sinensis* Teleomorph, Anamorph, and Its Products, Chapter 12. In *Quality Control of Herbal Medicines and Related Areas*; Shoyama, Y., Ed.; InTech: Rijeka, Croatia, **2011**; pp.

223–238. Available online: www.intechopen.com (accessed on 3 May 2024).
23. Stensrud, Ø.; Hywel-Jones, N.L.; Schumacher, T. Towards a phylogenetic classification of *Cordyceps*: ITS nrDNA sequence data confirm divergent lineages and paraphyly. *Mycol. Res.* **2005**, *109*, 41–56. https://doi.org/10.1017/s095375620400139x.
24. Stensrud, Ø.; Schumacher, T.; Shalchian-Tabrizi, K.; Svegardenib, I.B.; Kauserud, H. Accelerated nrDNA evolution and profound AT bias in the medicinal fungus *Cordyceps sinensis*. *Mycol. Res.* **2007**, *111*, 409–415. https://doi.org/10.1016/j.mycres.2007.01.015.
25. Leung, P.-H.; Zhang, Q.-X.; Wu, J.-Y. Mycelium cultivation, chemical composition and antitumour activity of a *Tolypocladium* sp. fungus isolated from wild *Cordyceps sinensis*. *J. Appl. Microbiol.* **2006**, *101*, 275–283. https://doi.org/10.1111/j.1365-2672.2006.02930.x.
26. Zhu J-S, Guo Y-L, Yao Y-S, Zhou Y-J, Lu J-H, Qi Y, Chen W, Zheng T-Y, Zhang L, Wu Z-M, Zhang L-J, Liu X-J, Yin W-T. Maturation of *Cordyceps sinensis* associates with co-existence of *Hirsutella sinensis* and *Paecilomyces hepiali* DNA and dynamic changes in fungal competitive proliferation predominance and chemical profiles. *J. Fungal Res*. **2007**, 5(4), 214–224.
27. Zhu, J.-S.; Gao, L.; Li, X.-H.; Yao, Y.-S.; Zhou, Y.-J.; Zhao, J.-Q.; Zhou, Y.-J. Maturational alterations of oppositely orientated rDNA and differential proliferations of CG:AT-biased genotypes of *Cordyceps sinensis* fungi and *Paecilomyces hepiali* in natural *C.* sinensis. *Am. J. Biomed. Sci.* **2010**, *2*, 217–238. https://doi.org/10.5099/aj100300217.
28. Yang, J.-L.; Xiao, W.; He, H.-X.; Zhu, H.-X.; Wang, S.-F.; Cheng, K.-D.; Zhu, P. Molecular phylogenetic analysis of *Paecilomyces hepiali* and *Cordyceps sinensis*. *Acta Pharmaceut. Sinica.* **2008**, *43*, 421–426.
29. Yang, J.-Y.; Tong, X.-X.; He, C.-Y.; Bai, J.; Wang, F.; Guo, J.-L. Comparison of endogenetic microbial community diversity between wild *Cordyceps sinensis*, artificial *C. sinensis* and habitat soil. *Chin. J. Chin. Mater. Medica.* **2021**, *46*, 3106–3115.
30. Xiao, W.; Yang, J.-P.; Zhu, P.; Cheng, K.-D.; He, H.-X.; Zhu, H.-X.; Wang, Q. Non-support of species complex hypothesis of *Cordyceps sinensis* by targeted rDNA-ITS sequence analysis. *Mycosystema.* **2009**, *28*, 724–730.
31. Zhang, Y.-J.; Sun, B.-D.; Zhang, S.; Wàngmŭ Liu, X.-Z.; Gong, W.-F. Mycobiotal investigation of natural *Ophiocordyceps sinensis* based on culture-dependent investigation. *Mycosistema.* **2010a**, *29*, 518–527. https://api.semanticscholar.org/CorpusID:88267953.
32. Zhang, Y.-J.; Zhang, S.; Wàngmŭ; Bai, F.-Y.; Liu, X.-Z. High Diversity of the Fungal Community Structure in Naturally-Occurring *Ophiocordyceps sinensis*. *PLoS ONE.* **2010b**, 5(12), e15570. doi:10.1371/journal.pone.0015570.
33. Zhang, S.-W.; Cen, K.; Liu, Y.; Zhou, X.-W.; Wang, C.-S. Metatranscriptomics analysis of the fruiting caterpillar fungus collected from the Qinghai-Tibetan plateau. *Sci. Sinica Vitae.* **2018**, *48*, 562–570. https://engine.scichina.com/publisher/scp/journal/SSV/48/5.
34. Barseghyan, G.S.; Holliday, J.C.; Price, T.C.; Madison, L.M.; Wasser, S.P. Growth and cultural-morphological characteristics of vegetative mycelia of medicinal caterpillar fungus *Ophiocordyceps sinensis* G.H. Sung et al. (Ascomycetes) Isolates from Tibetan Plateau (P. R. China). *Intl. J. Med. Mushrooms.* **2011**, *13*, 565–581. https://doi.org/10.1615/intjmedmushr.v13.i6.90.
35. Gao, L., Li, X.-H., Zhao, J.-Q., Lu, J.-H., Zhao, J.-G., Zhu, J.-S. Maturation of *Cordyceps sinensis* associates with alterations of fungal expressions of multiple *Ophiocordyceps sinensis* mutants with transition and transversion point mutations in stroma of *Cordyceps sinensis*. *Beijing Da Xue Xue Bao*. **2012**, *44*, 454–463. doi: 10.3969/j.issn.1671-167X.2012.03.025.
36. Li, Y.; Jiao, L.; Yao, Y.-J. Non-concerted ITS evolution in fungi, as revealed from the important medicinal fungus *Ophiocordyceps sinensis*. *Mol. Phylogenet. Evol.* **2013**, *68*, 373–379. https://doi.org/10.1016/j.ympev.2013.04.010.
37. Li, Y.-L.; Yao, Y.-S.; Zhang, Z.-H.; Xu, H.-F.; Liu, X.; Ma, S.-L.; Wu, Z.-M.; Zhu, J.-S. Synergy of fungal complexes isolated from the intestines of *Hepialus lagii* larvae in increasing infection potency. *J. Fungal Res.* **2016b**, *14*, 96–112.
38. Li, X.-Z., Li, Y.-L., Yao, Y.-S., Xie, W.-D., Zhu, J.-S. Further discussion with Li *et al*. (2013, 2019) regarding the "ITS pseudogene hypothesis" for *Ophiocordyceps sinensis*. *Mol. Phylogenet. Evol.* **2020b**, *146*, 106728. https://doi.org/10.1016/j.ympev.2019.106728.
39. Meng, Q.; Yu, H.-Y.; Zhang, H.; Zhu, W.; Wang, M.-L.; Zhang, J.-H.; Zhou, G.-L.; Li, X.; Qin, Q.-L.; Hu, S.-N.; et al. Transcriptomic insight into the immune defenses in the ghost moth, *Hepialus xiaojinensis*, during an *Ophiocordyceps sinensis* fungal infection. Insect Biochem. *Mol. Biol.* **2015**, *64*, 1–15. https://doi.org/10.1016/j.ibmb.2015.06.014.
40. Xia, F.; Liu, Y.; Shen, G.-L.; Guo, L.-X.; Zhou, X.-W. Investigation and analysis of microbiological communities in natural *Ophiocordyceps sinensis*. *Can. J. Microbiol.* **2015**, *61*, 104–111. https://doi.org/10.1139/cjm-2014-0610.

41. Xia, F.; Liu, Y.; Guo, L.-X.; Shen, G.-L.; Lin, J.; Zhou, X.-W. Pyrosequencing analysis revealed complex endogenetic microorganism community from natural DongChong XiaCao and its microhabitat. *BMC Microbiol*. **2016**, *16*, 196. DOI 10.1186/s12866-016-0813-5.
42. Guo, M.-Y.; Liu, Y.; Gao, Y.-H.; Jin, T.; Zhang, H.-B.; Zhou, X.-W. Identification and bioactive potential of endogenetic fungi isolated from medicinal caterpillar fungus *Ophiocordyceps sinensis* from Tibetan Plateau. *Int. J. Agric. Biol.* **2017**, *19*, 307–313. https://doi.org/10.17957/IJAB/15.0281.
43. Zhong, X.; Gu, L.; Wang, H.-Z.; Lian, D.-H.; Zheng, Y.-M.; Zhou, S.; Zhou, W.; Gu, J.; Zhang, G.; Liu, X. Profile of *Ophiocordyceps sinensis* transcriptome and differentially expressed genes in three different mycelia, sclerotium and fruiting body developmental stages. *Fungal Biol.* **2018**, *122*, 943–951. https://doi.org/10.1016/j.funbio.2018.05.011.
44. Kang, Q.; Zhang, J.; Chen, F.; Dong, C.; Qin, Q.; Li, X.; Wang, H.; Zhang, H.; Meng, Q. Unveiling mycoviral diversity in *Ophiocordyceps sinensis* through transcriptome analyses. *Front. Microbiol.* **2024**, *15*, 1493365. https://doi.org/10.3389/fmicb.2024.1493365.
45. Wei, X.-L.; Yin, X.-C.; Guo, Y.-L.; Shen, N.-Y.; Wei, J.-C. Analyses of molecular systematics on *Cordyceps sinensis* and its related taxa. *Mycosystema.* **2006**, *25*, 192–202.
46. Wei, J.-C.; Wei, X.-L.; Zheng, W.-F.; Guo, W.; Liu, R.-D. Species identification and component detection of *Ophiocordyceps sinensis* cultivated by modern industry. *Mycosystema.* **2016**, *35*, 404–410.
47. Lu, D. Western records and studies of the Chinese caterpillar fungus to the beginning of the 20th century. *J. Fungal. Res.* **2014**, *12*, 233–244. https://doi.org/10.13341/j.jfr.2014.0043.
48. Sung, G.-H.; Hywel-Jones, N.L.; Sung, J.-M.; Luangsa-ard, J.J.; Shrestha, B.; Spatafora, J.W. Phylogenetic classification of *Cordyceps* and the clavicipitaceous fungi. *Stud. Mycol.* **2007**, *57*, 5–59. https://doi.org/10.3114/sim.2007.57.01.
49. Zhang, Y.-J.; Li, E.-W.; Wang, C.-S.; Li, Y.-L.; Liu, X.-Z. *Ophiocordyceps sinensis*, the flagship fungus of China: Terminology, life strategy and ecology. *Mycol.* **2012**, *3*, 2–10. https://doi.org/10.1080/21501203.2011.654354.
50. Wang, Y.; Stata, M.; Wang, W.; Stajich, J.E.; White, M.M.; Moncalvo, J.M. Comparative genomics reveals the core gene toolbox for the fungus-insect symbiosis. *mBio.* **2018**, *9*, e00636-e18. https://doi.org/10.1128/mBio.00636-18.
51. Zhang, S.; Zhang, Y.-J.; Shrestha, B.; Xu, J.-P.; Wang, C.-S.; Liu, X.-Z. *Ophiocordyceps sinensis* and *Cordyceps militaris*: Research advances, issues and perspectives. *Mycosystema.* **2013**, *32*, 577–597.
52. Hawksworth, D.L.; Crous, P.W.; Redhead, S.A.; Reynolds, D.R.; Samson, R.A.; Seifert, K.A.; Taylor, J.W.; Wingfield, M.J.; Abaci, Ö.; Aime, C.; et al. The Amsterdam declaration on fungal nomenclature. *IMA Fungus.* **2011**, *2*, 105–112. https://doi.org/10.5598/imafungus.2011.02.01.14.
53. Taylor JW. One Fungus = One Name: DNA and fungal nomenclature twenty years after PCR. *IMA Fungus*. **2011**, *2(2),* 113–120. doi:10.5598/imafungus.2011.02.02.01.
54. Yao, Y.-S.; Zhu, J.-S. Indiscriminate use of the Latin name for natural *Cordyceps sinensis* and *Ophiocordyceps sinensis* fungi. *Chin. J. Chin. Mater. Med*. **2016**, *41*(7), 1316–1366.
55. Turgeon, B.G.; Yoder, O.C. Proposed nomenclature for mating type genes of filamentous ascomycetes. *Fungal Genet. Biol.* **2000**, *31*, 1–5. https://doi.org/10.1006/fgbi.2000.1227.
56. Debuchy, R.; Turgeo, B.G. Mating-Type Structure, Evolution, and Function in Euascomycetes. In *Growth, Differentiation and Sexuality*; Kües, U., Fischer, R., Eds.; Springer: Berlin/Heidelberg, Germany, 2006; pp. 293–323.
57. Jones, S.K.; Bennett, R.J. Fungal mating pheromones: Choreographing the dating game. *Fungal Genet. Biol.* **2011**, *48*, 668–676. https://doi.org/10.1016/j.fgb.2011.04.001.
58. Zheng, P.; Wang, C.-S. Sexuality Control and Sex Evolution in Fungi. *Sci. Sin. Vitae.* **2013**, *43*, 1090–1097. https://api.semanticscholar.org/CorpusID:87624278.
59. Wilson, A.M.; Wilken, P.M.; van der Nest, M.A.; Steenkamp, E.T.; Wingfield, M.J.; Wingfield, B.D. Homothallism: An umbrella term for describing diverse sexual behaviours. *IMA Fungus.* **2015**, *6*, 207–214. https://doi.org/10.5598/imafungus.2015.06.01.13.
60. Sun, S.; Coelho, M.A.; David-Palma, M.; Priest, S.J.; Heitman, J. The evolution of sexual reproduction and the mating-type locus: Links to pathogenesis of *Cryptococcus* human pathogenic fungi. *Annu. Rev. Genet.* **2019**, *53*, 417–444. https://doi.org/10.1146/annurev-genet-120116-024755.
61. Ramšak, B.; Kűck, U.; Hofmann, E. The mating type transcription factor MAT1-1-1 from the fungal human pathogen *Aspergillus fumigatus*: Synthesis, purification, and crystallization of the DNA binding domain. *bioRxiv.* **2021**. https://doi.org/10.1101/2021.12.13.472399.

62. Ramšak, B.; Kück, U. The *Penicillium chrysogenum* tom1 gene a major target of transcription factor MAT1-1-1 encodes a nuclear protein involved in sporulation. *Front. Fungal Bio.* **2022**, *3*, 937023. https://doi.org/10.3389/ffunb.2022.937023.
63. Metin, B.; Findley, K.; Heitman, J. The mating type locus (MAT) and sexual reproduction of *Cryptococcus heveanensis*: Insights into the evolution of sex and sex-determining chromosomal regions in fungi. *PLoS Genet.* **2010**, *6*, e1000961. https://doi.org/10.1371/journal.pgen.1000961.
64. Hu, X.; Zhang, Y.-J.; Xiao, G.-H.; Zheng, P.; Xia, Y.-L.; Zhang, X.-Y.; St Leger, R.J.; Liu, X.-Z.; Wang, C.-S. Genome survey uncovers the secrets of sex and lifestyle in caterpillar fungus. *Chin. Sci. Bull.* **2013**, *58*, 2846–2854. https://doi.org/10.1007/s11434-013-5929-5.
65. Li, X.-Z.; Li, Y.-L.; Liu, W.; Zhu, J.-S. Altered stereostructures of the DNA-binding domains of variant mating proteins of *Ophiocordyceps sinensis* and the wild insect–fungal complex. *Biol. (Basel).* **2026**, *15(2)*, 186. https://doi.org/10.3390/biology15020186.
66. Li, X.; Wang, F.; Liu, Q.; Li, Q.-P.; Qian, Z.-M.; Zhang, X.-L.; Li, K.; Li, W.-J.; Dong, C.-H. Developmental transcriptomics of Chinese cordyceps reveals gene regulatory network and expression profiles of sexual development-related genes. *BMC Genom.* **2019**, *20*, 337. https://doi.org/10.1186/s12864-019-5708-z.
67. Li, X.-Z.; Li, Y.-L.; Zhu, J.-S. Differential transcription of mating-type genes during sexual reproduction of natural *Cordyceps sinensis*. *Chin. J. Chin. Mater. Medica.* **2023c**, *48*, 2829–2840. https://doi.org/10.19540/j.cnki.cjcmm.20230213.102.
68. Li, X.-Z.; Xiao, M.-J.; Li, Y.-L.; Gao, L.; Zhu, J.-S. Mutations and differential transcription of mating-type and pheromone receptor genes in *Hirsutella sinensis* and the natural *Cordyceps sinensis* insect–fungi complex. *Biol.* **2024b**, *13*, 632. https://doi.org/10.3390/biology13080632.
69. Li, X.-Z.; Li, Y.-L.; Zhu, J.-S. Three-dimensional structural heteromorphs of mating-type proteins in *Hirsutella sinensis* and the natural *Cordyceps sinensis* insect–fungal complex. *J. Fungi.* **2025**, *11*, 244. https://doi.org/10.3390/jof11040244.
70. Zhang, Y.-J.; Xu, L.-L.; Zhang, S.; Liu, X.-Z.; An, Z.-Q.; Wàngmǔ Guo, Y.-L. Genetic diversity of *Ophiocordyceps sinensis*, a medicinal fungus endemic to the Tibetan Plateau: Implications for its evolution and conservation. *BMC Evol. Biol.* **2009**, *9*, 290. doi:10.1186/1471-2148-9-290.
71. Zhang, S.; Zhang, Y.-J.; Liu, X.-Z.; Wen, H.-A.; Wang, M.; Liu, D.-S. Cloning and analysis of the MAT1-2-1 gene from the traditional Chinese medicinal fungus *Ophiocordyceps sinensis*. *Fungal Biol.* **2011**, *115*, 708–714. https://doi.org/10.1016/j.funbio.2011.05.004.
72. Zhang, S.; Zhang, Y.-J. Molecular evolution of three protein-coding genes in the Chinese caterpillar fungus *Ophiocordyceps sinensis*. *Microbiol. China.* **2015**, *42*, 1549–1560.
73. Kyte, J.; Doolittle, R.F. A simple method for displaying the hydropathic character of a protein. *J. Mol. Biol.* **1982**, *157*, 105–132. https://doi.org/10.1016/0022-2836(82)90515-0.
74. Li, X.-Z.; Li, Y.-L.; Wang, Y.-N.; Zhu, J.-S. Translations of mutant repetitive genomic sequences in *Hirsutella sinensis* and changes in secondary structures and functional specifications of the encoded proteins. *Int. J. Mol. Sci.* **2024a**, *25*, 11178. https://doi.org/10.3390/ijms252011178.
75. Baxevanis, A.D.; Bryant, S.H.; Landsman, D. Homology model building of the HMG-1 box structural domain. *Nucleic Acids Res.* **1995**, *23*, 1019–1029. https://doi.org/10.1093/nar/23.6.1019.
76. Thapar, R. Structure-specific nucleic acid recognition by L-motifs and their diverse roles in expression and regulation of the genome. *Biochim. Biophys. Acta.* **2015**, *1849*, 677–687. https://doi.org/10.1016/j.bbagrm.2015.02.006.
77. Bittrich S.; Segura J.; Duarte J.M.; Burley S.K.; Rose Y. RCSB protein Data Bank: exploring protein 3D similarities via comprehensive structural alignments. *Bioinformatics*, **2024**, *40*(6), btae370. https://doi.org/10.1093/bioinformatics/btae370.
78. Zhang, Y.; Skolnick J. TM-align: a protein structure alignment algorithm based on the TM-score. *Nucleic Acids Research*, **2005**, *33*(7): 2302–2309. DOI: 10.1093/nar/gki524.
79. Smith, T.F.; Waterman, M.S. Identification of common molecular subsequences**.** *J. Mol. Biol*. **1981**, *147*(1):195–197. doi:10.1016/0022-2836(81)90087-5.
80. Li, Y.; Hsiang, T.; Yang, R.-H.; Hu, X.-D.; Wang, K.; Wang, W.-J.; Wang, X.-L.; Jiao, L.; Yao, Y.-J. Comparison of different sequencing and assembly strategies for a repeat-rich fungal genome, *Ophiocordyceps sinensis*. *J. Microbiol. Methods.* **2016a**, *128*, 1–6. https://doi.org/10.1016/j.mimet.2016.06.025.
81. Jin, L.-.Q.; Xu, Z.-.W.; Zhang, B.; Yi, M.; Weng, C.-.Y.; Lin, S.; Wu, H.; Qin, X.-T.; Xu, F.; Teng, Y.; Yuan, S.-J.; Liu, Z.-Q.; Zheng, Y.-G. Genome sequencing and analysis of fungus *Hirsutella sinensis* isolated from *Ophiocordyceps sinensis*. *AMB Expr***. 2020,** *10*, 105. https://doi.org/10.1186/s13568-020-01039-x.

82. Liu, J.; Guo, L.-N.; Li, Z.-W.; Zhou, Z.; Li, Z.; Li, Q.; Bo, X.-C.; Wang, S.-Q.; Wang, J.-L.; Ma, S.-C.; Zheng J, Yang Y. Genomic analyses reveal evolutionary and geologic context for the plateau fungus *Ophiocordyceps sinensis*. *Clin. Med.* **2020**, *15*, 107–119. https://doi.org/10.1186/s13020-020-00365-3.
83. Shu, R.-H.; Zhang, J.-H.; Meng, Q.; Zhang, H.; Zhou, G.-L.; Li, M.-M.; Wu, P.-P.; Zhao, Y.-N.; Chen, C.; Qin, Q.-L. A new high-quality draft genome assembly of the Chinese cordyceps *Ophiocordyceps sinensis*. *Genome Biol. Evol.* **2020**, *12*, 1074–1079. https://doi.org/10.1093/gbe/evaa112.
84. Rao, Z.; Han, R.; Sun, T.; Li, M.' Wang, Y.; Xu, J.; Li, Z.; Cao, L. Whole genome sequencing and resequencing provide insight into the genetic structure of wild Ophiocordyceps sinensis. *BMC Genomics*. **2025,** *26*(1):976. doi: 10.1186/s12864-025-12177-6.
85. Martin, T.; Lu, S.-W.; van Tilbeurgh, H.; Ripoll, D.R.; Dixelius, C.; Dixelius, C.; Turgeon, B.G.; Debuchy, R. Tracing the Origin of the Fungal a1 Domain Places Its Ancestor in the HMG-Box Superfamily, Implication for Fungal Mating-Type Evolution. *PLoS ONE.* **2010**, *5*, e15199. https://doi.org/10.1371/journal.pone.0015199.
86. Czaja, W.; Miller, K.Y.; Miller, B.L. Complex mechanisms regulate developmental expression of the matA (HMG) mating type gene in homothallic *Aspergillus* nidulans. *Genetics*. **2011**, *189*, 795-808. doi: 10.1534/genetics.111.131458.
87. Angelini, R.M.D.M., Rotolo, C., Pollastro, S., Faretra, F. Molecular analysis of the mating type (*MAT1*) locus in strains of the heterothallic ascomycete *Botrytis cinerea*. *Plant Pathol*. **2016**, *65*, 1221-1400. https://doi.org/10.1111/ppa.12509.
88. Poveda-Huertes, D., Matic, S., Marada, A., Habernig, L., Licheva, M., Myketin, L., Gilsbach, R., Tosal-Castano S, Papinski D, Mulica P, Kretz O, Kücükköse C, Taskin AA, Hein L, Kraft C, Büttner S, Meisinger C, Vögtle FN. An Early mtUPR: Redistribution of the Nuclear Transcription Factor Rox1 to Mitochondria Protects against Intramitochondrial Proteotoxic Aggregates. *Mol Cell*. **2020**, *77(1),* 180-188. doi: 10.1016/j.molcel.2019.09.026.
89. Hansen, F.T., Madsen, C.K., Nordland, A.M., Grasser, M., Merkle, T., Grasser, K.D. A novel family of plant DNA-binding proteins containing both HMG-box and AT-rich interaction domains. *Biochem*. **2008**; *47*, 13207–13214. doi: 10.1021/bi801772k.
90. Rajewska, M., Wegrzyn, K., Konieczny, I. AT-rich region and repeated sequences - the essential elements of replication origins of bacterial replicons. *FEMS Microbiol. Rev*. **2012**, *36(2),* 408–434. doi: 10.1111/j.1574-6976.2011.00300.x.
91. Jackson, D.; Lawson, T.; Villafane, R.; Gary, L. Modeling the structure of yeast MATα1, An HMG-Box motif with a C-terminal helical extension. *Open J. Biophys.* **2013**, *3*, 1–12. https://doi.org/10.4236/ojbiphy.2013.31001.
92. Zheng, P.; Xia, Y.-L.; Zhang, S.-W.; Wang, C.-S. Genetics of *Cordyceps* and related fungi. *Appl. Microbiol. Biotechnol*. **2013a**, *97*, 2797–2804. doi: 10.1007/s00253-013-4771-7.
93. Kim, H.-K.; Jo, S.-M.; Kim, G.-Y.; Kim, D.-W.; Kim, Y.-K.; Yun, S.-H. A large-scale functional analysis of putative target genes of mating-type loci provides insight into the regulation of sexual development of the cereal pathogen *Fusarium graminearum*. *PLoS Genet.* **2015**, *11*, e1005486. https://doi.org/10.1371/journal.pgen.1005486.
94. Ramšak, B.; Markau, J.; Pazen, T.; Dahlmann, T.A.; Krappmann, S.; Kűck, U. The master regulator MAT1-1-1 of fungal mating binds to its targets via a conserved motif in the human pathogen *Aspergillus fumigatus*. *G3 Genes Genom. Genet.* **2020**, *11*, jkaa012. https://doi.org/10.1093/g3journal/jkaa012.
95. Yamamoto, A.; Ando, Y.; Yoshioka, K.; Saito, K.; Tanabe, T.; Shirakawa, H.; Yoshida, M. Difference in affinity for DNA between HMG proteins 1 and 2 determined by surface plasmon resonance measurements. *J. Biochem.* **1997**, *122*, 586–594. https://doi.org/10.1093/oxfordjournals.jbchem.a021793.
96. Ait Benkhali, J.; Coppin, E.; Brun, S.; Peraza-Reyes, L.; Martin, T.; Dixelius, C.; Lazar, N.; van Tilbeurgh, H.; Debuchy, R. A Network of HMG-box Transcription Factors Regulates Sexual Cycle in the Fungus *Podospora anserina*. *PLoS Genet.* **2013**, *9*, e1003642. https://doi.org/10.1371/journal.pgen.1003642.
97. Balasubramanian, B.; Lowry, C.V.; Zitomer, R.S. The Rox1 repressor of the *Saccharomyces cerevisiae* hypoxic genes is a specific DNA-binding protein with a high-mobility-group motif. *Mol. Cell Biol.* **1993**, *13*, 6071–6078. https://doi.org/10.1128/mcb.13.10.6071-6078.1993.
98. Zitomer, R.S.; Limbach, M.P.; Rodriguez-Torres, A.M.; Balasubramanian, B.; Deckert, J.; Snow, P.M. Approaches to the study of Rox1 repression of the hypoxic genes in the yeast *Saccharomyces cerevisiae*. *Methods* **1997**, *11*, 279–288. https://doi.org/10.1006/meth.1996.0422.
99. Zheng, Q.; Hou, R.; Zhang, J.-Y.; Ma, J.; Ma, J.-W.; Wu, Z.-S.; Wang, G.-H.; Wang, C.-F.; Xu, J.-R. The MAT locus genes play different roles in sexual reproduction and pathogenesis in *Fusarium graminearum*. *PLoS ONE.* **2013b**, *8*, e66980. https://doi.org/10.1371/journal.pone.0066980; Erratum in *PLoS ONE*. **2015**, *10*, e0131623.

https://doi.org/10.1371/journal.pone.0131623.
100. Kastaniotis, A.J.; Zitomer, R.S. Oxygen Dependent Repression in Yeast. In *Rox1 Mediated Repression*; Advances in Experimental Medicine and Biology; Springer Nature: Cham, Switzerland, 2000; Vol. 475, pp. 185–195. https://doi.org/10.1007/0-306-46825-5_18.
101. Kües, U.; Casselton, L.A. The origin of multiple mating types in mushrooms. *J. Cell Sci.* **1993**, *104*, 227–230. https://doi.org/10.1242/jcs.104.2.227.
102. Asante-Owusu, R.N.; Banham, A.H.; Böhnert, H.U.; Mellor, E.J.C.; Casselton, L.A. Heterodimerization between two classes of homeodomain proteins in the mushroom *Coprinus cinereus* brings together potential DNA-binding and activation domains. *Gene.* **1996**, *172*, 25–31. https://doi.org/10.1016/0378-1119(96)00177-1.
103. Jacobsen, S.; Wittig, M.; Pöggeler, S. Interaction Between Mating-Type Proteins from the Homothallic Fungus *Sordaria macrospora*. *Curr. Genet.* **2002**, *41*, 150–158. https://doi.org/10.1007/s00294-002-0276-0.
104. Hancock, S.P.; Cascio, D.; Johnson, R.C. Cooperative DNA binding by proteins through DNA shape complementarity. *Nucleic Acids Res.* **2019**, *47*, 8874–8887. https://doi.org/10.1093/nar/gkz642.
105. Mao, X.-M.; Zhao, S.-M.; Cao, L.; Yan, X.; Han, R.-C. The morphology observation of *Ophiocordyceps sinensis* from different origins. *J. Environ. Entomol.* **2013**, *35*, 343–353. doi: 10.3969/j.issn.1674-0858.2013.03.11.
106. Wang, Y.-B., Wang, Y., Fan, Q., Duan, D-E., Zhang, G.-D., Dai6, R.-Q., Dai, Y.-D., Zeng, W.-B. Chen, Z.-H., Li, D.-D., Tang, D.-X., Xu, Z.-H., Sun, T., Nguyen, T.-T., Tran, N.-L., Dao, V.-M., Zhang, C.-M., Huang, L.-D., Liu, Y.-J., Zhang, X.-M., Yang, D.-R., Sanjuan, T., Liu, X.-Z., Yang, Z.-L., Yu, H. Multigene phylogeny of the family Cordycipitaceae (Hypocreales): new taxa and the new systematic position of the Chinese cordycipitoid fungus *Paecilomyces hepiali*. *Fungal Diversity*. **2020**, *103*, 1–46. https://doi.org/10.1007/s13225-020-00457-3.
107. Li, Y.; Yang, R.-H.; Jiang, L.; Hu, X.-D.; Wu, Z.-J.; Yao, Y.-J. rRNA Pseudogenes in Filamentous Ascomycetes as Revealed by Genome Data. *G3-Genes Genom. Genet.* **2017**, *7*, 2695–2703. https://doi.org/10.1534/g3.117.044016.
108. Li, Y.; Jiang, L.; Wang, K.; Wu, H.-J.; Yang, R.-H.; Yan, Y.-J.; Bushley, K.E.; Hawksworth, D.L.; Wu, Z.-J.; Yao, Y.-J. RIP mutated ITS genes in populations of *Ophiocordyceps sinensis* and their implications for molecular systematics. *IMA Fungus.* **2020c**, *11*, 18. https://doi.org/10.1186/s43008-020-00040-0.
109. Holliday, J.; Cleaver, M. Medicinal value of the caterpillar fungi species of the genus *Cordyceps* (Fr.) Link (Ascomycetes). A review. *Int. J. Med. Mushrooms.* **2008**, *10*, 219–234. https://doi.org/10.1615/IntJMedMushr.v10.i3.30.
110. Stone, R. Improbable partners aim to bring biotechnology to a Himalayan kingdom. *Science.* **2010**, *327*, 940–941. https://doi.org/10.1126/science.327.5968.940.
111. Qin, Q.-L.; Zhou, G.-L.; Zhang, H.; Meng, Q.; Zhang, J.-H.; Wang, H.-T.; Miao, L.; Li, X. Obstacles and approaches in artificial cultivation of Chinese cordyceps. *Mycol.* **2018**, *9*, 7–9. https://doi.org/10.1080/21501203.2018.1442132.
112. Li, W.-J.; Xia, J.-M.; Li, Q.-P.; Zhang, Z.-Y.; Zhang, W.-W.; Dong, C.-H.; Wei, J.-C.; Liu, X.-Z. Developmental recording of the ghost-moth larvae after ex situ infection by *Ophiocordyceps sinensis*. *Sci. China Life Sci.* **2020a**, *63*, 1093–1095. https://doi.org/10.1007/s11427-020-1686-7; Erratum in *Sci. China Life Sci*. **2023**, *66*, 892. https://doi.org/10.1007/s11427-022-2232-5.
113. He, Z.; Ye, M.; Wu, H.; Liang, D.; Huan, J.; Yao, Y.; Wu, X.; Luo, X. The Conservation Crisis of *Ophiocordyceps sinensis*: Strategies, Challenges, and Sustainable Future of Artificial Cultivation. *J. Fungi.* **2025**, 11, 892. https://doi.org/10.3390/jof11120892.
114. Liu, T-L.; Wu, L.-J.; Zhao, X.-Y.; Tao, X.-Y.; Qiu, Y.-J.; Li, R.; Zheng, Y.-J.; Liu, L.; Tian, M.-L. Regulation of strain infectivity in *Ophiocordyceps sinensis*: insights into fungal-insect symbiosis and artificial cultivation. *Symbiosis.* **2026**. https://doi.org/10.1007/s13199-026-01155-5.
115. Sun, T.; Zou, W.-Q.; Luo, R.; Li, C.-P.; Zhang, C.,-M.; Yu, H. Compositional and functional diversities of core microbial communities in wild and artificial *Ophiocordyceps sinensis*. *Int. Microbiol*. **2023**, *26*, 791–806. https://doi.org/10.1007/s10123-023-00333-5
116. Khalid, M.Z.; Zheng, X.; Han, R.-C.; Cao, L. *Fusarium pseudonygamai* promotes blastospore transformation in *Ophiocordyceps sinensis*: Insights into microbial interaction and key mechanisms. *J. Fungi*. **2025**, *11*(10): 746. https://doi.org/10.3390/jof11100746.
117. Bao, X.-W.; Wang, Q.-H.; Li, T.; Li, Y.; Bian, Z.-Y.; Liu, S.-J.; He, L.-Y.; Niu, S.-Q.; Guo, J.-L. *Ophiocordyceps sinensis*-induced changes in *Thitarodes xiaojinensis*: from intestinal barrier destruction, microbiome dysbiosis to immune responses at the molecular level. *BMC Biol*. **2025**, *23*, 183. https://doi.org/10.1186/s12915-025-02277-w

118. Bennett, R.J.; Johnson, A.D. Completion of a parasexual cycle in *Candida albicans* by induced chromosome loss in tetraploid strains. *EMBO J.* **2003**, *22*, 2505–2515. https://doi.org/10.1093/emboj/cdg235.
119. Sherwood, R.K.; Bennett, R.J. Fungal meiosis and parasexual reproduction--lessons from pathogenic yeast. *Curr. Opin. Microbiol.* **2009**, *12*, 599–607. https://doi.org/10.1016/j.mib.2009.09.005.
120. Seervai, R.N.H.; Jones, S.K.; Hirakawa, M.P.; Porman, A.M.; Bennett, R.J. Parasexuality and ploidy change in *Candida tropicalis*. *Eukaryot. Cell.* **2013**, *12*, 1629–1640. https://doi.org/10.1128/EC.00128-13.
121. Nakamura, N.; Tanaka, C.; Takeuchi-Kaneko, Y. Transmission of antibiotic-resistance markers by hyphal fusion suggests partial presence of parasexuality in the root endophytic fungus *Glutinomyces brunneus*. *Mycol. Progress.* **2019**, *18*, 453–462. https://doi.org/10.1007/s11557-018-1455-9.
122. Du, X.-H.; Wu, D.-M.; Kang, H.; Wang, H.-C.; Xu, N.; Li, T.-T.; Chen, K.-L. Heterothallism and potential hybridization events inferred for twenty-two yellow morel species. *IMA Fungus.* **2020**, *11*, 4. https://doi.org/10.1186/s43008-020-0027-1; Correction to *IMA Fungus*. **2022**, *13*, 10. https://doi.org/10.1186/s43008-022-00096-0.
123. Hėnault, M.; Marsit, S.; Charron, G.; Landry, C.R. The effect of hybridization on transposable element accumulation in an undomesticated fungal species. *eLife.* **2020**, *9*, e60474. https://doi.org/10.7554/eLife.60474.
124. Samarasinghe, H.; You, M.; Jenkinson, T.S.; Xu, J.-P.; James, T.Y. Hybridization Facilitates Adaptive Evolution in Two Major Fungal Pathogens. *Genes.* **2020**, *11*, 101. https://doi.org/10.3390/genes11010101.
125. Mishra, A.; Forche, A.; Anderson, M.Z. Parasexuality of Candida Species. *Front. Cell. Infect. Microbiol.* **2021**, *11*, 796929. https://doi.org/10.3389/fcimb.2021.796929.
126. Steensels, J.; Gallone, B.; Verstrepen, K.J. Interspecific hybridization as a driver of fungal evolution and Adaptation. *Nat. Rev. Microbiol.* **2021**, *19*, 485–500. https://doi.org/10.1038/s41579-021-00537-4.
127. Zhang, J.-S.; Zhang, W.; Wu, X.-D.; Fu, W.-D.; Yang, C.-Y.; Long, N.-N. Insights into the Mycosphere Fungal Community and Its Association with Nucleoside Accumulation in *Ophiocordyceps sinensis*. *J. Fungi.* **2025**, *11*, 696. https://doi.org/10.3390/jof11100696.
128. Wang, Q.-H.; Wang, Y.-P.; Li, T.; Bao, X.-W.; He, L.-Y.; Liu, L.; Liu, S.-J.; Bai, J.; Zhang, H.; Niu, S.-Q.; Guo, J.-L. The interplay between the formation of Chinese cordyceps and the characteristics of soil properties and microbial network. *Microbiol. Spectr*. **2025**, *13*, e327724. https://journals.asm.org/doi/10.1128/spectrum.03277-24.
129. Yang, J.-Y.; Tong, X.-X.; He, C.-Y.; Bai, J.; Wang, F.; Guo, J.-L. Comparison of endogenetic microbial community diversity between wild *Cordyceps sinensis*, artificial *C. sinensis* and habitat soil. *China J. Chin. Mater. Medica.* **2021**, 46, 3106–3115. DOI:10.19540/j.cnki.cjcmm.20210226.103.
130. Deleage, G.; Roux, B. An algorithm for protein secondary structure prediction based on class prediction. *Protein Eng. Des. Sel.* **1987**, *1*, 289–294. https://doi.org/10.1093/protein/1.4.289.
131. Gasteiger, E.; Hoogland, C.; Gattiker, A.; Duvaud, S.; Wilkins, M.R.; Appel, R.D.; Bairoch, A. Protein Identification and Analysis Tools on the ExPASy Server, Chapter 52. In *The Proteomics Protocols Handbook*; Walker, J.M., Ed.; Humana Press: Totowa, NJ, USA, **2005**; pp. 571–607.
132. Peters, C.; Elofsson, A. Why is the biological hydrophobicity scale more accurate than earlier experimental hydrophobicity scales? *Proteins.* **2014**, *82*, 2190–2198. https://doi.org/10.1002/prot.24582.
133. Simm, S.; Einloft, J.; Mirus, O.; Schleiff, E. 50 years of amino acid hydrophobicity scales, revisiting the capacity for peptide classification. *Biol. Res.* **2016**, *49*, 31. https://doi.org/10.1186/s40659-016-0092-5.
134. Jumper, J.; Evans, R.; Pritzel, A.; Green, T.; Figurnov, M.; Ronneberger, O.; Tunyasuvunakool, K.; Bates, R.; Žídek, A.; Potapenko, A.; Bridgland, A.; Meyer, C.: Kohl, S,A,A,; Ballard, A.J.; Cowie, A.; Romera-Paredes, B.; Nikolov, S.; Jain, R.; Adler, J.; Back, T.; Petersen, S.; Reiman, D.; Clancy, E.; Zielinski, M.; Steinegger, M.; Pacholska, M.; Berghammer, T.; Bodenstein; S.; Silver, D.; Vinyals, O.; Senior, A.W.; Kavukcuoglu, K.; Kohli, P.; Hassabis, D. Highly accurate protein structure prediction with AlphaFold. *Nature.* **2021**, *596*, 583–589. https://doi.org/10.1038/s41586-021-03819-2.
135. Tunyasuvunakool, K.; Adler, J.; Wu, Z.; Green, T.; Zielinski, M.; Žídek, A.; Bridgland, A.; Cowie, A.; Meyer, C.; Laydon, A.; Velankar S, Kleywegt GJ, Bateman A, Evans R, Pritzel A, Figurnov M, Ronneberger O, Bates R, Kohl SAA, Potapenko A, Ballard AJ, Romera-Paredes B, Nikolov S, Jain R, Clancy E, Reiman D, Petersen S, Senior AW, Kavukcuoglu K, Birney E, Kohli P, Jumper J, Hassabis D. Highly accurate protein structure prediction for the human proteome. *Nature.* **2021**, *596*, 590–596. https://doi.org/10.1038/s41586-021-03828-1.
136. David, A.; Islam, S.; Tankhilevich, E.; Sternberg, M.J.E. The AlphaFold Database of Protein Structures, A Biologist's Guide. *J. Mol. Biol.* **2022**, *434*, 167336. https://doi.org/10.1016/j.jmb.2021.167336.

137. Monzon, V.; Haft, D.H.; Bateman, A. Folding the unfoldable, using AlphaFold to explore spurious proteins. *Bioinform. Adv.* **2022**, *1*, vbab043. https://doi.org/10.1093/bioadv/vbab043.
138. Rettie, S.A.; Campbell, K.V.; Bera, A.K.; Kang, A.; Kozlov, S.; De La Cruz, J.; Adebomi, V.; Zhou, G.; DiMaio, F.; Ovchinnikov, S.; et al. Cyclic peptide structure prediction and design using AlphaFold. *bioRxiv.* **2023**, *26*: 2023.02.25.529956. https://doi.org/10.1101/2023.02.25.529956.
139. Xu, T.; Xu, Q.; Li, J.-Y. Toward the appropriate interpretation of Alphafold2. *Front. Artif. Intell.* **2023b**, *6*, 1149748. https://doi.org/10.3389/frai.2023.1149748.
140. Abramson, J.; Adler, J.; Dunger, J.; Evans, R.; Green, T.; Pritzel, A.; Ronneberger, O.; Willmore, L.; Ballard, A.J.; Bambrick, J.; Bodenstein SW, Evans DA, Hung CC, O'Neill M, Reiman D, Tunyasuvunakool K, Wu Z, Žemgulytė A, Arvaniti E, Beattie C, Bertolli O, Bridgland A, Cherepanov A, Congreve M, Cowen-Rivers AI, Cowie A, Figurnov M, Fuchs FB, Gladman H, Jain R, Khan YA, Low CMR, Perlin K, Potapenko A, Savy P, Singh S, Stecula A, Thillaisundaram A, Tong C, Yakneen S, Zhong ED, Zielinski M, Žídek A, Bapst V, Kohli P, Jaderberg M, Hassabis D, Jumper JM. Accurate structure prediction of biomolecular interactions with AlphaFold 3. *Nature.* **2024**, *630*, 493–500. https://doi.org/10.1038/s41586-024-07487-w.
141. Varadi, M.; Bertoni, D.; Magana, P.; Paramval, U.; Pidruchna, I.; Radhakrishnan, M.; Tsenkov, M.; Nair, S.; Mirdita, M.; Yeo, J.; et al. AlphaFold Protein Structure Database in 2024, providing structure coverage for over 214 million protein sequences. *Nucleic Acids Res.* **2024**, *52*, D368–D375. https://doi.org/10.1093/nar/gkad1011.
142. Wroblewski, K.; Kmiecik, S. Integrating AlphaFold pLDDT Scores into CABS-flex for enhanced protein flexibility simulations. *Comput. Struct. Biotechnol. J.* **2024**, *30*, 4350–4356. https://doi.org/10.1016/j.csbj.2024.11.047.
143. Li, X.-Z.; Li, Y.-L.; Zhu, J.-S. Three-dimensional structural heteromorphs of mating-type proteins in *Hirsutella sinensis* and the natural *Cordyceps sinensis* insect–fungal complex. *J. Fungi.* **2025**, *11*, 244. https://doi.org/10.3390/jof11040244.
144. Yang, X.; Zhu, H.-Q.; Shi, L.-X.; Song, T.-G.; Gong, W.-B.; He, S.-M.; Shan, S.; Xu, C.-F.; Zhou, Z. AlphaFold-guided structural analyses of nucleosome binding proteins. *Nucleic Acids Res.* **2025**, *53*, gkaf735. https://doi.org/10.1093/nar/gkaf735.
145. Mariani, V.; Biasini, M.; Barbato, A.; Schwede, T. lDDT, a local superposition-free score for comparing protein structures and models using distance difference tests. *Bioinformatics.* **2013**, *29*, 2722–2728. https://doi.org/10.1093/bioinformatics/btt473.
